# *Aspilota*-group (Hymenoptera: Braconidae: Alysiinae) diversity in Mediterranean Natural Parks of Spain

**DOI:** 10.3897/BDJ.2.e1112

**Published:** 2014-07-21

**Authors:** Francisco Javier Peris-Felipo, Sergey A Belokobylskij, Jose Vicente Falcó-Garí, Ricardo Jiménez-Peydró

**Affiliations:** † Instituto Cavanilles de Biodiversidad y Biología Evolutiva. University of Valencia, Paterna (Valencia), Spain; ‡ Zoological Institute Russian Academy of Sciences, St. Petersburg, Russia

**Keywords:** Biodiversity, community, natural parks, Valencia, Aspilota, Braconidae

## Abstract

This work analyses the biodiversity of the *Aspilota*-group (Hymenoptera: Braconidae: Alysiinae) in three Mediterranean Natural parks: Natural Park of La Font Roja, Natural Park of Las Lagunas de la Mata-Torrevieja and Natural Park of La Tinença de Benifassà. Samples were carried out from April 2004 to December 2007. In total, 822 specimens, belonging to 52 species, were collected. Alpha, beta and gamma diversities were analysed, and the Tinença Park was proven to have higher diversity than the Font Roja and Torrevieja. Also, the structure of the *Aspilota*-group community was analysed.

## Introduction

Mediterranean ecosystems are very important in terms of biodiversity, and are thus considered hotspot areas (Myers et al. 2000). Landscapes and habitats grow in complexity over time, as a consequence of ecological processes. For example, Mediterranean forest landscapes rich in evergreen species frequently intersect with brushwood, pasture, farming and ranching areas. In close proximity to these areas, however, it is often possible to identify zones that have been reclaimed by highly diverse natural communities after the cessation of human intervention. Despite the huge resistance displayed by Mediterranean biotopes to human pressure, isolation and fragmentation are unavoidable (Pungetti 2003), resulting in the emergence of isolated patches in the landscape.

In land environments, the information provided by arthropods can be very valuable for the adoption of measures aimed at guaranteeing the diversity and welfare of protected forests (Pyle et al. 1981, Pearson and Cassola 1992, Kremen et al. 1993), especially insects with a high sensitivity to alterations in environmental resources and conditions. Parasitoids Hymenoptera of the Braconidae family, with around 40,000 catalogued species, are especially pertinent in this respect due to their particular biology (Wharton et al. 1997).

Braconidae are the second largest family within Hymenoptera; the majority of species are primary parasitoids of immature stages of Lepidoptera, Coleoptera and Diptera (Sharkey 1993). These wasps are of enormous ecological interest because of their role in controlling the population of phytophagous insects, causing direct effects in the host species’ population size and indirect effects on the diversity and survival of host plants (LaSalle and Gauld 1992). Additionally, they can indicate the presence or absence of said populations (Matthews 1974, LaSalle and Gauld 1992). Finally, some species can also be relevant from an economic point of view, because of their potential for pest control (González and Ruíz 2000).

Because of the type of relationship established between Braconidae populations and host species, and the effect that climatic factors and human activity pose upon this, we can consider that Braconidae (especially those adopting koinobiont strategies) are a valid parameter for the determination of human effects on these communities and the assessment of specific diversity within a region (González and Ruíz 2000).

The subfamily Alysiinae has 2,000 catalogued species worldwide classified in two tribes, Alysiini and Dacnusini (Yu et al. 2012). The Alysiini interact with a wide variety of cyclorrhapha hosts, often in humid habitats and ephemeral substrata, laying their eggs in the host’s larvae or eggs. Dacnusini are almost exclusively specialised for leaf and stem miners, such as Agromyzidae, Ephydridae and Chloropidae. Alysiini are distributed in all regions while Dacnusini are known mainly in the temperate and boreal regions of the Northern Hemisphere.

The *Aspilota*-group is a rather well differentiated group within the tribe *Alysiini* (van Achterberg 1988) with approximately 750 species described (Yu et al. 2012), including in the world fauna the genera *Adelphenaldis* Fischer, 2003, *Alithia* Cameron, 1906, *Aspilota* Foerster, 1862, *Carinthilota* Fischer, 1975, *Cubitalostigma* Fischer, 1998, *Dinostigma* Fischer, 1966, *Dinotrema* Foerster, 1862, *Eudinostigma* Tobias, 1986, *Grandilota* Fischer, 2002, *Leptotrema* van Achterberg, 1988, *Lysodinotrema* Fischer, 1995, *Neorthostigma* Belokobylskij, 1998, *Orthostigma* Ratzeburg, 1844, *Panerema* Foerster, 1862, *Pterusa* Fischer, 1958, *Regetus* Papp, 1999, and *Synaldis* Foerster, 1862.

Nevertheless, over the years, the genera included in this group have changed since, for example, van Achterberg (1988) did not consider *Synaldis* as a genus and included their species within the *Dinotrema* genus. However, later publications by Fischer (Fischer 1993a, Fischer 1993b), Belokobylskij (Belokobylskij 2002, Belokobylskij 2004a, Belokobylskij 2004b) and Tobias (Tobias 2003b, Tobias 2003a, Tobias 2004a, Tobias 2004b, Tobias 2006) considered *Synaldis* as a taxonomically different genus from *Dinotrema* due to the absence of the 2RS vein.

Although many diversity studies around Braconidae have been carried out worldwide, for example in Brazil (Cirelli and Penteado-Dias 2003, Scatolini and Penteado-Dias 2003), Venezuela (Briceño et al. 2007, Briceño et al. 2009) or in the Iberian Peninsula (Andorra, Spain and Portugal) (Falcó-Garí et al. 2006, Jiménez-Peydró and Peris-Felipo 2011, Peris-Felipo and Jiménez-Peydró 2011, Falcó-Garí et al. 2014), these communities have been insufficiently analysed. However, in recent years, some biodiversity studies have been carried out. These studies have increased the knowledge of this group with the description of 42 new European species (Peris-Felipo et al. 2012, Peris-Felipo et al. 2013a, Peris-Felipo et al. 2013b, Peris-Felipo et al. 2013c, Peris-Felipo et al. 2013d, Peris-Felipo and Belokobylskij 2013, Munk et al. 2013a, Munk et al. 2013b, Peris-Felipo et al. 2014).

Within this context, this work analyses alpha, beta and gamma diversity and community structure of the *Aspilota*-group in three Mediterranean Natural Parks of Valencia (Spain).

## Materials and methods

### *Area of Study*

Three natural parks in Comunidad Valenciana were selected: Natural Park of La Font Roja; Natural Park of Las Lagunas de la Mata-Torrevieja and Natural Park of La Tinença de Benifassà, each of which features peculiar microclimate conditions.

The Natural Park of La Font Roja is located to the north of Alicante province, and is known for its low level of anthropogenic disturbance. The park extends over 2,298 ha, with a maximum elevation of 1,356 m. The orientation of the hill range favours cool, moist winds from the northeast, resulting in rainfall retention. This fact, along with the steep slopes and the predominance of limestone, fosters the existence of different landscape units. Among these, deciduous forests, brushwood, scrub rock vegetation, pine forests and agricultural areas can be differentiated. In addition, each face experiences different climate conditions: the north face is classified as upper sub-humid, with annual rainfall between 600–1,000 mm; while the south face is dry, with annual rainfall between 350–600 mm. Due to high average temperatures throughout the year (15–20 °C), and the low average rainfall, the park is classified is dry and thermo-Mediterranean.

The Natural Park of Las Lagunas de la Mata-Torrevieja is located to the south of Alicante province, and extends over 3,700 ha, 2,100 of which are covered by water. The park is notable for its saline soils, extensive wild orchid population (*Orchis collina* Banks and Sol. ex Russell), differentiated areas of *Senecio auricula* Bourgeau ex Coss and salt marsh plants of the genus *Limonium*, reed and bulrush areas with abundant grass plants such as *Arthrocnemum* sp. and *Juncus* sp., and Mediterranean areas populated by *Quercus coccifera* L., *Pinus halepensis* Mill. and *Thymus* sp. The climate is arid with an annual rainfall below 300 mm and high temperatures.

The Natural Park of La Tinença de Benifassà is located to the north of Castellón province, and extends over approximately 25,814 ha. The park covers an extensive and well-preserved mountainous area, encompassing numerous and widely varied landscapes associated with medium and high-altitude Mediterranean regimes and hosting a high biodiversity of fauna and flora. It is possible to differentiate forests of *Pinus sylvestris* L., *Pinus uncinata* Mill. and *Fagus sylvatica* L., *Juniperus communis* L., and *Quercus ilex* L., etc., alternating with crops of *Prunus* sp., *Corylus* sp., etc. Climate conditions are continental humid, with annual average temperatures below 12 °C: freezing conditions are possible throughout most of the year. Rainfall varies in different zones according to topographical features, and the annual precipitation ranges from 600 to 1,000 l/m^2^. The park is contained within the supramediterranean bioclimate.

### *Sampling Design and Data Collection*

Sampling stage covered the period among April 2004 and December 2007. During this period, in each natural park, a Malaise trap to collected specimens was placed. Weekly, each area was visited to replace the collecting bottle. Specimens captured were preserved in 70% ethanol until final preparation.

Once separated, the specimens were determined by subfamily keys of van Achterberg (1990) to work only with Alysiinae specimens. Subsequently, the identification to genera was carried out using van Achterberg (1990) key. Finally, species identification was did it by Fischer (1993b), Fischer (2003), Fischer et al. (2008a), Fischer et al. (2008b) and Tobias' keys (Tobias et al. 1986). The studied specimens are deposited with a bar code labels in the Entomological Collection at the University of Valencia (Valencia, Spain; ENV). General distribution data were provide from Yu et al. (2012).

### *Data analysis*

Once the specimens of *Aspilota*-group had been identified, alpha, beta and gamma biodiversity indexes for each trap and habitat were calculated to gain insight into the richness, abundance, dominance and complementarity values of each area.

Alpha diversity reflects the richness in species of a homogeneous community. This sort of diversity was measured by taxa richness, abundance and dominance.

- Taxa richness: used for valuing richness of sampling areas. It was measured using the Margalef index, a measure of specific richness that transforms the number of species per sample into the proportion to which the species are added by expansion of the sample, establishing a functional relationship between number of species and total number of specimens (Moreno 2001).
- Species richness estimators: It was measured using Chao 2 to know what percentage of the total known species of possible species (Moreno 2001).
- Abundance: used for valuing faunal composition of a given area (Magurran 1988). This was undertaken using the Shannon-Weaver index because it measures equity, indicating the degree of uniformity in species representation (in order of abundance) while considering all samples. This index measures the average degree of uncertainty that predicts which species an individual randomly picked from a sample belongs to (Magurran 1988, Moreno 2001, Villarreal et al. 2004).
- Dominance: occurrence of genera or dominance value was calculated with the Simpson index, often used to measure species dominance values in a given community, with negative values thus representing equity. It measures the representativity of the most important species without considering the other species present. It expresses the probability that two individuals randomly picked from a sample will belong to the same species (Magurran 1988).
- Community structure: In order to complement the diversity analyses and enquire into community structure, *log-series*, *log-normal* and *broken-stick* models were also applied (Magurran 1988). The *log-series* model represents a community composed of a few abundant species and a high number of rare species. The *broken-stick* model refers to maximum occupation of an environment with equitable sharing of resources between species. Finally, the *log-normal* reflects an intermediate situation between the two (Soares et al. 2010). Using the data obtained from the parks, each of these models was applied to calculate the expected number of species, and log_2_ grouping species according to abundance (Magurran 1988, Tokeshi 1993, Krebs 1999). To test the significance of the models, the expected species values were compared with those of the observed species through chi-square analysis (Zar 1999).

Beta diversity is the degree of change or substitution in species composition between different communities within the same landscape. In order to measure beta diversity, Jaccard and Complementarity indexes were used and cluster analyses were also performed.

- Jaccard index: relates the total amount of shared species to the total amount of exclusive species. It is a qualitative coefficient, the interval of which will go from 0 when no species are shared between both sites to 1 when both sites have an identical composition (Moreno 2001, Villarreal et al. 2004).
- Complementarity index: indicates the degree of similarity in species composition and abundance between two or more communities (Moreno 2001, Villarreal et al. 2004).
- Cluster analysis: employed to calculate the degree of correlation based on similarity/dissimilarity. For the calculation of these values, statistics-processing software PAST was used (Hammer et al. 2001).

Finally, gamma diversity measurement indicates the diversity value of all environments under study, as expressed in the richness indexes for each area (alpha diversity) and the difference between them (beta diversity) (Schluter and Ricklefs 1993, Villarreal et al. 2004).

## Taxon treatments

### Adelphenaldis maxfischeri

Peris-Felipo, 2012

#### Materials

- **Type status:** Holotype. **Occurrence:** recordedBy: F. J. Peris-Felipo; individualCount: 1; sex: female; **Location:** country: Spain; stateProvince: Alicante; verbatimLocality: Alcoi, Natural Parl of Carrascal de La Font Roja; verbatimElevation: 1072; verbatimLatitude: 38°38'51''N; verbatimLongitude: 000°32'46''W; **Event:** samplingProtocol: Malaise trap; eventDate: 2007-06-25; **Record Level:** institutionCode: ENV
- **Type status:** Paratype. **Occurrence:** recordedBy: F. J. Peris-Felipo; individualCount: 1; sex: female; **Location:** country: Spain; stateProvince: Alicante; verbatimLocality: Alcoi, Natural Park of Carrascal de La Font Roja; verbatimElevation: 1072; verbatimLatitude: 38°38'51''N; verbatimLongitude: 000°32'46''W; **Event:** samplingProtocol: Malaise trap; eventDate: 2007-07-02; **Record Level:** institutionCode: ENV

#### Distribution

Spain.

### Aspilota anaphoretica

Fischer, 1973

#### Materials

- **Type status:** Other material. **Occurrence:** recordedBy: F. J. Peris-Felipo; individualCount: 1; sex: female; **Location:** country: Spain; stateProvince: Alicante; verbatimLocality: Alcoi, Natural Park of Carrascal de La Font Roja; verbatimElevation: 1072; verbatimLatitude: 38°38'51''N; verbatimLongitude: 000°32'46''W; **Event:** samplingProtocol: Malaise trap; eventDate: 2006-10-23; **Record Level:** institutionCode: ENV
- **Type status:** Other material. **Occurrence:** recordedBy: F. J. Peris-Felipo; individualCount: 1; sex: female; **Location:** country: Spain; stateProvince: Alicante; verbatimLocality: Alcoi, Natural Park of Carrascal de La Font Roja; verbatimElevation: 1072; verbatimLatitude: 38°38'51''N; verbatimLongitude: 000°32'46''W; **Event:** samplingProtocol: Malaise trap; eventDate: 2007-05-28; **Record Level:** institutionCode: ENV
- **Type status:** Other material. **Occurrence:** recordedBy: F. J. Peris-Felipo; individualCount: 2; sex: females; **Location:** country: Spain; stateProvince: Alicante; verbatimLocality: Alcoi, Natural Park of Carrascal de La Font Roja; verbatimElevation: 1072; verbatimLatitude: 38°38'51''N; verbatimLongitude: 000°32'46''W; **Event:** samplingProtocol: Malaise trap; eventDate: 2007-06-04; **Record Level:** institutionCode: ENV

#### Distribution

Austria, Greece, Hungary, Korea and Spain.

### Aspilota delicata

Fischer, 1973

#### Materials

- **Type status:** Other material. **Occurrence:** recordedBy: F. J. Peris-Felipo; individualCount: 1; sex: female; **Location:** country: Spain; stateProvince: Alicante; verbatimLocality: Alcoi, Natural Park of Carrascal de La Font Roja; verbatimElevation: 1072; verbatimLatitude: 38°38'51''N; verbatimLongitude: 000°32'46''W; **Event:** samplingProtocol: Malaise trap; eventDate: 2007-05-29; **Record Level:** institutionCode: ENV
- **Type status:** Other material. **Occurrence:** recordedBy: F. J. Peris-Felipo; individualCount: 2; sex: females; **Location:** country: Spain; stateProvince: Castellón; verbatimLocality: Pobla de Benifassà, Natural Park of Tinença de Benifassà; verbatimElevation: 662; verbatimLatitude: 40°39'22''N; verbatimLongitude: 000°9'25''W; **Event:** samplingProtocol: Malaise trap; eventDate: 2006-09-11; **Record Level:** institutionCode: ENV

#### Distribution

Austria, Greece, Hungary, Iran and Spain **(new record)**.

### Aspilota flagimilis

Fischer, 1996

#### Materials

- **Type status:** Other material. **Occurrence:** recordedBy: F. J. Peris-Felipo; individualCount: 2; sex: females; **Location:** country: Spain; stateProvince: Alicante; verbatimLocality: Alcoi, Natural Park of Carrascal de La Font Roja; verbatimElevation: 1072; verbatimLatitude: 38°38'51''N; verbatimLongitude: 000°32'46''W; **Event:** samplingProtocol: Malaise trap; eventDate: 2006-11-27; **Record Level:** institutionCode: ENV
- **Type status:** Other material. **Occurrence:** recordedBy: F. J. Peris-Felipo; individualCount: 1; sex: female; **Location:** country: Spain; stateProvince: Alicante; verbatimLocality: Alcoi, Natural Park of Carrascal de La Font Roja; verbatimElevation: 1072; verbatimLatitude: 38°38'51''N; verbatimLongitude: 000°32'46''W; **Event:** samplingProtocol: Malaise trap; eventDate: 2007-06-25; **Record Level:** institutionCode: ENV

#### Distribution

Spain.

### Aspilota insolita

(Tobias, 1962)

#### Materials

- **Type status:** Other material. **Occurrence:** recordedBy: F. J. Peris-Felipo; individualCount: 1; sex: female; **Location:** country: Spain; stateProvince: Castellón; verbatimLocality: Pobla de Benifassà, Natural Park of Tinença de Benifassà; verbatimElevation: 662; verbatimLatitude: 40°39'22''N; verbatimLongitude: 000°9'25''W; **Event:** samplingProtocol: Malaise trap; eventDate: 2006-03-21; **Record Level:** institutionCode: ENV
- **Type status:** Other material. **Occurrence:** recordedBy: F. J. Peris-Felipo; individualCount: 1; sex: female; **Location:** country: Spain; stateProvince: Castellón; verbatimLocality: Pobla de Benifassà, Natural Park of Tinença de Benifassà; verbatimElevation: 662; verbatimLatitude: 40°39'22''N; verbatimLongitude: 000°9'25''W; **Event:** samplingProtocol: Malaise trap; eventDate: 2006-06-12; **Record Level:** institutionCode: ENV

#### Distribution

Former Czechoslovakia, Hungary, Russia and Spain.

### Aspilota procreata

Fischer, 1976

#### Materials

- **Type status:** Other material. **Occurrence:** individualCount: 1; sex: female; **Location:** country: Spain; stateProvince: Alicante; locality: Torrevieja, Natural Park of Lagunas de La Mata-Torrevieja; verbatimElevation: 6 m; verbatimLatitude: 38°01'49''N; verbatimLongitude: 000°42'00''E; **Identification:** identifiedBy: F. J. Peris-Felipo; **Event:** samplingProtocol: Malaise trap; eventDate: 2004-05-04; **Record Level:** institutionCode: ENV
- **Type status:** Other material. **Occurrence:** individualCount: 2; sex: female; **Location:** country: Spain; stateProvince: Alicante; locality: Torrevieja, Natural Park of Lagunas de La Mata-Torrevieja; verbatimElevation: 6 m; verbatimLatitude: 38°01'49''N; verbatimLongitude: 000°42'00''E; **Identification:** identifiedBy: F. J. Peris-Felipo; **Event:** samplingProtocol: Malaise trap; eventDate: 2004-05-18; **Record Level:** institutionCode: ENV
- **Type status:** Other material. **Occurrence:** individualCount: 1; sex: female; **Location:** country: Spain; stateProvince: Alicante; locality: Torrevieja, Natural Park of Lagunas de La Mata-Torrevieja; verbatimElevation: 6 m; verbatimLatitude: 38°01'49''N; verbatimLongitude: 000°42'00''E; **Identification:** identifiedBy: F. J. Peris-Felipo; **Event:** samplingProtocol: Malaise trap; eventDate: 2004-05-25; **Record Level:** institutionCode: ENV
- **Type status:** Other material. **Occurrence:** individualCount: 3; sex: female; **Location:** country: Spain; stateProvince: Alicante; locality: Torrevieja, Natural Park of Lagunas de La Mata-Torrevieja; verbatimElevation: 6 m; verbatimLatitude: 38°01'49''N; verbatimLongitude: 000°42'00''E; **Identification:** identifiedBy: F. J. Peris-Felipo; **Event:** samplingProtocol: Malaise trap; eventDate: 2004-06-01; **Record Level:** institutionCode: ENV
- **Type status:** Other material. **Occurrence:** individualCount: 2; sex: males; **Location:** country: Spain; stateProvince: Alicante; locality: Torrevieja, Natural Park of Lagunas de La Mata-Torrevieja; verbatimElevation: 6 m; verbatimLatitude: 38°01'49''N; verbatimLongitude: 000°42'00''E; **Identification:** identifiedBy: F. J. Peris-Felipo; **Event:** samplingProtocol: Malaise trap; eventDate: 2004-06-01; **Record Level:** institutionCode: ENV
- **Type status:** Other material. **Occurrence:** individualCount: 1; sex: female; **Location:** country: Spain; stateProvince: Alicante; locality: Torrevieja, Natural Park of Lagunas de La Mata-Torrevieja; verbatimElevation: 6 m; verbatimLatitude: 38°01'49''N; verbatimLongitude: 000°42'00''E; **Identification:** identifiedBy: F. J. Peris-Felipo; **Event:** samplingProtocol: Malaise trap; eventDate: 2004-06-08; **Record Level:** institutionCode: ENV
- **Type status:** Other material. **Occurrence:** individualCount: 1; sex: male; **Location:** country: Spain; stateProvince: Alicante; locality: Torrevieja, Natural Park of Lagunas de La Mata-Torrevieja; verbatimElevation: 6 m; verbatimLatitude: 38°01'49''N; verbatimLongitude: 000°42'00''E; **Identification:** identifiedBy: F. J. Peris-Felipo; **Event:** samplingProtocol: Malaise trap; eventDate: 2004-06-15; **Record Level:** institutionCode: ENV
- **Type status:** Other material. **Occurrence:** individualCount: 1; sex: male; **Location:** country: Spain; stateProvince: Alicante; locality: Torrevieja, Natural Park of Lagunas de La Mata-Torrevieja; verbatimElevation: 6 m; verbatimLatitude: 38°01'49''N; verbatimLongitude: 000°42'00''E; **Identification:** identifiedBy: F. J. Peris-Felipo; **Event:** samplingProtocol: Malaise trap; eventDate: 2004-06-22; **Record Level:** institutionCode: ENV
- **Type status:** Other material. **Occurrence:** individualCount: 1; sex: female; **Location:** country: Spain; stateProvince: Alicante; locality: Torrevieja, Natural Park of Lagunas de La Mata-Torrevieja; verbatimElevation: 6 m; verbatimLatitude: 38°01'49''N; verbatimLongitude: 000°42'00''E; **Identification:** identifiedBy: F. J. Peris-Felipo; **Event:** samplingProtocol: Malaise trap; eventDate: 2004-12-14; **Record Level:** institutionCode: ENV
- **Type status:** Other material. **Occurrence:** individualCount: 1; sex: female; **Location:** country: Spain; stateProvince: Alicante; locality: Torrevieja, Natural Park of Lagunas de La Mata-Torrevieja; verbatimElevation: 6 m; verbatimLatitude: 38°01'49''N; verbatimLongitude: 000°42'00''E; **Identification:** identifiedBy: F. J. Peris-Felipo; **Event:** samplingProtocol: Malaise trap; eventDate: 2005-03-04; **Record Level:** institutionCode: ENV
- **Type status:** Other material. **Occurrence:** individualCount: 1; sex: male; **Location:** country: Spain; stateProvince: Alicante; locality: Torrevieja, Natural Park of Lagunas de La Mata-Torrevieja; verbatimElevation: 6 m; verbatimLatitude: 38°01'49''N; verbatimLongitude: 000°42'00''E; **Identification:** identifiedBy: F. J. Peris-Felipo; **Event:** samplingProtocol: Malaise trap; eventDate: 2005-03-23; **Record Level:** institutionCode: ENV
- **Type status:** Other material. **Occurrence:** individualCount: 1; sex: female; **Location:** country: Spain; stateProvince: Alicante; locality: Torrevieja, Natural Park of Lagunas de La Mata-Torrevieja; verbatimElevation: 6 m; verbatimLatitude: 38°01'49''N; verbatimLongitude: 000°42'00''E; **Identification:** identifiedBy: F. J. Peris-Felipo; **Event:** samplingProtocol: Malaise trap; eventDate: 2005-04-26; **Record Level:** institutionCode: ENV
- **Type status:** Other material. **Occurrence:** individualCount: 1; sex: female; **Location:** country: Spain; stateProvince: Alicante; locality: Torrevieja, Natural Park of Lagunas de La Mata-Torrevieja; verbatimElevation: 6 m; verbatimLatitude: 38°01'49''N; verbatimLongitude: 000°42'00''E; **Identification:** identifiedBy: F. J. Peris-Felipo; **Event:** samplingProtocol: Malaise trap; eventDate: 2005-12-12; **Record Level:** institutionCode: ENV
- **Type status:** Other material. **Occurrence:** individualCount: 1; sex: female; **Location:** country: Spain; stateProvince: Alicante; locality: Torrevieja, Natural Park of Lagunas de La Mata-Torrevieja; verbatimElevation: 6 m; verbatimLatitude: 38°01'49''N; verbatimLongitude: 000°42'00''E; **Identification:** identifiedBy: F. J. Peris-Felipo; **Event:** samplingProtocol: Malaise trap; eventDate: 2006-05-01; **Record Level:** institutionCode: ENV
- **Type status:** Other material. **Occurrence:** individualCount: 2; sex: females; **Location:** country: Spain; stateProvince: Alicante; locality: Torrevieja, Natural Park of Lagunas de La Mata-Torrevieja; verbatimElevation: 6 m; verbatimLatitude: 38°01'49''N; verbatimLongitude: 000°42'00''E; **Identification:** identifiedBy: F. J. Peris-Felipo; **Event:** samplingProtocol: Malaise trap; eventDate: 2006-06-06; **Record Level:** institutionCode: ENV
- **Type status:** Other material. **Occurrence:** individualCount: 1; sex: female; **Location:** country: Spain; stateProvince: Alicante; locality: Torrevieja, Natural Park of Lagunas de La Mata-Torrevieja; verbatimElevation: 6 m; verbatimLatitude: 38°01'49''N; verbatimLongitude: 000°42'00''E; **Identification:** identifiedBy: F. J. Peris-Felipo; **Event:** samplingProtocol: Malaise trap; eventDate: 2006-06-26; **Record Level:** institutionCode: ENV
- **Type status:** Other material. **Occurrence:** individualCount: 1; sex: male; **Location:** country: Spain; stateProvince: Alicante; locality: Torrevieja, Natural Park of Lagunas de La Mata-Torrevieja; verbatimElevation: 6 m; verbatimLatitude: 38°01'49''N; verbatimLongitude: 000°42'00''E; **Identification:** identifiedBy: F. J. Peris-Felipo; **Event:** samplingProtocol: Malaise trap; eventDate: 2007-03-20; **Record Level:** institutionCode: ENV
- **Type status:** Other material. **Occurrence:** individualCount: 1; sex: female; **Location:** country: Spain; stateProvince: Alicante; locality: Torrevieja, Natural Park of Lagunas de La Mata-Torrevieja; verbatimElevation: 6 m; verbatimLatitude: 38°01'49''N; verbatimLongitude: 000°42'00''E; **Identification:** identifiedBy: F. J. Peris-Felipo; **Event:** samplingProtocol: Malaise trap; eventDate: 2007-04-03; **Record Level:** institutionCode: ENV
- **Type status:** Other material. **Occurrence:** individualCount: 1; sex: female; **Location:** country: Spain; stateProvince: Alicante; locality: Torrevieja, Natural Park of Lagunas de La Mata-Torrevieja; verbatimElevation: 6 m; verbatimLatitude: 38°01'49''N; verbatimLongitude: 000°42'00''E; **Identification:** identifiedBy: F. J. Peris-Felipo; **Event:** samplingProtocol: Malaise trap; eventDate: 2007-04-16; **Record Level:** institutionCode: ENV
- **Type status:** Other material. **Occurrence:** individualCount: 1; sex: female; **Location:** country: Spain; stateProvince: Alicante; locality: Torrevieja, Natural Park of Lagunas de La Mata-Torrevieja; verbatimElevation: 6 m; verbatimLatitude: 38°01'49''N; verbatimLongitude: 000°42'00''E; **Identification:** identifiedBy: F. J. Peris-Felipo; **Event:** samplingProtocol: Malaise trap; eventDate: 2007-05-01; **Record Level:** institutionCode: ENV
- **Type status:** Other material. **Occurrence:** individualCount: 2; sex: females; **Location:** country: Spain; stateProvince: Alicante; locality: Torrevieja, Natural Park of Lagunas de La Mata-Torrevieja; verbatimElevation: 6 m; verbatimLatitude: 38°01'49''N; verbatimLongitude: 000°42'00''E; **Identification:** identifiedBy: F. J. Peris-Felipo; **Event:** samplingProtocol: Malaise trap; eventDate: 2007-05-08; **Record Level:** institutionCode: ENV
- **Type status:** Other material. **Occurrence:** individualCount: 1; sex: male; **Location:** country: Spain; stateProvince: Alicante; locality: Torrevieja, Natural Park of Lagunas de La Mata-Torrevieja; verbatimElevation: 6 m; verbatimLatitude: 38°01'49''N; verbatimLongitude: 000°42'00''E; **Identification:** identifiedBy: F. J. Peris-Felipo; **Event:** samplingProtocol: Malaise trap; eventDate: 2007-05-08; **Record Level:** institutionCode: ENV
- **Type status:** Other material. **Occurrence:** individualCount: 1; sex: female; **Location:** country: Spain; stateProvince: Alicante; locality: Torrevieja, Natural Park of Lagunas de La Mata-Torrevieja; verbatimElevation: 6 m; verbatimLatitude: 38°01'49''N; verbatimLongitude: 000°42'00''E; **Identification:** identifiedBy: F. J. Peris-Felipo; **Event:** samplingProtocol: Malaise trap; eventDate: 2007-05-15; **Record Level:** institutionCode: ENV
- **Type status:** Other material. **Occurrence:** individualCount: 3; sex: males; **Location:** country: Spain; stateProvince: Alicante; locality: Torrevieja, Natural Park of Lagunas de La Mata-Torrevieja; verbatimElevation: 6 m; verbatimLatitude: 38°01'49''N; verbatimLongitude: 000°42'00''E; **Identification:** identifiedBy: F. J. Peris-Felipo; **Event:** samplingProtocol: Malaise trap; eventDate: 2007-05-15; **Record Level:** institutionCode: ENV
- **Type status:** Other material. **Occurrence:** individualCount: 2; sex: females; **Location:** country: Spain; stateProvince: Alicante; locality: Torrevieja, Natural Park of Lagunas de La Mata-Torrevieja; verbatimElevation: 6 m; verbatimLatitude: 38°01'49''N; verbatimLongitude: 000°42'00''E; **Identification:** identifiedBy: F. J. Peris-Felipo; **Event:** samplingProtocol: Malaise trap; eventDate: 2007-05-29

#### Distribution

Austria, Czech Republic, Hungary and Spain **(new record)**.

### Aspilota propedaemon

Fischer, 1996

#### Materials

- **Type status:** Other material. **Occurrence:** individualCount: 1; sex: female; **Location:** country: Spain; stateProvince: Alicante; locality: Alcoi, Natural Park of Carrascal de La Font Roja; verbatimElevation: 1072 m; verbatimLatitude: 38°38'51''N; verbatimLongitude: 000°32'46''W; **Identification:** identifiedBy: F. J. Peris-Felipo; **Event:** samplingProtocol: Malaise trap; eventDate: 2004-05-06; **Record Level:** institutionCode: ENV
- **Type status:** Other material. **Occurrence:** individualCount: 1; sex: female; **Location:** country: Spain; stateProvince: Alicante; locality: Alcoi, Natural Park of Carrascal de La Font Roja; verbatimElevation: 1072 m; verbatimLatitude: 38°38'51''N; verbatimLongitude: 000°32'46''W; **Identification:** identifiedBy: F. J. Peris-Felipo; **Event:** samplingProtocol: Malaise trap; eventDate: 2006-12-04; **Record Level:** institutionCode: ENV
- **Type status:** Other material. **Occurrence:** individualCount: 1; sex: female; **Location:** country: Spain; stateProvince: Castellón; locality: Pobla de Benifassà, Natural Park of Tinença de Benifassà; verbatimElevation: 662 m; verbatimLatitude: 40°39'22''N; verbatimLongitude: 000°9'25''W; **Identification:** identifiedBy: F. J. Peris-Felipo; **Event:** samplingProtocol: Malaise trap; eventDate: 2005-03-29; **Record Level:** institutionCode: ENV
- **Type status:** Other material. **Occurrence:** individualCount: 1; sex: female; **Location:** country: Spain; stateProvince: Castellón; locality: Pobla de Benifassà, Natural Park of Tinença de Benifassà; verbatimElevation: 662 m; verbatimLatitude: 40°39'22''N; verbatimLongitude: 000°9'25''W; **Identification:** identifiedBy: F. J. Peris-Felipo; **Event:** samplingProtocol: Malaise trap; eventDate: 2006-06-12; **Record Level:** institutionCode: ENV
- **Type status:** Other material. **Occurrence:** individualCount: 1; sex: female; **Location:** country: Spain; stateProvince: Castellón; locality: Pobla de Benifassà, Natural Park of Tinença de Benifassà; verbatimElevation: 662 m; verbatimLatitude: 40°39'22''N; verbatimLongitude: 000°9'25''W; **Identification:** identifiedBy: F. J. Peris-Felipo; **Event:** samplingProtocol: Malaise trap; eventDate: 2006-06-19; **Record Level:** institutionCode: ENV
- **Type status:** Other material. **Occurrence:** individualCount: 1; sex: female; **Location:** country: Spain; stateProvince: Castellón; locality: Pobla de Benifassà, Natural Park of Tinença de Benifassà; verbatimElevation: 662 m; verbatimLatitude: 40°39'22''N; verbatimLongitude: 000°9'25''W; **Identification:** identifiedBy: F. J. Peris-Felipo; **Event:** samplingProtocol: Malaise trap; eventDate: 2006-08-20; **Record Level:** institutionCode: ENV
- **Type status:** Other material. **Occurrence:** individualCount: 1; sex: female; **Location:** country: Spain; stateProvince: Castellón; locality: Pobla de Benifassà, Natural Park of Tinença de Benifassà; verbatimElevation: 662 m; verbatimLatitude: 40°39'22''N; verbatimLongitude: 000°9'25''W; **Identification:** identifiedBy: F. J. Peris-Felipo; **Event:** samplingProtocol: Malaise trap; eventDate: 2005-03-23; **Record Level:** institutionCode: ENV
- **Type status:** Other material. **Occurrence:** individualCount: 1; sex: male; **Location:** country: Spain; stateProvince: Alicante; locality: Torrevieja, Natural Park of Lagunas de La Mata-Torrevieja; verbatimElevation: 6 m; verbatimLatitude: 38°01'49''N; verbatimLongitude: 000°42'00''W; **Identification:** identifiedBy: F. J. Peris-Felipo; **Event:** samplingProtocol: Malaise trap; eventDate: 2005-03-23; **Record Level:** institutionCode: ENV

#### Distribution

Spain.

### Aspilota propeminimam

Fischer, Torlos, Pardo & Asís, 2008

#### Materials

- **Type status:** Other material. **Occurrence:** individualCount: 1; sex: female; **Location:** country: Spain; stateProvince: Alicante; locality: Alcoi, Natural Park of Carrascal de La Font Roja; verbatimElevation: 1072 m; verbatimLatitude: 38°38'51''N; verbatimLongitude: 000°32'46''W; **Identification:** identifiedBy: F. J. Peris-Felipo; **Event:** samplingProtocol: Malaise trap; eventDate: 2007-06-18; **Record Level:** institutionCode: ENV
- **Type status:** Other material. **Occurrence:** individualCount: 1; sex: female; **Location:** country: Spain; stateProvince: Castellón; locality: Pobla de Benifassà, Natural Park of Tinença de Benifassà; verbatimElevation: 662 m; verbatimLatitude: 40°39'22''N; verbatimLongitude: 000°9'25''W; **Identification:** identifiedBy: F. J. Peris-Felipo; **Event:** samplingProtocol: Malaise trap; eventDate: 2005-05-23; **Record Level:** institutionCode: ENV
- **Type status:** Other material. **Occurrence:** individualCount: 1; sex: female; **Location:** country: Spain; stateProvince: Castellón; locality: Pobla de Benifassà, Natural Park of Tinença de Benifassà; verbatimElevation: 662 m; verbatimLatitude: 40°39'22''N; verbatimLongitude: 000°9'25''W; **Identification:** identifiedBy: F. J. Peris-Felipo; **Event:** samplingProtocol: Malaise trap; eventDate: 2006-03-14; **Record Level:** institutionCode: ENV
- **Type status:** Other material. **Occurrence:** individualCount: 2; sex: females; **Location:** country: Spain; stateProvince: Castellón; locality: Pobla de Benifassà, Natural Park of Tinença de Benifassà; verbatimElevation: 662 m; verbatimLatitude: 40°39'22''N; verbatimLongitude: 000°9'25''W; **Identification:** identifiedBy: F. J. Peris-Felipo; **Event:** samplingProtocol: Malaise trap; eventDate: 2006-04-17; **Record Level:** institutionCode: ENV
- **Type status:** Other material. **Occurrence:** individualCount: 4; sex: females; **Location:** country: Spain; stateProvince: Castellón; locality: Pobla de Benifassà, Natural Park of Tinença de Benifassà; verbatimElevation: 662 m; verbatimLatitude: 40°39'22''N; verbatimLongitude: 000°9'25''W; **Identification:** identifiedBy: F. J. Peris-Felipo; **Event:** samplingProtocol: Malaise trap; eventDate: 2006-06-05; **Record Level:** institutionCode: ENV
- **Type status:** Other material. **Occurrence:** individualCount: 1; sex: female; **Location:** country: Spain; stateProvince: Castellón; locality: Pobla de Benifassà, Natural Park of Tinença de Benifassà; verbatimElevation: 662 m; verbatimLatitude: 40°39'22''N; verbatimLongitude: 000°9'25''W; **Identification:** identifiedBy: F. J. Peris-Felipo; **Event:** samplingProtocol: Malaise trap; eventDate: 2006-07-17; **Record Level:** institutionCode: ENV
- **Type status:** Other material. **Occurrence:** individualCount: 1; sex: male; **Location:** country: Spain; stateProvince: Castellón; locality: Pobla de Benifassà, Natural Park of Tinença de Benifassà; verbatimElevation: 662 m; verbatimLatitude: 40°39'22''N; verbatimLongitude: 000°9'25''W; **Identification:** identifiedBy: F. J. Peris-Felipo; **Event:** samplingProtocol: Malaise trap; eventDate: 2006-11-27; **Record Level:** institutionCode: ENV
- **Type status:** Other material. **Occurrence:** individualCount: 1; sex: female; **Location:** country: Spain; stateProvince: Castellón; locality: Pobla de Benifassà, Natural Park of Tinença de Benifassà; verbatimElevation: 662 m; verbatimLatitude: 40°39'22''N; verbatimLongitude: 000°9'25''W; **Identification:** identifiedBy: F. J. Peris-Felipo; **Event:** samplingProtocol: Malaise trap; eventDate: 2007-02-26; **Record Level:** institutionCode: ENV
- **Type status:** Other material. **Occurrence:** individualCount: 1; sex: female; **Location:** country: Spain; stateProvince: Castellón; locality: Pobla de Benifassà, Natural Park of Tinença de Benifassà; verbatimElevation: 662 m; verbatimLatitude: 40°39'22''N; verbatimLongitude: 000°9'25''W; **Identification:** identifiedBy: F. J. Peris-Felipo; **Event:** samplingProtocol: Malaise trap; eventDate: 2007-04-23; **Record Level:** institutionCode: ENV
- **Type status:** Other material. **Occurrence:** individualCount: 1; sex: female; **Location:** country: Spain; stateProvince: Castellón; locality: Pobla de Benifassà, Natural Park of Tinença de Benifassà; verbatimElevation: 662 m; verbatimLatitude: 40°39'22''N; verbatimLongitude: 000°9'25''W; **Identification:** identifiedBy: F. J. Peris-Felipo; **Event:** samplingProtocol: Malaise trap; eventDate: 2007-05-14; **Record Level:** institutionCode: ENV
- **Type status:** Other material. **Occurrence:** individualCount: 1; sex: female; **Location:** country: Spain; stateProvince: Castellón; locality: Pobla de Benifassà, Natural Park of Tinença de Benifassà; verbatimElevation: 662 m; verbatimLatitude: 40°39'22''N; verbatimLongitude: 000°9'25''W; **Identification:** identifiedBy: F. J. Peris-Felipo; **Event:** samplingProtocol: Malaise trap; eventDate: 2007-05-28; **Record Level:** institutionCode: ENV
- **Type status:** Other material. **Occurrence:** individualCount: 1; sex: female; **Location:** country: Spain; stateProvince: Castellón; locality: Pobla de Benifassà, Natural Park of Tinença de Benifassà; verbatimElevation: 662 m; verbatimLatitude: 40°39'22''N; verbatimLongitude: 000°9'25''W; **Identification:** identifiedBy: F. J. Peris-Felipo; **Event:** samplingProtocol: Malaise trap; eventDate: 2007-06-18; **Record Level:** institutionCode: ENV
- **Type status:** Other material. **Occurrence:** individualCount: 1; sex: male; **Location:** country: Spain; stateProvince: Alicante; locality: Torrevieja, Natural Park of Lagunas de La Mata-Torrevieja; verbatimElevation: 6 m; verbatimLatitude: 38°01'49''N; verbatimLongitude: 000°42'00''W; **Identification:** identifiedBy: F. J. Peris-Felipo; **Event:** samplingProtocol: Malaise trap; eventDate: 2007-05-15; **Record Level:** institutionCode: ENV

#### Distribution

Spain.

### Aspilota valenciensis

Fischer, 1996

#### Materials

- **Type status:** Other material. **Occurrence:** individualCount: 1; sex: female; **Location:** country: Spain; stateProvince: Alicante; locality: Alcoi, Natural Park of Carrascal de La Font Roja; verbatimElevation: 1072 m; verbatimLatitude: 38°38'51''N; verbatimLongitude: 000°32'46''W; **Identification:** identifiedBy: F. J. Peris-Felipo; **Event:** samplingProtocol: Malaise trap; eventDate: 2007-05-21; **Record Level:** institutionCode: ENV
- **Type status:** Other material. **Occurrence:** individualCount: 1; sex: female; **Location:** country: Spain; stateProvince: Castellón; locality: Pobla de Benifassà, Natural Park of Tinença de Benifassà; verbatimElevation: 662 m; verbatimLatitude: 40°39'22''N; verbatimLongitude: 000°9'25''W; **Identification:** identifiedBy: F. J. Peris-Felipo; **Event:** samplingProtocol: Malaise trap; eventDate: 2004-05-18; **Record Level:** institutionCode: ENV
- **Type status:** Other material. **Occurrence:** individualCount: 4; sex: females; **Location:** country: Spain; stateProvince: Castellón; locality: Pobla de Benifassà, Natural Park of Tinença de Benifassà; verbatimElevation: 662 m; verbatimLatitude: 40°39'22''N; verbatimLongitude: 000°9'25''W; **Identification:** identifiedBy: F. J. Peris-Felipo; **Event:** samplingProtocol: Malaise trap; eventDate: 2004-06-01; **Record Level:** institutionCode: ENV
- **Type status:** Other material. **Occurrence:** individualCount: 1; sex: female; **Location:** country: Spain; stateProvince: Castellón; locality: Pobla de Benifassà, Natural Park of Tinença de Benifassà; verbatimElevation: 662 m; verbatimLatitude: 40°39'22''N; verbatimLongitude: 000°9'25''W; **Identification:** identifiedBy: F. J. Peris-Felipo; **Event:** samplingProtocol: Malaise trap; eventDate: 2004-10-07; **Record Level:** institutionCode: ENV
- **Type status:** Other material. **Occurrence:** individualCount: 1; sex: female; **Location:** country: Spain; stateProvince: Castellón; locality: Pobla de Benifassà, Natural Park of Tinença de Benifassà; verbatimElevation: 662 m; verbatimLatitude: 40°39'22''N; verbatimLongitude: 000°9'25''W; **Identification:** identifiedBy: F. J. Peris-Felipo; **Event:** samplingProtocol: Malaise trap; eventDate: 2005-01-18; **Record Level:** institutionCode: ENV
- **Type status:** Other material. **Occurrence:** individualCount: 1; sex: female; **Location:** country: Spain; stateProvince: Castellón; locality: Pobla de Benifassà, Natural Park of Tinença de Benifassà; verbatimElevation: 662 m; verbatimLatitude: 40°39'22''N; verbatimLongitude: 000°9'25''W; **Identification:** identifiedBy: F. J. Peris-Felipo; **Event:** samplingProtocol: Malaise trap; eventDate: 2005-05-04; **Record Level:** institutionCode: ENV
- **Type status:** Other material. **Occurrence:** individualCount: 2; sex: females; **Location:** country: Spain; stateProvince: Castellón; locality: Pobla de Benifassà, Natural Park of Tinença de Benifassà; verbatimElevation: 662 m; verbatimLatitude: 40°39'22''N; verbatimLongitude: 000°9'25''W; **Identification:** identifiedBy: F. J. Peris-Felipo; **Event:** samplingProtocol: Malaise trap; eventDate: 2005-05-31; **Record Level:** institutionCode: ENV
- **Type status:** Other material. **Occurrence:** individualCount: 1; sex: female; **Location:** country: Spain; stateProvince: Castellón; locality: Pobla de Benifassà, Natural Park of Tinença de Benifassà; verbatimElevation: 662 m; verbatimLatitude: 40°39'22''N; verbatimLongitude: 000°9'25''W; **Identification:** identifiedBy: F. J. Peris-Felipo; **Event:** samplingProtocol: Malaise trap; eventDate: 2005-06-13; **Record Level:** institutionCode: ENV
- **Type status:** Other material. **Occurrence:** individualCount: 1; sex: female; **Location:** country: Spain; stateProvince: Castellón; locality: Pobla de Benifassà, Natural Park of Tinença de Benifassà; verbatimElevation: 662 m; verbatimLatitude: 40°39'22''N; verbatimLongitude: 000°9'25''W; **Identification:** identifiedBy: F. J. Peris-Felipo; **Event:** samplingProtocol: Malaise trap; eventDate: 2005-06-19; **Record Level:** institutionCode: ENV
- **Type status:** Other material. **Occurrence:** individualCount: 1; sex: female; **Location:** country: Spain; stateProvince: Castellón; locality: Pobla de Benifassà, Natural Park of Tinença de Benifassà; verbatimElevation: 662 m; verbatimLatitude: 40°39'22''N; verbatimLongitude: 000°9'25''W; **Identification:** identifiedBy: F. J. Peris-Felipo; **Event:** samplingProtocol: Malaise trap; eventDate: 2005-06-27; **Record Level:** institutionCode: ENV
- **Type status:** Other material. **Occurrence:** individualCount: 1; sex: female; **Location:** country: Spain; stateProvince: Castellón; locality: Pobla de Benifassà, Natural Park of Tinença de Benifassà; verbatimElevation: 662 m; verbatimLatitude: 40°39'22''N; verbatimLongitude: 000°9'25''W; **Identification:** identifiedBy: F. J. Peris-Felipo; **Event:** samplingProtocol: Malaise trap; eventDate: 2006-04-17; **Record Level:** institutionCode: ENV
- **Type status:** Other material. **Occurrence:** individualCount: 1; sex: female; **Location:** country: Spain; stateProvince: Castellón; locality: Pobla de Benifassà, Natural Park of Tinença de Benifassà; verbatimElevation: 662 m; verbatimLatitude: 40°39'22''N; verbatimLongitude: 000°9'25''W; **Identification:** identifiedBy: F. J. Peris-Felipo; **Event:** samplingProtocol: Malaise trap; eventDate: 2006-04-24; **Record Level:** institutionCode: ENV
- **Type status:** Other material. **Occurrence:** individualCount: 1; sex: female; **Location:** country: Spain; stateProvince: Castellón; locality: Pobla de Benifassà, Natural Park of Tinença de Benifassà; verbatimElevation: 662 m; verbatimLatitude: 40°39'22''N; verbatimLongitude: 000°9'25''W; **Identification:** identifiedBy: F. J. Peris-Felipo; **Event:** samplingProtocol: Malaise trap; eventDate: 2006-05-01; **Record Level:** institutionCode: ENV
- **Type status:** Other material. **Occurrence:** individualCount: 1; sex: female; **Location:** country: Spain; stateProvince: Castellón; locality: Pobla de Benifassà, Natural Park of Tinença de Benifassà; verbatimElevation: 662 m; verbatimLatitude: 40°39'22''N; verbatimLongitude: 000°9'25''W; **Identification:** identifiedBy: F. J. Peris-Felipo; **Event:** samplingProtocol: Malaise trap; eventDate: 2006-05-15; **Record Level:** institutionCode: ENV
- **Type status:** Other material. **Occurrence:** individualCount: 1; sex: female; **Location:** country: Spain; stateProvince: Castellón; locality: Pobla de Benifassà, Natural Park of Tinença de Benifassà; verbatimElevation: 662 m; verbatimLatitude: 40°39'22''N; verbatimLongitude: 000°9'25''W; **Identification:** identifiedBy: F. J. Peris-Felipo; **Event:** samplingProtocol: Malaise trap; eventDate: 2006-05-29; **Record Level:** institutionCode: ENV
- **Type status:** Other material. **Occurrence:** individualCount: 1; sex: female; **Location:** country: Spain; stateProvince: Castellón; locality: Pobla de Benifassà, Natural Park of Tinença de Benifassà; verbatimElevation: 662 m; verbatimLatitude: 40°39'22''N; verbatimLongitude: 000°9'25''W; **Identification:** identifiedBy: F. J. Peris-Felipo; **Event:** samplingProtocol: Malaise trap; eventDate: 2006-06-06; **Record Level:** institutionCode: ENV
- **Type status:** Other material. **Occurrence:** individualCount: 1; sex: female; **Location:** country: Spain; stateProvince: Castellón; locality: Pobla de Benifassà, Natural Park of Tinença de Benifassà; verbatimElevation: 662 m; verbatimLatitude: 40°39'22''N; verbatimLongitude: 000°9'25''W; **Identification:** identifiedBy: F. J. Peris-Felipo; **Event:** samplingProtocol: Malaise trap; eventDate: 2006-08-20; **Record Level:** institutionCode: ENV
- **Type status:** Other material. **Occurrence:** individualCount: 1; sex: female; **Location:** country: Spain; stateProvince: Castellón; locality: Pobla de Benifassà, Natural Park of Tinença de Benifassà; verbatimElevation: 662 m; verbatimLatitude: 40°39'22''N; verbatimLongitude: 000°9'25''W; **Identification:** identifiedBy: F. J. Peris-Felipo; **Event:** samplingProtocol: Malaise trap; eventDate: 2007-04-30; **Record Level:** institutionCode: ENV
- **Type status:** Other material. **Occurrence:** individualCount: 1; sex: female; **Location:** country: Spain; stateProvince: Castellón; locality: Pobla de Benifassà, Natural Park of Tinença de Benifassà; verbatimElevation: 662 m; verbatimLatitude: 40°39'22''N; verbatimLongitude: 000°9'25''W; **Identification:** identifiedBy: F. J. Peris-Felipo; **Event:** samplingProtocol: Malaise trap; eventDate: 2007-05-15; **Record Level:** institutionCode: ENV
- **Type status:** Other material. **Occurrence:** individualCount: 1; sex: female; **Location:** country: Spain; stateProvince: Alicante; locality: Torrevieja, Natural Park of Lagunas de La Mata-Torrevieja; verbatimElevation: 6 m; verbatimLatitude: 38°01'49''N; verbatimLongitude: 000°42'00''W; **Identification:** identifiedBy: F. J. Peris-Felipo; **Event:** samplingProtocol: Malaise trap; eventDate: 2005-03-08; **Record Level:** institutionCode: ENV
- **Type status:** Other material. **Occurrence:** individualCount: 1; sex: male; **Location:** country: Spain; stateProvince: Alicante; locality: Torrevieja, Natural Park of Lagunas de La Mata-Torrevieja; verbatimElevation: 6 m; verbatimLatitude: 38°01'49''N; verbatimLongitude: 000°42'00''W; **Identification:** identifiedBy: F. J. Peris-Felipo; **Event:** samplingProtocol: Malaise trap; eventDate: 2006-03-14; **Record Level:** institutionCode: ENV
- **Type status:** Other material. **Occurrence:** individualCount: 1; sex: female; **Location:** country: Spain; stateProvince: Alicante; locality: Torrevieja, Natural Park of Lagunas de La Mata-Torrevieja; verbatimElevation: 6 m; verbatimLatitude: 38°01'49''N; verbatimLongitude: 000°42'00''W; **Identification:** identifiedBy: F. J. Peris-Felipo; **Event:** samplingProtocol: Malaise trap; eventDate: 2006-10-09; **Record Level:** institutionCode: ENV
- **Type status:** Other material. **Occurrence:** individualCount: 1; sex: female; **Location:** country: Spain; stateProvince: Alicante; locality: Torrevieja, Natural Park of Lagunas de La Mata-Torrevieja; verbatimElevation: 6 m; verbatimLatitude: 38°01'49''N; verbatimLongitude: 000°42'00''W; **Identification:** identifiedBy: F. J. Peris-Felipo; **Event:** samplingProtocol: Malaise trap; eventDate: 2006-10-23; **Record Level:** institutionCode: ENV

#### Distribution

Hungary and Spain.

### Aspilota sp1

#### Materials

- **Type status:** Other material. **Occurrence:** individualCount: 1; sex: female; **Location:** country: Spain; stateProvince: Castellón; locality: Pobla de Benifassà, Natural Park of Tinença de Benifassà; verbatimElevation: 662 m; verbatimLatitude: 40°39'22''N; verbatimLongitude: 000°9'25''W; **Identification:** identifiedBy: F. J. Peris-Felipo; **Event:** samplingProtocol: Malaise trap; eventDate: 2006-06-12; **Record Level:** institutionCode: ENV
- **Type status:** Other material. **Occurrence:** individualCount: 1; sex: female; **Location:** country: Spain; stateProvince: Alicante; locality: Torrevieja, Natural Park of Lagunas de La Mata-Torrevieja; verbatimElevation: 6 m; verbatimLatitude: 38°01'49''N; verbatimLongitude: 000°42'00''W; **Identification:** identifiedBy: F. J. Peris-Felipo; **Event:** samplingProtocol: Malaise trap; eventDate: 2004-05-18; **Record Level:** institutionCode: ENV
- **Type status:** Other material. **Occurrence:** individualCount: 2; sex: females; **Location:** country: Spain; stateProvince: Alicante; locality: Torrevieja, Natural Park of Lagunas de La Mata-Torrevieja; verbatimElevation: 6 m; verbatimLatitude: 38°01'49''N; verbatimLongitude: 000°42'00''W; **Identification:** identifiedBy: F. J. Peris-Felipo; **Event:** samplingProtocol: Malaise trap; eventDate: 2004-06-01; **Record Level:** institutionCode: ENV
- **Type status:** Other material. **Occurrence:** individualCount: 1; sex: female; **Location:** country: Spain; stateProvince: Alicante; locality: Torrevieja, Natural Park of Lagunas de La Mata-Torrevieja; verbatimElevation: 6 m; verbatimLatitude: 38°01'49''N; verbatimLongitude: 000°42'00''W; **Identification:** identifiedBy: F. J. Peris-Felipo; **Event:** samplingProtocol: Malaise trap; eventDate: 2005-06-27; **Record Level:** institutionCode: ENV
- **Type status:** Other material. **Occurrence:** individualCount: 1; sex: female; **Location:** country: Spain; stateProvince: Alicante; locality: Torrevieja, Natural Park of Lagunas de La Mata-Torrevieja; verbatimElevation: 6 m; verbatimLatitude: 38°01'49''N; verbatimLongitude: 000°42'00''W; **Identification:** identifiedBy: F. J. Peris-Felipo; **Event:** samplingProtocol: Malaise trap; eventDate: 2006-05-15; **Record Level:** institutionCode: ENV
- **Type status:** Other material. **Occurrence:** individualCount: 1; sex: female; **Location:** country: Spain; stateProvince: Alicante; locality: Torrevieja, Natural Park of Lagunas de La Mata-Torrevieja; verbatimElevation: 6 m; verbatimLatitude: 38°01'49''N; verbatimLongitude: 000°42'00''W; **Identification:** identifiedBy: F. J. Peris-Felipo; **Event:** samplingProtocol: Malaise trap; eventDate: 2006-06-05; **Record Level:** institutionCode: ENV
- **Type status:** Other material. **Occurrence:** individualCount: 1; sex: female; **Location:** country: Spain; stateProvince: Alicante; locality: Torrevieja, Natural Park of Lagunas de La Mata-Torrevieja; verbatimElevation: 6 m; verbatimLatitude: 38°01'49''N; verbatimLongitude: 000°42'00''W; **Identification:** identifiedBy: F. J. Peris-Felipo; **Event:** samplingProtocol: Malaise trap; eventDate: 2006-06-15; **Record Level:** institutionCode: ENV
- **Type status:** Other material. **Occurrence:** individualCount: 1; sex: female; **Location:** country: Spain; stateProvince: Alicante; locality: Torrevieja, Natural Park of Lagunas de La Mata-Torrevieja; verbatimElevation: 6 m; verbatimLatitude: 38°01'49''N; verbatimLongitude: 000°42'00''W; **Identification:** identifiedBy: F. J. Peris-Felipo; **Event:** samplingProtocol: Malaise trap; eventDate: 2007-05-15; **Record Level:** institutionCode: ENV
- **Type status:** Other material. **Occurrence:** individualCount: 1; sex: female; **Location:** country: Spain; stateProvince: Alicante; locality: Torrevieja, Natural Park of Lagunas de La Mata-Torrevieja; verbatimElevation: 6 m; verbatimLatitude: 38°01'49''N; verbatimLongitude: 000°42'00''W; **Identification:** identifiedBy: F. J. Peris-Felipo; **Event:** samplingProtocol: Malaise trap; eventDate: 2007-05-28; **Record Level:** institutionCode: ENV

### Aspilota sp2

#### Materials

- **Type status:** Other material. **Occurrence:** individualCount: 1; sex: female; **Location:** country: Spain; stateProvince: Alicante; locality: Torrevieja, Natural Park of Lagunas de La Mata-Torrevieja; verbatimElevation: 6 m; verbatimLatitude: 38°01'49''N; verbatimLongitude: 000°42'00''W; **Identification:** identifiedBy: F. J. Peris-Felipo; **Event:** samplingProtocol: Malaise trap; eventDate: 2004-06-08; **Record Level:** institutionCode: ENV
- **Type status:** Other material. **Occurrence:** individualCount: 1; sex: female; **Location:** country: Spain; stateProvince: Alicante; locality: Torrevieja, Natural Park of Lagunas de La Mata-Torrevieja; verbatimElevation: 6 m; verbatimLatitude: 38°01'49''N; verbatimLongitude: 000°42'00''W; **Identification:** identifiedBy: F. J. Peris-Felipo; **Event:** samplingProtocol: Malaise trap; eventDate: 2006-04-04; **Record Level:** institutionCode: ENV

### Dinotrema achterbergi

Peris-Felipo, 2013

#### Materials

- **Type status:** Holotype. **Occurrence:** individualCount: 1; sex: female; **Location:** country: Spain; stateProvince: Castellón; locality: Pobla de Benifassà, Natural Park of Tinença de Benifassà; verbatimElevation: 662 m; verbatimLatitude: 40°39'22''N; verbatimLongitude: 000°9'25''W; **Identification:** identifiedBy: F. J. Peris-Felipo; **Event:** samplingProtocol: Malaise trap; eventDate: 2006-06-10; **Record Level:** institutionCode: ENV
- **Type status:** Paratype. **Occurrence:** individualCount: 1; sex: female; **Location:** country: Spain; stateProvince: Castellón; locality: Pobla de Benifassà, Natural Park of Tinença de Benifassà; verbatimElevation: 662 m; verbatimLatitude: 40°39'22''N; verbatimLongitude: 000°9'25''W; **Identification:** identifiedBy: F. J. Peris-Felipo; **Event:** samplingProtocol: Malaise trap; eventDate: 2004-06-03; **Record Level:** institutionCode: ENV
- **Type status:** Other material. **Occurrence:** individualCount: 1; sex: female; **Location:** country: Spain; stateProvince: Alicante; locality: Alcoi, Natural Park of Carrascal de La Font Roja; verbatimElevation: 1072 m; verbatimLatitude: 38°38'51''N; verbatimLongitude: 000°32'46''W; **Identification:** identifiedBy: F. J. Peris-Felipo; **Event:** samplingProtocol: Malaise trap; eventDate: 2005-10-17; **Record Level:** institutionCode: ENV
- **Type status:** Other material. **Occurrence:** individualCount: 1; sex: female; **Location:** country: Spain; stateProvince: Alicante; locality: Alcoi, Natural Park of Carrascal de La Font Roja; verbatimElevation: 1072 m; verbatimLatitude: 38°38'51''N; verbatimLongitude: 000°32'46''W; **Identification:** identifiedBy: F. J. Peris-Felipo; **Event:** samplingProtocol: Malaise trap; eventDate: 2005-11-27; **Record Level:** institutionCode: ENV
- **Type status:** Other material. **Occurrence:** individualCount: 1; sex: female; **Location:** country: Spain; stateProvince: Castellón; locality: Pobla de Benifassà, Natural Park of Tinença de Benifassà; verbatimElevation: 662 m; verbatimLatitude: 40°39'22''N; verbatimLongitude: 000°9'25''W; **Identification:** identifiedBy: F. J. Peris-Felipo; **Event:** samplingProtocol: Malaise trap; eventDate: 2006-06-05; **Record Level:** institutionCode: ENV
- **Type status:** Other material. **Occurrence:** individualCount: 1; sex: female; **Location:** country: Spain; stateProvince: Castellón; locality: Pobla de Benifassà, Natural Park of Tinença de Benifassà; verbatimElevation: 662 m; verbatimLatitude: 40°39'22''N; verbatimLongitude: 000°9'25''W; **Identification:** identifiedBy: F. J. Peris-Felipo; **Event:** samplingProtocol: Malaise trap; eventDate: 2006-06-19; **Record Level:** institutionCode: ENV
- **Type status:** Other material. **Occurrence:** individualCount: 1; sex: female; **Location:** country: Spain; stateProvince: Castellón; locality: Pobla de Benifassà, Natural Park of Tinença de Benifassà; verbatimElevation: 662 m; verbatimLatitude: 40°39'22''N; verbatimLongitude: 000°9'25''W; **Identification:** identifiedBy: F. J. Peris-Felipo; **Event:** samplingProtocol: Malaise trap; eventDate: 2006-10-30; **Record Level:** institutionCode: ENV
- **Type status:** Other material. **Occurrence:** individualCount: 3; sex: females; **Location:** country: Spain; stateProvince: Castellón; locality: Pobla de Benifassà, Natural Park of Tinença de Benifassà; verbatimElevation: 662 m; verbatimLatitude: 40°39'22''N; verbatimLongitude: 000°9'25''W; **Identification:** identifiedBy: F. J. Peris-Felipo; **Event:** samplingProtocol: Malaise trap; eventDate: 2006-11-13; **Record Level:** institutionCode: ENV
- **Type status:** Other material. **Occurrence:** individualCount: 2; sex: females; **Location:** country: Spain; stateProvince: Castellón; locality: Pobla de Benifassà, Natural Park of Tinença de Benifassà; verbatimElevation: 662 m; verbatimLatitude: 40°39'22''N; verbatimLongitude: 000°9'25''W; **Identification:** identifiedBy: F. J. Peris-Felipo; **Event:** samplingProtocol: Malaise trap; eventDate: 2006-12-04; **Record Level:** institutionCode: ENV
- **Type status:** Other material. **Occurrence:** individualCount: 1; sex: female; **Location:** country: Spain; stateProvince: Castellón; locality: Pobla de Benifassà, Natural Park of Tinença de Benifassà; verbatimElevation: 662 m; verbatimLatitude: 40°39'22''N; verbatimLongitude: 000°9'25''W; **Identification:** identifiedBy: F. J. Peris-Felipo; **Event:** samplingProtocol: Malaise trap; eventDate: 2007-02-26; **Record Level:** institutionCode: ENV
- **Type status:** Other material. **Occurrence:** individualCount: 1; sex: female; **Location:** country: Spain; stateProvince: Castellón; locality: Pobla de Benifassà, Natural Park of Tinença de Benifassà; verbatimElevation: 662 m; verbatimLatitude: 38°01'49''N; verbatimLongitude: 000°42'00''W; **Identification:** identifiedBy: F. J. Peris-Felipo; **Event:** samplingProtocol: Malaise trap; eventDate: 2004-11-30; **Record Level:** institutionCode: ENV
- **Type status:** Other material. **Occurrence:** individualCount: 1; sex: female; **Location:** country: Spain; stateProvince: Alicante; locality: Torrevieja, Natural Park of Lagunas de La Mata-Torrevieja; verbatimElevation: 6 m; verbatimLatitude: 38°01'49''N; verbatimLongitude: 000°42'00''W; **Identification:** identifiedBy: F. J. Peris-Felipo; **Event:** samplingProtocol: Malaise trap; eventDate: 2006-12-26; **Record Level:** institutionCode: ENV
- **Type status:** Other material. **Occurrence:** individualCount: 1; sex: female; **Location:** country: Spain; stateProvince: Alicante; locality: Torrevieja, Natural Park of Lagunas de La Mata-Torrevieja; verbatimElevation: 6 m; verbatimLatitude: 38°01'49''N; verbatimLongitude: 000°42'00''W; **Identification:** identifiedBy: F. J. Peris-Felipo; **Event:** samplingProtocol: Malaise trap; eventDate: 2007-10-09; **Record Level:** institutionCode: ENV

#### Distribution

Spain.

### Dinotrema amparoae

Peris-Felipo, 2013

#### Materials

- **Type status:** Holotype. **Occurrence:** individualCount: 1; sex: female; **Location:** country: Spain; stateProvince: Alicante; locality: Torrevieja, Natural Park of Lagunas de La Mata-Torrevieja; verbatimElevation: 6 m; verbatimLatitude: 38°01'49''N; verbatimLongitude: 000°42'00''W; **Identification:** identifiedBy: F. J. Peris-Felipo; **Event:** samplingProtocol: Malaise trap; eventDate: 2005-02-08; **Record Level:** institutionCode: ENV
- **Type status:** Paratype. **Occurrence:** individualCount: 1; sex: female; **Location:** country: Spain; stateProvince: Alicante; locality: Torrevieja, Natural Park of Lagunas de La Mata-Torrevieja; verbatimElevation: 6 m; verbatimLatitude: 38°01'49''N; verbatimLongitude: 000°42'00''W; **Identification:** identifiedBy: F. J. Peris-Felipo; **Event:** samplingProtocol: Malaise trap; eventDate: 2005-03-04; **Record Level:** institutionCode: ENV
- **Type status:** Paratype. **Occurrence:** individualCount: 1; sex: female; **Location:** country: Spain; stateProvince: Alicante; locality: Torrevieja, Natural Park of Lagunas de La Mata-Torrevieja; verbatimElevation: 6 m; verbatimLatitude: 38°01'49''N; verbatimLongitude: 000°42'00''W; **Identification:** identifiedBy: F. J. Peris-Felipo; **Event:** samplingProtocol: Malaise trap; eventDate: 2005-04-05; **Record Level:** institutionCode: ENV
- **Type status:** Paratype. **Occurrence:** individualCount: 1; sex: female; **Location:** country: Spain; stateProvince: Alicante; locality: Torrevieja, Natural Park of Lagunas de La Mata-Torrevieja; verbatimElevation: 6 m; verbatimLatitude: 38°01'49''N; verbatimLongitude: 000°42'00''W; **Identification:** identifiedBy: F. J. Peris-Felipo; **Event:** samplingProtocol: Malaise trap; eventDate: 2006-03-28; **Record Level:** institutionCode: ZISP
- **Type status:** Paratype. **Occurrence:** individualCount: 1; sex: male; **Location:** country: Spain; stateProvince: Alicante; locality: Torrevieja, Natural Park of Lagunas de La Mata-Torrevieja; verbatimElevation: 6 m; verbatimLatitude: 38°01'49''N; verbatimLongitude: 000°42'00''W; **Identification:** identifiedBy: F. J. Peris-Felipo; **Event:** samplingProtocol: Malaise trap; eventDate: 2004-11-30; **Record Level:** institutionCode: ENV

#### Distribution

Spain.

### Dinotrema belokobylskiji

Peris-Felipo, 2013

#### Materials

- **Type status:** Holotype. **Occurrence:** individualCount: 1; sex: female; **Location:** country: Spain; stateProvince: Castellón; locality: Pobla de Benifassà, Natural Park of Tinença de Benifassà; verbatimElevation: 662 m; verbatimLatitude: 40°39'22''N; verbatimLongitude: 000°9'25''W; **Identification:** identifiedBy: F. J. Peris-Felipo; **Event:** samplingProtocol: Malaise trap; eventDate: 2006-10-21; **Record Level:** institutionCode: ENV
- **Type status:** Paratype. **Occurrence:** individualCount: 1; sex: female; **Location:** country: Spain; stateProvince: Castellón; locality: Pobla de Benifassà, Natural Park of Tinença de Benifassà; verbatimElevation: 662 m; verbatimLatitude: 40°39'22''N; verbatimLongitude: 000°9'25''W; **Identification:** identifiedBy: F. J. Peris-Felipo; **Event:** samplingProtocol: Malaise trap; eventDate: 2006-10-30; **Record Level:** institutionCode: ENV

#### Distribution

Spain.

### Dinotrema benifassaense

Peris-Felipo, 2013

#### Materials

- **Type status:** Holotype. **Occurrence:** individualCount: 1; sex: female; **Location:** country: Spain; stateProvince: Castellón; locality: Pobla de Benifassà, Natural Park of Tinença de Benifassà; verbatimElevation: 662 m; verbatimLatitude: 40°39'22''N; verbatimLongitude: 000°9'25''W; **Identification:** identifiedBy: F. J. Peris-Felipo; **Event:** samplingProtocol: Malaise trap; eventDate: 2007-06-11; **Record Level:** institutionCode: ENV
- **Type status:** Paratype. **Occurrence:** individualCount: 1; sex: female; **Location:** country: Spain; stateProvince: Castellón; locality: Pobla de Benifassà, Natural Park of Tinença de Benifassà; verbatimElevation: 662 m; verbatimLatitude: 40°39'22''N; verbatimLongitude: 000°9'25''W; **Identification:** identifiedBy: F. J. Peris-Felipo; **Event:** samplingProtocol: Malaise trap; eventDate: 2007-05-14; **Record Level:** institutionCode: ENV

#### Distribution

Spain.

### Dinotrema broadi

Peris-Felipo, 2013

#### Materials

- **Type status:** Holotype. **Occurrence:** individualCount: 1; sex: female; **Location:** country: Spain; stateProvince: Castellón; locality: Pobla de Benifassà, Natural Park of Tinença de Benifassà; verbatimElevation: 662 m; verbatimLatitude: 40°39'22''N; verbatimLongitude: 000°9'25''W; **Identification:** identifiedBy: F. J. Peris-Felipo; **Event:** samplingProtocol: Malaise trap; eventDate: 2006-10-30; **Record Level:** institutionCode: ENV
- **Type status:** Paratype. **Occurrence:** individualCount: 1; sex: female; **Location:** country: Spain; stateProvince: Castellón; locality: Pobla de Benifassà, Natural Park of Tinença de Benifassà; verbatimElevation: 662 m; verbatimLatitude: 40°39'22''N; verbatimLongitude: 000°9'25''W; **Identification:** identifiedBy: F. J. Peris-Felipo; **Event:** samplingProtocol: Malaise trap; eventDate: 2006-07-03; **Record Level:** institutionCode: ENV
- **Type status:** Paratype. **Occurrence:** individualCount: 1; sex: female; **Location:** country: Spain; stateProvince: Castellón; locality: Pobla de Benifassà, Natural Park of Tinença de Benifassà; verbatimElevation: 662 m; verbatimLatitude: 40°39'22''N; verbatimLongitude: 000°9'25''W; **Identification:** identifiedBy: F. J. Peris-Felipo; **Event:** samplingProtocol: Malaise trap; eventDate: 2006-11-06; **Record Level:** institutionCode: ZISP
- **Type status:** Paratype. **Occurrence:** individualCount: 1; sex: female; **Location:** country: Spain; stateProvince: Castellón; locality: Pobla de Benifassà, Natural Park of Tinença de Benifassà; verbatimElevation: 662 m; verbatimLatitude: 40°39'22''N; verbatimLongitude: 000°9'25''W; **Identification:** identifiedBy: F. J. Peris-Felipo; **Event:** samplingProtocol: Malaise trap; eventDate: 2007-02-26; **Record Level:** institutionCode: ENV
- **Type status:** Paratype. **Occurrence:** individualCount: 1; sex: male; **Location:** country: Spain; stateProvince: Castellón; locality: Pobla de Benifassà, Natural Park of Tinença de Benifassà; verbatimElevation: 662 m; verbatimLatitude: 40°39'22''N; verbatimLongitude: 000°9'25''W; **Identification:** identifiedBy: F. J. Peris-Felipo; **Event:** samplingProtocol: Malaise trap; eventDate: 2006-05-01; **Record Level:** institutionCode: ZISP
- **Type status:** Paratype. **Occurrence:** individualCount: 1; sex: male; **Location:** country: Spain; stateProvince: Castellón; locality: Pobla de Benifassà, Natural Park of Tinença de Benifassà; verbatimElevation: 662 m; verbatimLatitude: 40°39'22''N; verbatimLongitude: 000°9'25''W; **Identification:** identifiedBy: F. J. Peris-Felipo; **Event:** samplingProtocol: Malaise trap; eventDate: 2006-05-29; **Record Level:** institutionCode: ENV
- **Type status:** Other material. **Occurrence:** individualCount: 1; sex: male; **Location:** country: Spain; stateProvince: Castellón; locality: Pobla de Benifassà, Natural Park of Tinença de Benifassà; verbatimElevation: 662 m; verbatimLatitude: 40°39'22''N; verbatimLongitude: 000°9'25''W; **Identification:** identifiedBy: F. J. Peris-Felipo; **Event:** samplingProtocol: Malaise trap; eventDate: 2004-06-02; **Record Level:** institutionCode: ENV
- **Type status:** Other material. **Occurrence:** individualCount: 2; sex: males; **Location:** country: Spain; stateProvince: Castellón; locality: Pobla de Benifassà, Natural Park of Tinença de Benifassà; verbatimElevation: 662 m; verbatimLatitude: 40°39'22''N; verbatimLongitude: 000°9'25''W; **Identification:** identifiedBy: F. J. Peris-Felipo; **Event:** samplingProtocol: Malaise trap; eventDate: 2006-10-30; **Record Level:** institutionCode: ENV
- **Type status:** Other material. **Occurrence:** individualCount: 1; sex: male; **Location:** country: Spain; stateProvince: Castellón; locality: Pobla de Benifassà, Natural Park of Tinença de Benifassà; verbatimElevation: 662 m; verbatimLatitude: 40°39'22''N; verbatimLongitude: 000°9'25''W; **Identification:** identifiedBy: F. J. Peris-Felipo; **Event:** samplingProtocol: Malaise trap; eventDate: 2007-01-08; **Record Level:** institutionCode: ENV
- **Type status:** Other material. **Occurrence:** individualCount: 1; sex: female; **Location:** country: Spain; stateProvince: Castellón; locality: Pobla de Benifassà, Natural Park of Tinença de Benifassà; verbatimElevation: 662 m; verbatimLatitude: 40°39'22''N; verbatimLongitude: 000°9'25''W; **Identification:** identifiedBy: F. J. Peris-Felipo; **Event:** samplingProtocol: Malaise trap; eventDate: 2005-11-27; **Record Level:** institutionCode: ENV
- **Type status:** Other material. **Occurrence:** individualCount: 1; sex: male; **Location:** country: Spain; stateProvince: Alicante; locality: Alcoi, Natural Park of Carrascal de La Font Roja; verbatimElevation: 1072 m; verbatimLatitude: 38°38'51''N; verbatimLongitude: 000°32'46''W; **Identification:** identifiedBy: F. J. Peris-Felipo; **Event:** samplingProtocol: Malaise trap; eventDate: 2005-05-16; **Record Level:** institutionCode: ENV

#### Distribution

Spain.

### Dinotrema castaneithorax

(Fischer, 1973)

#### Materials

- **Type status:** Other material. **Occurrence:** individualCount: 1; sex: female; **Location:** country: Spain; stateProvince: Castellón; locality: Pobla de Benifassà, Natural Park of Tinença de Benifassà; verbatimElevation: 662 m; verbatimLatitude: 40°39'22''N; verbatimLongitude: 000°9'25''W; **Identification:** identifiedBy: F. J. Peris-Felipo; **Event:** samplingProtocol: Malaise trap; eventDate: 2006-05-01; **Record Level:** institutionCode: ENV
- **Type status:** Other material. **Occurrence:** individualCount: 2; sex: females; **Location:** country: Spain; stateProvince: Castellón; locality: Pobla de Benifassà, Natural Park of Tinença de Benifassà; verbatimElevation: 662 m; verbatimLatitude: 40°39'22''N; verbatimLongitude: 000°9'25''W; **Identification:** identifiedBy: F. J. Peris-Felipo; **Event:** samplingProtocol: Malaise trap; eventDate: 2006-05-08; **Record Level:** institutionCode: ENV
- **Type status:** Other material. **Occurrence:** individualCount: 1; sex: female; **Location:** country: Spain; stateProvince: Castellón; locality: Pobla de Benifassà, Natural Park of Tinença de Benifassà; verbatimElevation: 662 m; verbatimLatitude: 40°39'22''N; verbatimLongitude: 000°9'25''W; **Identification:** identifiedBy: F. J. Peris-Felipo; **Event:** samplingProtocol: Malaise trap; eventDate: 2006-05-22; **Record Level:** institutionCode: ENV
- **Type status:** Other material. **Occurrence:** individualCount: 1; sex: female; **Location:** country: Spain; stateProvince: Castellón; locality: Pobla de Benifassà, Natural Park of Tinença de Benifassà; verbatimElevation: 662 m; verbatimLatitude: 40°39'22''N; verbatimLongitude: 000°9'25''W; **Identification:** identifiedBy: F. J. Peris-Felipo; **Event:** samplingProtocol: Malaise trap; eventDate: 2006-05-29; **Record Level:** institutionCode: ENV
- **Type status:** Other material. **Occurrence:** individualCount: 2; sex: females; **Location:** country: Spain; stateProvince: Castellón; locality: Pobla de Benifassà, Natural Park of Tinença de Benifassà; verbatimElevation: 662 m; verbatimLatitude: 40°39'22''N; verbatimLongitude: 000°9'25''W; **Identification:** identifiedBy: F. J. Peris-Felipo; **Event:** samplingProtocol: Malaise trap; eventDate: 2006-06-12; **Record Level:** institutionCode: ENV
- **Type status:** Other material. **Occurrence:** individualCount: 1; sex: female; **Location:** country: Spain; stateProvince: Castellón; locality: Pobla de Benifassà, Natural Park of Tinença de Benifassà; verbatimElevation: 662 m; verbatimLatitude: 40°39'22''N; verbatimLongitude: 000°9'25''W; **Identification:** identifiedBy: F. J. Peris-Felipo; **Event:** samplingProtocol: Malaise trap; eventDate: 2006-08-01; **Record Level:** institutionCode: ENV
- **Type status:** Other material. **Occurrence:** individualCount: 1; sex: female; **Location:** country: Spain; stateProvince: Castellón; locality: Pobla de Benifassà, Natural Park of Tinença de Benifassà; verbatimElevation: 662 m; verbatimLatitude: 40°39'22''N; verbatimLongitude: 000°9'25''W; **Identification:** identifiedBy: F. J. Peris-Felipo; **Event:** samplingProtocol: Malaise trap; eventDate: 2007-02-12; **Record Level:** institutionCode: ENV
- **Type status:** Other material. **Occurrence:** individualCount: 1; sex: female; **Location:** country: Spain; stateProvince: Castellón; locality: Pobla de Benifassà, Natural Park of Tinença de Benifassà; verbatimElevation: 662 m; verbatimLatitude: 40°39'22''N; verbatimLongitude: 000°9'25''W; **Identification:** identifiedBy: F. J. Peris-Felipo; **Event:** samplingProtocol: Malaise trap; eventDate: 2007-04-30; **Record Level:** institutionCode: ENV
- **Type status:** Other material. **Occurrence:** individualCount: 2; sex: females; **Location:** country: Spain; stateProvince: Castellón; locality: Pobla de Benifassà, Natural Park of Tinença de Benifassà; verbatimElevation: 662 m; verbatimLatitude: 40°39'22''N; verbatimLongitude: 000°9'25''W; **Identification:** identifiedBy: F. J. Peris-Felipo; **Event:** samplingProtocol: Malaise trap; eventDate: 2007-05-07; **Record Level:** institutionCode: ENV
- **Type status:** Other material. **Occurrence:** individualCount: 1; sex: female; **Location:** country: Spain; stateProvince: Castellón; locality: Pobla de Benifassà, Natural Park of Tinença de Benifassà; verbatimElevation: 662 m; verbatimLatitude: 40°39'22''N; verbatimLongitude: 000°9'25''W; **Identification:** identifiedBy: F. J. Peris-Felipo; **Event:** samplingProtocol: Malaise trap; eventDate: 2007-05-28; **Record Level:** institutionCode: ENV
- **Type status:** Other material. **Occurrence:** individualCount: 1; sex: female; **Location:** country: Spain; stateProvince: Castellón; locality: Pobla de Benifassà, Natural Park of Tinença de Benifassà; verbatimElevation: 662 m; verbatimLatitude: 40°39'22''N; verbatimLongitude: 000°9'25''W; **Identification:** identifiedBy: F. J. Peris-Felipo; **Event:** samplingProtocol: Malaise trap; eventDate: 2007-06-04; **Record Level:** institutionCode: ENV
- **Type status:** Other material. **Occurrence:** individualCount: 4; sex: females; **Location:** country: Spain; stateProvince: Castellón; locality: Pobla de Benifassà, Natural Park of Tinença de Benifassà; verbatimElevation: 662 m; verbatimLatitude: 40°39'22''N; verbatimLongitude: 000°9'25''W; **Identification:** identifiedBy: F. J. Peris-Felipo; **Event:** samplingProtocol: Malaise trap; eventDate: 2007-06-12; **Record Level:** institutionCode: ENV
- **Type status:** Other material. **Occurrence:** individualCount: 1; sex: female; **Location:** country: Spain; stateProvince: Castellón; locality: Pobla de Benifassà, Natural Park of Tinença de Benifassà; verbatimElevation: 662 m; verbatimLatitude: 40°39'22''N; verbatimLongitude: 000°9'25''W; **Identification:** identifiedBy: F. J. Peris-Felipo; **Event:** samplingProtocol: Malaise trap; eventDate: 2007-06-25; **Record Level:** institutionCode: ENV
- **Type status:** Other material. **Occurrence:** individualCount: 1; sex: female; **Location:** country: Spain; stateProvince: Castellón; locality: Pobla de Benifassà, Natural Park of Tinença de Benifassà; verbatimElevation: 662 m; verbatimLatitude: 40°39'22''N; verbatimLongitude: 000°9'25''W; **Identification:** identifiedBy: F. J. Peris-Felipo; **Event:** samplingProtocol: Malaise trap; eventDate: 2007-08-13; **Record Level:** institutionCode: ENV
- **Type status:** Other material. **Occurrence:** individualCount: 2; sex: females; **Location:** country: Spain; stateProvince: Castellón; locality: Pobla de Benifassà, Natural Park of Tinença de Benifassà; verbatimElevation: 662 m; verbatimLatitude: 40°39'22''N; verbatimLongitude: 000°9'25''W; **Identification:** identifiedBy: F. J. Peris-Felipo; **Event:** samplingProtocol: Malaise trap; eventDate: 2007-09-10; **Record Level:** institutionCode: ENV
- **Type status:** Other material. **Occurrence:** individualCount: 1; sex: female; **Location:** country: Spain; stateProvince: Castellón; locality: Pobla de Benifassà, Natural Park of Tinença de Benifassà; verbatimElevation: 662 m; verbatimLatitude: 40°39'22''N; verbatimLongitude: 000°9'25''W; **Identification:** identifiedBy: F. J. Peris-Felipo; **Event:** samplingProtocol: Malaise trap; eventDate: 2007-09-17; **Record Level:** institutionCode: ENV

#### Distribution

Austria, Hungary, Korea, Romania and Spain.

### Dinotrema costulatum

(Thomson, 1895)

#### Materials

- **Type status:** Other material. **Occurrence:** individualCount: 1; sex: female; **Location:** country: Spain; stateProvince: Alicante; locality: Alcoi, Natural Park of Carrascal de La Font Roja; verbatimElevation: 1072 m; verbatimLatitude: 38°38'51''N; verbatimLongitude: 000°32'46''W; **Identification:** identifiedBy: F. J. Peris-Felipo; **Event:** samplingProtocol: Malaise trap; eventDate: 2005-05-16; **Record Level:** institutionCode: ENV
- **Type status:** Other material. **Occurrence:** individualCount: 1; sex: female; **Location:** country: Spain; stateProvince: Alicante; locality: Alcoi, Natural Park of Carrascal de La Font Roja; verbatimElevation: 1072 m; verbatimLatitude: 38°38'51''N; verbatimLongitude: 000°32'46''W; **Identification:** identifiedBy: F. J. Peris-Felipo; **Event:** samplingProtocol: Malaise trap; eventDate: 2006-06-19; **Record Level:** institutionCode: ENV
- **Type status:** Other material. **Occurrence:** individualCount: 1; sex: female; **Location:** country: Spain; stateProvince: Alicante; locality: Alcoi, Natural Park of Carrascal de La Font Roja; verbatimElevation: 1072 m; verbatimLatitude: 38°38'51''N; verbatimLongitude: 000°32'46''W; **Identification:** identifiedBy: F. J. Peris-Felipo; **Event:** samplingProtocol: Malaise trap; eventDate: 2007-03-05; **Record Level:** institutionCode: ENV
- **Type status:** Other material. **Occurrence:** individualCount: 1; sex: female; **Location:** country: Spain; stateProvince: Alicante; locality: Alcoi, Natural Park of Carrascal de La Font Roja; verbatimElevation: 1072 m; verbatimLatitude: 38°38'51''N; verbatimLongitude: 000°32'46''W; **Identification:** identifiedBy: F. J. Peris-Felipo; **Event:** samplingProtocol: Malaise trap; eventDate: 2007-05-14; **Record Level:** institutionCode: ENV
- **Type status:** Other material. **Occurrence:** individualCount: 1; sex: female; **Location:** country: Spain; stateProvince: Alicante; locality: Alcoi, Natural Park of Carrascal de La Font Roja; verbatimElevation: 1072 m; verbatimLatitude: 38°38'51''N; verbatimLongitude: 000°32'46''W; **Identification:** identifiedBy: F. J. Peris-Felipo; **Event:** samplingProtocol: Malaise trap; eventDate: 2007-05-28; **Record Level:** institutionCode: ENV
- **Type status:** Other material. **Occurrence:** individualCount: 1; sex: female; **Location:** country: Spain; stateProvince: Castellón; locality: Pobla de Benifassà, Natural Park of Tinença de Benifassà; verbatimElevation: 662 m; verbatimLatitude: 40°39'22''N; verbatimLongitude: 000°9'25''W; **Identification:** identifiedBy: F. J. Peris-Felipo; **Event:** samplingProtocol: Malaise trap; eventDate: 2004-07-29; **Record Level:** institutionCode: ENV
- **Type status:** Other material. **Occurrence:** individualCount: 1; sex: female; **Location:** country: Spain; stateProvince: Castellón; locality: Pobla de Benifassà, Natural Park of Tinença de Benifassà; verbatimElevation: 662 m; verbatimLatitude: 40°39'22''N; verbatimLongitude: 000°9'25''W; **Identification:** identifiedBy: F. J. Peris-Felipo; **Event:** samplingProtocol: Malaise trap; eventDate: 2004-10-28; **Record Level:** institutionCode: ENV
- **Type status:** Other material. **Occurrence:** individualCount: 1; sex: female; **Location:** country: Spain; stateProvince: Castellón; locality: Pobla de Benifassà, Natural Park of Tinença de Benifassà; verbatimElevation: 662 m; verbatimLatitude: 40°39'22''N; verbatimLongitude: 000°9'25''W; **Identification:** identifiedBy: F. J. Peris-Felipo; **Event:** samplingProtocol: Malaise trap; eventDate: 2004-11-04; **Record Level:** institutionCode: ENV
- **Type status:** Other material. **Occurrence:** individualCount: 1; sex: female; **Location:** country: Spain; stateProvince: Castellón; locality: Pobla de Benifassà, Natural Park of Tinença de Benifassà; verbatimElevation: 662 m; verbatimLatitude: 40°39'22''N; verbatimLongitude: 000°9'25''W; **Identification:** identifiedBy: F. J. Peris-Felipo; **Event:** samplingProtocol: Malaise trap; eventDate: 2004-11-18; **Record Level:** institutionCode: ENV
- **Type status:** Other material. **Occurrence:** individualCount: 1; sex: female; **Location:** country: Spain; stateProvince: Castellón; locality: Pobla de Benifassà, Natural Park of Tinença de Benifassà; verbatimElevation: 662 m; verbatimLatitude: 40°39'22''N; verbatimLongitude: 000°9'25''W; **Identification:** identifiedBy: F. J. Peris-Felipo; **Event:** samplingProtocol: Malaise trap; eventDate: 2005-06-27; **Record Level:** institutionCode: ENV
- **Type status:** Other material. **Occurrence:** individualCount: 2; sex: females; **Location:** country: Spain; stateProvince: Castellón; locality: Pobla de Benifassà, Natural Park of Tinença de Benifassà; verbatimElevation: 662 m; verbatimLatitude: 40°39'22''N; verbatimLongitude: 000°9'25''W; **Identification:** identifiedBy: F. J. Peris-Felipo; **Event:** samplingProtocol: Malaise trap; eventDate: 2005-07-11; **Record Level:** institutionCode: ENV
- **Type status:** Other material. **Occurrence:** individualCount: 1; sex: female; **Location:** country: Spain; stateProvince: Castellón; locality: Pobla de Benifassà, Natural Park of Tinença de Benifassà; verbatimElevation: 662 m; verbatimLatitude: 40°39'22''N; verbatimLongitude: 000°9'25''W; **Identification:** identifiedBy: F. J. Peris-Felipo; **Event:** samplingProtocol: Malaise trap; eventDate: 2006-04-17; **Record Level:** institutionCode: ENV
- **Type status:** Other material. **Occurrence:** individualCount: 2; sex: females; **Location:** country: Spain; stateProvince: Castellón; locality: Pobla de Benifassà, Natural Park of Tinença de Benifassà; verbatimElevation: 662 m; verbatimLatitude: 40°39'22''N; verbatimLongitude: 000°9'25''W; **Identification:** identifiedBy: F. J. Peris-Felipo; **Event:** samplingProtocol: Malaise trap; eventDate: 2006-04-24; **Record Level:** institutionCode: ENV
- **Type status:** Other material. **Occurrence:** individualCount: 1; sex: female; **Location:** country: Spain; stateProvince: Castellón; locality: Pobla de Benifassà, Natural Park of Tinença de Benifassà; verbatimElevation: 662 m; verbatimLatitude: 40°39'22''N; verbatimLongitude: 000°9'25''W; **Identification:** identifiedBy: F. J. Peris-Felipo; **Event:** samplingProtocol: Malaise trap; eventDate: 2006-05-01; **Record Level:** institutionCode: ENV
- **Type status:** Other material. **Occurrence:** individualCount: 3; sex: females; **Location:** country: Spain; stateProvince: Castellón; locality: Pobla de Benifassà, Natural Park of Tinença de Benifassà; verbatimElevation: 662 m; verbatimLatitude: 40°39'22''N; verbatimLongitude: 000°9'25''W; **Identification:** identifiedBy: F. J. Peris-Felipo; **Event:** samplingProtocol: Malaise trap; eventDate: 2006-05-08; **Record Level:** institutionCode: ENV
- **Type status:** Other material. **Occurrence:** individualCount: 1; sex: female; **Location:** country: Spain; stateProvince: Castellón; locality: Pobla de Benifassà, Natural Park of Tinença de Benifassà; verbatimElevation: 662 m; verbatimLatitude: 40°39'22''N; verbatimLongitude: 000°9'25''W; **Identification:** identifiedBy: F. J. Peris-Felipo; **Event:** samplingProtocol: Malaise trap; eventDate: 2006-05-22; **Record Level:** institutionCode: ENV
- **Type status:** Other material. **Occurrence:** individualCount: 1; sex: female; **Location:** country: Spain; stateProvince: Castellón; locality: Pobla de Benifassà, Natural Park of Tinença de Benifassà; verbatimElevation: 662 m; verbatimLatitude: 40°39'22''N; verbatimLongitude: 000°9'25''W; **Identification:** identifiedBy: F. J. Peris-Felipo; **Event:** samplingProtocol: Malaise trap; eventDate: 2006-05-29; **Record Level:** institutionCode: ENV
- **Type status:** Other material. **Occurrence:** individualCount: 2; sex: females; **Location:** country: Spain; stateProvince: Castellón; locality: Pobla de Benifassà, Natural Park of Tinença de Benifassà; verbatimElevation: 662 m; verbatimLatitude: 40°39'22''N; verbatimLongitude: 000°9'25''W; **Identification:** identifiedBy: F. J. Peris-Felipo; **Event:** samplingProtocol: Malaise trap; eventDate: 2006-06-05; **Record Level:** institutionCode: ENV
- **Type status:** Other material. **Occurrence:** individualCount: 1; sex: female; **Location:** country: Spain; stateProvince: Castellón; locality: Pobla de Benifassà, Natural Park of Tinença de Benifassà; verbatimElevation: 662 m; verbatimLatitude: 40°39'22''N; verbatimLongitude: 000°9'25''W; **Identification:** identifiedBy: F. J. Peris-Felipo; **Event:** samplingProtocol: Malaise trap; eventDate: 2006-06-12; **Record Level:** institutionCode: ENV
- **Type status:** Other material. **Occurrence:** individualCount: 3; sex: females; **Location:** country: Spain; stateProvince: Castellón; locality: Pobla de Benifassà, Natural Park of Tinença de Benifassà; verbatimElevation: 662 m; verbatimLatitude: 40°39'22''N; verbatimLongitude: 000°9'25''W; **Identification:** identifiedBy: F. J. Peris-Felipo; **Event:** samplingProtocol: Malaise trap; eventDate: 2006-06-26; **Record Level:** institutionCode: ENV
- **Type status:** Other material. **Occurrence:** individualCount: 1; sex: female; **Location:** country: Spain; stateProvince: Castellón; locality: Pobla de Benifassà, Natural Park of Tinença de Benifassà; verbatimElevation: 662 m; verbatimLatitude: 40°39'22''N; verbatimLongitude: 000°9'25''W; **Identification:** identifiedBy: F. J. Peris-Felipo; **Event:** samplingProtocol: Malaise trap; eventDate: 2006-09-25; **Record Level:** institutionCode: ENV
- **Type status:** Other material. **Occurrence:** individualCount: 1; sex: female; **Location:** country: Spain; stateProvince: Castellón; locality: Pobla de Benifassà, Natural Park of Tinença de Benifassà; verbatimElevation: 662 m; verbatimLatitude: 40°39'22''N; verbatimLongitude: 000°9'25''W; **Identification:** identifiedBy: F. J. Peris-Felipo; **Event:** samplingProtocol: Malaise trap; eventDate: 2006-10-02; **Record Level:** institutionCode: ENV
- **Type status:** Other material. **Occurrence:** individualCount: 1; sex: female; **Location:** country: Spain; stateProvince: Castellón; locality: Pobla de Benifassà, Natural Park of Tinença de Benifassà; verbatimElevation: 662 m; verbatimLatitude: 40°39'22''N; verbatimLongitude: 000°9'25''W; **Identification:** identifiedBy: F. J. Peris-Felipo; **Event:** samplingProtocol: Malaise trap; eventDate: 2006-10-23; **Record Level:** institutionCode: ENV
- **Type status:** Other material. **Occurrence:** individualCount: 2; sex: females; **Location:** country: Spain; stateProvince: Castellón; locality: Pobla de Benifassà, Natural Park of Tinença de Benifassà; verbatimElevation: 662 m; verbatimLatitude: 40°39'22''N; verbatimLongitude: 000°9'25''W; **Identification:** identifiedBy: F. J. Peris-Felipo; **Event:** samplingProtocol: Malaise trap; eventDate: 2006-10-30; **Record Level:** institutionCode: ENV
- **Type status:** Other material. **Occurrence:** individualCount: 3; sex: females; **Location:** country: Spain; stateProvince: Castellón; locality: Pobla de Benifassà, Natural Park of Tinença de Benifassà; verbatimElevation: 662 m; verbatimLatitude: 40°39'22''N; verbatimLongitude: 000°9'25''W; **Identification:** identifiedBy: F. J. Peris-Felipo; **Event:** samplingProtocol: Malaise trap; eventDate: 2007-01-01; **Record Level:** institutionCode: ENV
- **Type status:** Other material. **Occurrence:** individualCount: 1; sex: female; **Location:** country: Spain; stateProvince: Castellón; locality: Pobla de Benifassà, Natural Park of Tinença de Benifassà; verbatimElevation: 662 m; verbatimLatitude: 40°39'22''N; verbatimLongitude: 000°9'25''W; **Identification:** identifiedBy: F. J. Peris-Felipo; **Event:** samplingProtocol: Malaise trap; eventDate: 2007-01-22; **Record Level:** institutionCode: ENV
- **Type status:** Other material. **Occurrence:** individualCount: 1; sex: female; **Location:** country: Spain; stateProvince: Castellón; locality: Pobla de Benifassà, Natural Park of Tinença de Benifassà; verbatimElevation: 662 m; verbatimLatitude: 40°39'22''N; verbatimLongitude: 000°9'25''W; **Identification:** identifiedBy: F. J. Peris-Felipo; **Event:** samplingProtocol: Malaise trap; eventDate: 2007-02-19; **Record Level:** institutionCode: ENV
- **Type status:** Other material. **Occurrence:** individualCount: 1; sex: female; **Location:** country: Spain; stateProvince: Castellón; locality: Pobla de Benifassà, Natural Park of Tinença de Benifassà; verbatimElevation: 662 m; verbatimLatitude: 40°39'22''N; verbatimLongitude: 000°9'25''W; **Identification:** identifiedBy: F. J. Peris-Felipo; **Event:** samplingProtocol: Malaise trap; eventDate: 2007-03-05; **Record Level:** institutionCode: ENV
- **Type status:** Other material. **Occurrence:** individualCount: 1; sex: female; **Location:** country: Spain; stateProvince: Castellón; locality: Pobla de Benifassà, Natural Park of Tinença de Benifassà; verbatimElevation: 662 m; verbatimLatitude: 40°39'22''N; verbatimLongitude: 000°9'25''W; **Identification:** identifiedBy: F. J. Peris-Felipo; **Event:** samplingProtocol: Malaise trap; eventDate: 2007-03-12; **Record Level:** institutionCode: ENV
- **Type status:** Other material. **Occurrence:** individualCount: 1; sex: female; **Location:** country: Spain; stateProvince: Castellón; locality: Pobla de Benifassà, Natural Park of Tinença de Benifassà; verbatimElevation: 662 m; verbatimLatitude: 40°39'22''N; verbatimLongitude: 000°9'25''W; **Identification:** identifiedBy: F. J. Peris-Felipo; **Event:** samplingProtocol: Malaise trap; eventDate: 2007-03-26; **Record Level:** institutionCode: ENV
- **Type status:** Other material. **Occurrence:** individualCount: 1; sex: female; **Location:** country: Spain; stateProvince: Castellón; locality: Pobla de Benifassà, Natural Park of Tinença de Benifassà; verbatimElevation: 662 m; verbatimLatitude: 40°39'22''N; verbatimLongitude: 000°9'25''W; **Identification:** identifiedBy: F. J. Peris-Felipo; **Event:** samplingProtocol: Malaise trap; eventDate: 2007-04-02; **Record Level:** institutionCode: ENV
- **Type status:** Other material. **Occurrence:** individualCount: 1; sex: female; **Location:** country: Spain; stateProvince: Castellón; locality: Pobla de Benifassà, Natural Park of Tinença de Benifassà; verbatimElevation: 662 m; verbatimLatitude: 40°39'22''N; verbatimLongitude: 000°9'25''W; **Identification:** identifiedBy: F. J. Peris-Felipo; **Event:** samplingProtocol: Malaise trap; eventDate: 2007-04-23; **Record Level:** institutionCode: ENV
- **Type status:** Other material. **Occurrence:** individualCount: 7; sex: females; **Location:** country: Spain; stateProvince: Castellón; locality: Pobla de Benifassà, Natural Park of Tinença de Benifassà; verbatimElevation: 662 m; verbatimLatitude: 40°39'22''N; verbatimLongitude: 000°9'25''W; **Identification:** identifiedBy: F. J. Peris-Felipo; **Event:** samplingProtocol: Malaise trap; eventDate: 2007-04-30; **Record Level:** institutionCode: ENV
- **Type status:** Other material. **Occurrence:** individualCount: 2; sex: females; **Location:** country: Spain; stateProvince: Castellón; locality: Pobla de Benifassà, Natural Park of Tinença de Benifassà; verbatimElevation: 662 m; verbatimLatitude: 40°39'22''N; verbatimLongitude: 000°9'25''W; **Identification:** identifiedBy: F. J. Peris-Felipo; **Event:** samplingProtocol: Malaise trap; eventDate: 2007-05-14; **Record Level:** institutionCode: ENV
- **Type status:** Other material. **Occurrence:** individualCount: 2; sex: females; **Location:** country: Spain; stateProvince: Castellón; locality: Pobla de Benifassà, Natural Park of Tinença de Benifassà; verbatimElevation: 662 m; verbatimLatitude: 40°39'22''N; verbatimLongitude: 000°9'25''W; **Identification:** identifiedBy: F. J. Peris-Felipo; **Event:** samplingProtocol: Malaise trap; eventDate: 2007-06-04; **Record Level:** institutionCode: ENV
- **Type status:** Other material. **Occurrence:** individualCount: 2; sex: females; **Location:** country: Spain; stateProvince: Castellón; locality: Pobla de Benifassà, Natural Park of Tinença de Benifassà; verbatimElevation: 662 m; verbatimLatitude: 40°39'22''N; verbatimLongitude: 000°9'25''W; **Identification:** identifiedBy: F. J. Peris-Felipo; **Event:** samplingProtocol: Malaise trap; eventDate: 2007-06-11; **Record Level:** institutionCode: ENV
- **Type status:** Other material. **Occurrence:** individualCount: 1; sex: female; **Location:** country: Spain; stateProvince: Castellón; locality: Pobla de Benifassà, Natural Park of Tinença de Benifassà; verbatimElevation: 662 m; verbatimLatitude: 40°39'22''N; verbatimLongitude: 000°9'25''W; **Identification:** identifiedBy: F. J. Peris-Felipo; **Event:** samplingProtocol: Malaise trap; eventDate: 2007-06-18; **Record Level:** institutionCode: ENV
- **Type status:** Other material. **Occurrence:** individualCount: 1; sex: female; **Location:** country: Spain; stateProvince: Castellón; locality: Pobla de Benifassà, Natural Park of Tinença de Benifassà; verbatimElevation: 662 m; verbatimLatitude: 40°39'22''N; verbatimLongitude: 000°9'25''W; **Identification:** identifiedBy: F. J. Peris-Felipo; **Event:** samplingProtocol: Malaise trap; eventDate: 2007-09-24; **Record Level:** institutionCode: ENV
- **Type status:** Other material. **Occurrence:** individualCount: 1; sex: female; **Location:** country: Spain; stateProvince: Castellón; locality: Pobla de Benifassà, Natural Park of Tinença de Benifassà; verbatimElevation: 662 m; verbatimLatitude: 40°39'22''N; verbatimLongitude: 000°9'25''W; **Identification:** identifiedBy: F. J. Peris-Felipo; **Event:** samplingProtocol: Malaise trap; eventDate: 2007-10-08; **Record Level:** institutionCode: ENV
- **Type status:** Other material. **Occurrence:** individualCount: 2; sex: females; **Location:** country: Spain; stateProvince: Castellón; locality: Pobla de Benifassà, Natural Park of Tinença de Benifassà; verbatimElevation: 662 m; verbatimLatitude: 40°39'22''N; verbatimLongitude: 000°9'25''W; **Identification:** identifiedBy: F. J. Peris-Felipo; **Event:** samplingProtocol: Malaise trap; eventDate: 2007-10-08; **Record Level:** institutionCode: ENV
- **Type status:** Other material. **Occurrence:** individualCount: 1; sex: male; **Location:** country: Spain; stateProvince: Castellón; locality: Pobla de Benifassà, Natural Park of Tinença de Benifassà; verbatimElevation: 662 m; verbatimLatitude: 40°39'22''N; verbatimLongitude: 000°9'25''W; **Identification:** identifiedBy: F. J. Peris-Felipo; **Event:** samplingProtocol: Malaise trap; eventDate: 2007-10-15; **Record Level:** institutionCode: ENV
- **Type status:** Other material. **Occurrence:** individualCount: 1; sex: female; **Location:** country: Spain; stateProvince: Castellón; locality: Pobla de Benifassà, Natural Park of Tinença de Benifassà; verbatimElevation: 662 m; verbatimLatitude: 40°39'22''N; verbatimLongitude: 000°9'25''W; **Identification:** identifiedBy: F. J. Peris-Felipo; **Event:** samplingProtocol: Malaise trap; eventDate: 2007-10-22; **Record Level:** institutionCode: ENV
- **Type status:** Other material. **Occurrence:** individualCount: 2; sex: females; **Location:** country: Spain; stateProvince: Castellón; locality: Pobla de Benifassà, Natural Park of Tinença de Benifassà; verbatimElevation: 662 m; verbatimLatitude: 40°39'22''N; verbatimLongitude: 000°9'25''W; **Identification:** identifiedBy: F. J. Peris-Felipo; **Event:** samplingProtocol: Malaise trap; eventDate: 2007-10-22; **Record Level:** institutionCode: ENV
- **Type status:** Other material. **Occurrence:** individualCount: 3; sex: females; **Location:** country: Spain; stateProvince: Castellón; locality: Pobla de Benifassà, Natural Park of Tinença de Benifassà; verbatimElevation: 662 m; verbatimLatitude: 40°39'22''N; verbatimLongitude: 000°9'25''W; **Identification:** identifiedBy: F. J. Peris-Felipo; **Event:** samplingProtocol: Malaise trap; eventDate: 2007-11-19; **Record Level:** institutionCode: ENV
- **Type status:** Other material. **Occurrence:** individualCount: 1; sex: female; **Location:** country: Spain; stateProvince: Castellón; locality: Pobla de Benifassà, Natural Park of Tinença de Benifassà; verbatimElevation: 662 m; verbatimLatitude: 40°39'22''N; verbatimLongitude: 000°9'25''W; **Identification:** identifiedBy: F. J. Peris-Felipo; **Event:** samplingProtocol: Malaise trap; eventDate: 2007-11-26; **Record Level:** institutionCode: ENV

#### Distribution

Former Czechoslovakia, Denmark, Hungary, Italy, Macedonia, Spain **(new record)**, Sweden and former Yugoslavia.

### Dinotrema crassicosta

(Thomson, 1895)

#### Materials

- **Type status:** Other material. **Occurrence:** individualCount: 2; sex: females; **Location:** country: Spain; stateProvince: Castellón; locality: Pobla de Benifassà, Natural Park of Tinença de Benifassà; verbatimElevation: 662 m; verbatimLatitude: 40°39'22''N; verbatimLongitude: 000°9'25''W; **Identification:** identifiedBy: F. J. Peris-Felipo; **Event:** samplingProtocol: Malaise trap; eventDate: 2004-06-10; **Record Level:** institutionCode: ENV
- **Type status:** Other material. **Occurrence:** individualCount: 1; sex: female; **Location:** country: Spain; stateProvince: Castellón; locality: Pobla de Benifassà, Natural Park of Tinença de Benifassà; verbatimElevation: 662 m; verbatimLatitude: 40°39'22''N; verbatimLongitude: 000°9'25''W; **Identification:** identifiedBy: F. J. Peris-Felipo; **Event:** samplingProtocol: Malaise trap; eventDate: 2004-06-17; **Record Level:** institutionCode: ENV
- **Type status:** Other material. **Occurrence:** individualCount: 1; sex: female; **Location:** country: Spain; stateProvince: Castellón; locality: Pobla de Benifassà, Natural Park of Tinença de Benifassà; verbatimElevation: 662 m; verbatimLatitude: 40°39'22''N; verbatimLongitude: 000°9'25''W; **Identification:** identifiedBy: F. J. Peris-Felipo; **Event:** samplingProtocol: Malaise trap; eventDate: 2004-08-05; **Record Level:** institutionCode: ENV
- **Type status:** Other material. **Occurrence:** individualCount: 1; sex: female; **Location:** country: Spain; stateProvince: Castellón; locality: Pobla de Benifassà, Natural Park of Tinença de Benifassà; verbatimElevation: 662 m; verbatimLatitude: 40°39'22''N; verbatimLongitude: 000°9'25''W; **Identification:** identifiedBy: F. J. Peris-Felipo; **Event:** samplingProtocol: Malaise trap; eventDate: 2004-09-16; **Record Level:** institutionCode: ENV
- **Type status:** Other material. **Occurrence:** individualCount: 1; sex: female; **Location:** country: Spain; stateProvince: Castellón; locality: Pobla de Benifassà, Natural Park of Tinença de Benifassà; verbatimElevation: 662 m; verbatimLatitude: 40°39'22''N; verbatimLongitude: 000°9'25''W; **Identification:** identifiedBy: F. J. Peris-Felipo; **Event:** samplingProtocol: Malaise trap; eventDate: 2004-10-14; **Record Level:** institutionCode: ENV
- **Type status:** Other material. **Occurrence:** individualCount: 1; sex: female; **Location:** country: Spain; stateProvince: Castellón; locality: Pobla de Benifassà, Natural Park of Tinença de Benifassà; verbatimElevation: 662 m; verbatimLatitude: 40°39'22''N; verbatimLongitude: 000°9'25''W; **Identification:** identifiedBy: F. J. Peris-Felipo; **Event:** samplingProtocol: Malaise trap; eventDate: 2004-10-28; **Record Level:** institutionCode: ENV
- **Type status:** Other material. **Occurrence:** individualCount: 1; sex: female; **Location:** country: Spain; stateProvince: Castellón; locality: Pobla de Benifassà, Natural Park of Tinença de Benifassà; verbatimElevation: 662 m; verbatimLatitude: 40°39'22''N; verbatimLongitude: 000°9'25''W; **Identification:** identifiedBy: F. J. Peris-Felipo; **Event:** samplingProtocol: Malaise trap; eventDate: 2005-06-13; **Record Level:** institutionCode: ENV
- **Type status:** Other material. **Occurrence:** individualCount: 1; sex: female; **Location:** country: Spain; stateProvince: Castellón; locality: Pobla de Benifassà, Natural Park of Tinença de Benifassà; verbatimElevation: 662 m; verbatimLatitude: 40°39'22''N; verbatimLongitude: 000°9'25''W; **Identification:** identifiedBy: F. J. Peris-Felipo; **Event:** samplingProtocol: Malaise trap; eventDate: 2005-09-26; **Record Level:** institutionCode: ENV
- **Type status:** Other material. **Occurrence:** individualCount: 1; sex: female; **Location:** country: Spain; stateProvince: Castellón; locality: Pobla de Benifassà, Natural Park of Tinença de Benifassà; verbatimElevation: 662 m; verbatimLatitude: 40°39'22''N; verbatimLongitude: 000°9'25''W; **Identification:** identifiedBy: F. J. Peris-Felipo; **Event:** samplingProtocol: Malaise trap; eventDate: 2006-04-24; **Record Level:** institutionCode: ENV
- **Type status:** Other material. **Occurrence:** individualCount: 1; sex: female; **Location:** country: Spain; stateProvince: Castellón; locality: Pobla de Benifassà, Natural Park of Tinença de Benifassà; verbatimElevation: 662 m; verbatimLatitude: 40°39'22''N; verbatimLongitude: 000°9'25''W; **Identification:** identifiedBy: F. J. Peris-Felipo; **Event:** samplingProtocol: Malaise trap; eventDate: 2006-05-01; **Record Level:** institutionCode: ENV
- **Type status:** Other material. **Occurrence:** individualCount: 1; sex: male; **Location:** country: Spain; stateProvince: Castellón; locality: Pobla de Benifassà, Natural Park of Tinença de Benifassà; verbatimElevation: 662 m; verbatimLatitude: 40°39'22''N; verbatimLongitude: 000°9'25''W; **Identification:** identifiedBy: F. J. Peris-Felipo; **Event:** samplingProtocol: Malaise trap; eventDate: 2006-05-15; **Record Level:** institutionCode: ENV
- **Type status:** Other material. **Occurrence:** individualCount: 2; sex: females; **Location:** country: Spain; stateProvince: Castellón; locality: Pobla de Benifassà, Natural Park of Tinença de Benifassà; verbatimElevation: 662 m; verbatimLatitude: 40°39'22''N; verbatimLongitude: 000°9'25''W; **Identification:** identifiedBy: F. J. Peris-Felipo; **Event:** samplingProtocol: Malaise trap; eventDate: 2006-05-22; **Record Level:** institutionCode: ENV
- **Type status:** Other material. **Occurrence:** individualCount: 1; sex: male; **Location:** country: Spain; stateProvince: Castellón; locality: Pobla de Benifassà, Natural Park of Tinença de Benifassà; verbatimElevation: 662 m; verbatimLatitude: 40°39'22''N; verbatimLongitude: 000°9'25''W; **Identification:** identifiedBy: F. J. Peris-Felipo; **Event:** samplingProtocol: Malaise trap; eventDate: 2006-05-22; **Record Level:** institutionCode: ENV
- **Type status:** Other material. **Occurrence:** individualCount: 1; sex: female; **Location:** country: Spain; stateProvince: Castellón; locality: Pobla de Benifassà, Natural Park of Tinença de Benifassà; verbatimElevation: 662 m; verbatimLatitude: 40°39'22''N; verbatimLongitude: 000°9'25''W; **Identification:** identifiedBy: F. J. Peris-Felipo; **Event:** samplingProtocol: Malaise trap; eventDate: 2006-05-29; **Record Level:** institutionCode: ENV
- **Type status:** Other material. **Occurrence:** individualCount: 1; sex: male; **Location:** country: Spain; stateProvince: Castellón; locality: Pobla de Benifassà, Natural Park of Tinença de Benifassà; verbatimElevation: 662 m; verbatimLatitude: 40°39'22''N; verbatimLongitude: 000°9'25''W; **Identification:** identifiedBy: F. J. Peris-Felipo; **Event:** samplingProtocol: Malaise trap; eventDate: 2006-06-12; **Record Level:** institutionCode: ENV
- **Type status:** Other material. **Occurrence:** individualCount: 1; sex: female; **Location:** country: Spain; stateProvince: Castellón; locality: Pobla de Benifassà, Natural Park of Tinença de Benifassà; verbatimElevation: 662 m; verbatimLatitude: 40°39'22''N; verbatimLongitude: 000°9'25''W; **Identification:** identifiedBy: F. J. Peris-Felipo; **Event:** samplingProtocol: Malaise trap; eventDate: 2006-06-26; **Record Level:** institutionCode: ENV
- **Type status:** Other material. **Occurrence:** individualCount: 1; sex: female; **Location:** country: Spain; stateProvince: Castellón; locality: Pobla de Benifassà, Natural Park of Tinença de Benifassà; verbatimElevation: 662 m; verbatimLatitude: 40°39'22''N; verbatimLongitude: 000°9'25''W; **Identification:** identifiedBy: F. J. Peris-Felipo; **Event:** samplingProtocol: Malaise trap; eventDate: 2006-07-03; **Record Level:** institutionCode: ENV
- **Type status:** Other material. **Occurrence:** individualCount: 1; sex: female; **Location:** country: Spain; stateProvince: Castellón; locality: Pobla de Benifassà, Natural Park of Tinença de Benifassà; verbatimElevation: 662 m; verbatimLatitude: 40°39'22''N; verbatimLongitude: 000°9'25''W; **Identification:** identifiedBy: F. J. Peris-Felipo; **Event:** samplingProtocol: Malaise trap; eventDate: 2006-09-06; **Record Level:** institutionCode: ENV
- **Type status:** Other material. **Occurrence:** individualCount: 1; sex: female; **Location:** country: Spain; stateProvince: Castellón; locality: Pobla de Benifassà, Natural Park of Tinença de Benifassà; verbatimElevation: 662 m; verbatimLatitude: 40°39'22''N; verbatimLongitude: 000°9'25''W; **Identification:** identifiedBy: F. J. Peris-Felipo; **Event:** samplingProtocol: Malaise trap; eventDate: 2006-09-25; **Record Level:** institutionCode: ENV
- **Type status:** Other material. **Occurrence:** individualCount: 1; sex: female; **Location:** country: Spain; stateProvince: Castellón; locality: Pobla de Benifassà, Natural Park of Tinença de Benifassà; verbatimElevation: 662 m; verbatimLatitude: 40°39'22''N; verbatimLongitude: 000°9'25''W; **Identification:** identifiedBy: F. J. Peris-Felipo; **Event:** samplingProtocol: Malaise trap; eventDate: 2006-09-25; **Record Level:** institutionCode: ENV
- **Type status:** Other material. **Occurrence:** individualCount: 2; sex: males; **Location:** country: Spain; stateProvince: Castellón; locality: Pobla de Benifassà, Natural Park of Tinença de Benifassà; verbatimElevation: 662 m; verbatimLatitude: 40°39'22''N; verbatimLongitude: 000°9'25''W; **Identification:** identifiedBy: F. J. Peris-Felipo; **Event:** samplingProtocol: Malaise trap; eventDate: 2006-10-02; **Record Level:** institutionCode: ENV
- **Type status:** Other material. **Occurrence:** individualCount: 1; sex: female; **Location:** country: Spain; stateProvince: Castellón; locality: Pobla de Benifassà, Natural Park of Tinença de Benifassà; verbatimElevation: 662 m; verbatimLatitude: 40°39'22''N; verbatimLongitude: 000°9'25''W; **Identification:** identifiedBy: F. J. Peris-Felipo; **Event:** samplingProtocol: Malaise trap; eventDate: 2007-03-19; **Record Level:** institutionCode: ENV
- **Type status:** Other material. **Occurrence:** individualCount: 1; sex: male; **Location:** country: Spain; stateProvince: Castellón; locality: Pobla de Benifassà, Natural Park of Tinença de Benifassà; verbatimElevation: 662 m; verbatimLatitude: 40°39'22''N; verbatimLongitude: 000°9'25''W; **Identification:** identifiedBy: F. J. Peris-Felipo; **Event:** samplingProtocol: Malaise trap; eventDate: 2007-04-30; **Record Level:** institutionCode: ENV
- **Type status:** Other material. **Occurrence:** individualCount: 1; sex: female; **Location:** country: Spain; stateProvince: Castellón; locality: Pobla de Benifassà, Natural Park of Tinença de Benifassà; verbatimElevation: 662 m; verbatimLatitude: 40°39'22''N; verbatimLongitude: 000°9'25''W; **Identification:** identifiedBy: F. J. Peris-Felipo; **Event:** samplingProtocol: Malaise trap; eventDate: 2007-05-14; **Record Level:** institutionCode: ENV
- **Type status:** Other material. **Occurrence:** individualCount: 1; sex: female; **Location:** country: Spain; stateProvince: Castellón; locality: Pobla de Benifassà, Natural Park of Tinença de Benifassà; verbatimElevation: 662 m; verbatimLatitude: 40°39'22''N; verbatimLongitude: 000°9'25''W; **Identification:** identifiedBy: F. J. Peris-Felipo; **Event:** samplingProtocol: Malaise trap; eventDate: 2007-06-11; **Record Level:** institutionCode: ENV
- **Type status:** Other material. **Occurrence:** individualCount: 2; sex: females; **Location:** country: Spain; stateProvince: Castellón; locality: Pobla de Benifassà, Natural Park of Tinença de Benifassà; verbatimElevation: 662 m; verbatimLatitude: 40°39'22''N; verbatimLongitude: 000°9'25''W; **Identification:** identifiedBy: F. J. Peris-Felipo; **Event:** samplingProtocol: Malaise trap; eventDate: 2007-06-11; **Record Level:** institutionCode: ENV
- **Type status:** Other material. **Occurrence:** individualCount: 1; sex: male; **Location:** country: Spain; stateProvince: Castellón; locality: Pobla de Benifassà, Natural Park of Tinença de Benifassà; verbatimElevation: 662 m; verbatimLatitude: 40°39'22''N; verbatimLongitude: 000°9'25''W; **Identification:** identifiedBy: F. J. Peris-Felipo; **Event:** samplingProtocol: Malaise trap; eventDate: 2007-09-17; **Record Level:** institutionCode: ENV
- **Type status:** Other material. **Occurrence:** individualCount: 1; sex: male; **Location:** country: Spain; stateProvince: Castellón; locality: Pobla de Benifassà, Natural Park of Tinença de Benifassà; verbatimElevation: 662 m; verbatimLatitude: 40°39'22''N; verbatimLongitude: 000°9'25''W; **Identification:** identifiedBy: F. J. Peris-Felipo; **Event:** samplingProtocol: Malaise trap; eventDate: 2007-10-08; **Record Level:** institutionCode: ENV
- **Type status:** Other material. **Occurrence:** individualCount: 1; sex: female; **Location:** country: Spain; stateProvince: Castellón; locality: Pobla de Benifassà, Natural Park of Tinença de Benifassà; verbatimElevation: 662 m; verbatimLatitude: 40°39'22''N; verbatimLongitude: 000°9'25''W; **Identification:** identifiedBy: F. J. Peris-Felipo; **Event:** samplingProtocol: Malaise trap; eventDate: 2007-10-08; **Record Level:** institutionCode: ENV

#### Distribution

Austria, Denmark, Germany, Hungary, Korea, Russia and Spain **(new record)**.

### Dinotrema enanum

Peris-Felipo, 2013

#### Materials

- **Type status:** Holotype. **Occurrence:** individualCount: 1; sex: female; **Location:** country: Spain; stateProvince: Castellón; locality: Pobla de Benifassà, Natural Park of Tinença de Benifassà; verbatimElevation: 662 m; verbatimLatitude: 40°39'22''N; verbatimLongitude: 000°9'25''W; **Identification:** identifiedBy: F. J. Peris-Felipo; **Event:** samplingProtocol: Malaise trap; eventDate: 2006-12-11; **Record Level:** institutionCode: ENV
- **Type status:** Paratype. **Occurrence:** individualCount: 1; sex: male; **Location:** country: Spain; stateProvince: Castellón; locality: Pobla de Benifassà, Natural Park of Tinença de Benifassà; verbatimElevation: 662 m; verbatimLatitude: 40°39'22''N; verbatimLongitude: 000°9'25''W; **Identification:** identifiedBy: F. J. Peris-Felipo; **Event:** samplingProtocol: Malaise trap; eventDate: 2007-05-14; **Record Level:** institutionCode: ENV
- **Type status:** Paratype. **Occurrence:** individualCount: 1; sex: male; **Location:** country: Spain; stateProvince: Alicante; locality: Alcoi, Natural Park of Carrascal de La Font Roja; verbatimElevation: 1072 m; verbatimLatitude: 38°38'51''N; verbatimLongitude: 000°32'46''W; **Identification:** identifiedBy: F. J. Peris-Felipo; **Event:** samplingProtocol: Malaise trap; eventDate: 2006-04-13; **Record Level:** institutionCode: ENV

#### Distribution

Spain.

### Dinotrema fischerianum

Peris-Felipo, 2013

#### Materials

- **Type status:** Holotype. **Occurrence:** individualCount: 1; sex: female; **Location:** country: Spain; stateProvince: Alicante; locality: Alcoi, Natural Park of Carrascal de La Font Roja; verbatimElevation: 1072 m; verbatimLatitude: 38°38'51''N; verbatimLongitude: 000°32'46''W; **Identification:** identifiedBy: F. J. Peris-Felipo; **Event:** samplingProtocol: Malaise trap; eventDate: 2007-04-25; **Record Level:** institutionCode: ENV
- **Type status:** Paratype. **Occurrence:** individualCount: 1; sex: female; **Location:** country: Spain; stateProvince: Alicante; locality: Alcoi, Natural Park of Carrascal de La Font Roja; verbatimElevation: 1072 m; verbatimLatitude: 38°38'51''N; verbatimLongitude: 000°32'46''W; **Identification:** identifiedBy: F. J. Peris-Felipo; **Event:** samplingProtocol: Malaise trap; eventDate: 2004-05-13; **Record Level:** institutionCode: ZISP
- **Type status:** Paratype. **Occurrence:** individualCount: 1; sex: female; **Location:** country: Spain; stateProvince: Castellón; locality: Pobla de Benifassà, Natural Park of Tinença de Benifassà; verbatimElevation: 662 m; verbatimLatitude: 40°39'22''N; verbatimLongitude: 000°9'25''W; **Identification:** identifiedBy: F. J. Peris-Felipo; **Event:** samplingProtocol: Malaise trap; eventDate: 2007-04-23; **Record Level:** institutionCode: ENV

#### Distribution

Spain.

### Dinotrema jimenezi

Peris-Felipo, 2013

#### Materials

- **Type status:** Holotype. **Occurrence:** individualCount: 1; sex: female; **Location:** country: Spain; stateProvince: Castellón; locality: Pobla de Benifassà, Natural Park of Tinença de Benifassà; verbatimElevation: 662 m; verbatimLatitude: 40°39'22''N; verbatimLongitude: 000°9'25''W; **Identification:** identifiedBy: F. J. Peris-Felipo; **Event:** samplingProtocol: Malaise trap; eventDate: 2006-09-25; **Record Level:** institutionCode: ENV
- **Type status:** Paratype. **Occurrence:** individualCount: 1; sex: female; **Location:** country: Spain; stateProvince: Castellón; locality: Pobla de Benifassà, Natural Park of Tinença de Benifassà; verbatimElevation: 662 m; verbatimLatitude: 40°39'22''N; verbatimLongitude: 000°9'25''W; **Identification:** identifiedBy: F. J. Peris-Felipo; **Event:** samplingProtocol: Malaise trap; eventDate: 2006-10-30; **Record Level:** institutionCode: ENV

#### Distribution

Spain.

### Dinotrema lagunasense

Peris-Felipo, 2013

#### Materials

- **Type status:** Holotype. **Occurrence:** individualCount: 1; sex: female; **Location:** country: Spain; stateProvince: Alicante; locality: Torrevieja, Natural Park of Lagunas de La Mata-Torrevieja; verbatimElevation: 6 m; verbatimLatitude: 38°01'49''N; verbatimLongitude: 000°42'00''W; **Identification:** identifiedBy: F. J. Peris-Felipo; **Event:** samplingProtocol: Malaise trap; eventDate: 2005-03-23; **Record Level:** institutionCode: ENV
- **Type status:** Paratype. **Occurrence:** individualCount: 1; sex: male; **Location:** country: Spain; stateProvince: Alicante; locality: Torrevieja, Natural Park of Lagunas de La Mata-Torrevieja; verbatimElevation: 6 m; verbatimLatitude: 38°01'49''N; verbatimLongitude: 000°42'00''W; **Identification:** identifiedBy: F. J. Peris-Felipo; **Event:** samplingProtocol: Malaise trap; eventDate: 2004-11-16; **Record Level:** institutionCode: ENV
- **Type status:** Paratype. **Occurrence:** individualCount: 1; sex: female; **Location:** stateProvince: Alicante; locality: Torrevieja, Natural Park of Lagunas de La Mata-Torrevieja; verbatimElevation: 6 m; verbatimLatitude: 38°01'49''N; verbatimLongitude: 000°42'00''W; **Identification:** identifiedBy: F. J. Peris-Felipo; **Event:** samplingProtocol: Malaise trap; eventDate: 2004-11-23; **Record Level:** institutionCode: ENV
- **Type status:** Paratype. **Occurrence:** individualCount: 1; sex: female; **Location:** stateProvince: Alicante; locality: Torrevieja, Natural Park of Lagunas de La Mata-Torrevieja; verbatimElevation: 6 m; verbatimLatitude: 38°01'49''N; verbatimLongitude: 000°42'00''W; **Identification:** identifiedBy: F. J. Peris-Felipo; **Event:** samplingProtocol: Malaise trap; eventDate: 2004-11-30; **Record Level:** institutionCode: ENV
- **Type status:** Paratype. **Occurrence:** individualCount: 1; sex: male; **Location:** stateProvince: Alicante; locality: Torrevieja, Natural Park of Lagunas de La Mata-Torrevieja; verbatimElevation: 6 m; verbatimLatitude: 38°01'49''N; verbatimLongitude: 000°42'00''W; **Identification:** identifiedBy: F. J. Peris-Felipo; **Event:** samplingProtocol: Malaise trap; eventDate: 2004-11-30; **Record Level:** institutionCode: ENV
- **Type status:** Paratype. **Occurrence:** individualCount: 1; sex: female; **Location:** stateProvince: Alicante; locality: Torrevieja, Natural Park of Lagunas de La Mata-Torrevieja; verbatimElevation: 6 m; verbatimLatitude: 38°01'49''N; verbatimLongitude: 000°42'00''W; **Identification:** identifiedBy: F. J. Peris-Felipo; **Event:** samplingProtocol: Malaise trap; eventDate: 2005-02-02; **Record Level:** institutionCode: ZISP
- **Type status:** Paratype. **Occurrence:** individualCount: 1; sex: female; **Location:** stateProvince: Alicante; locality: Torrevieja, Natural Park of Lagunas de La Mata-Torrevieja; verbatimElevation: 6 m; verbatimLatitude: 38°01'49''N; verbatimLongitude: 000°42'00''W; **Identification:** identifiedBy: F. J. Peris-Felipo; **Event:** samplingProtocol: Malaise trap; eventDate: 2005-03-23; **Record Level:** institutionCode: ZISP
- **Type status:** Paratype. **Occurrence:** individualCount: 1; sex: female; **Location:** stateProvince: Alicante; locality: Torrevieja, Natural Park of Lagunas de La Mata-Torrevieja; verbatimElevation: 6 m; verbatimLatitude: 38°01'49''N; verbatimLongitude: 000°42'00''W; **Identification:** identifiedBy: F. J. Peris-Felipo; **Event:** samplingProtocol: Malaise trap; eventDate: 2005-03-29; **Record Level:** institutionCode: ZISP
- **Type status:** Paratype. **Occurrence:** individualCount: 2; sex: males; **Location:** stateProvince: Alicante; locality: Torrevieja, Natural Park of Lagunas de La Mata-Torrevieja; verbatimElevation: 6 m; verbatimLatitude: 38°01'49''N; verbatimLongitude: 000°42'00''W; **Identification:** identifiedBy: F. J. Peris-Felipo; **Event:** samplingProtocol: Malaise trap; eventDate: 2005-03-29; **Record Level:** institutionCode: ZISP
- **Type status:** Paratype. **Occurrence:** individualCount: 1; sex: male; **Location:** stateProvince: Alicante; locality: Torrevieja, Natural Park of Lagunas de La Mata-Torrevieja; verbatimElevation: 6 m; verbatimLatitude: 38°01'49''N; verbatimLongitude: 000°42'00''W; **Identification:** identifiedBy: F. J. Peris-Felipo; **Event:** samplingProtocol: Malaise trap; eventDate: 2005-04-05; **Record Level:** institutionCode: NHMW
- **Type status:** Paratype. **Occurrence:** individualCount: 1; sex: female; **Location:** stateProvince: Alicante; locality: Torrevieja, Natural Park of Lagunas de La Mata-Torrevieja; verbatimElevation: 6 m; verbatimLatitude: 38°01'49''N; verbatimLongitude: 000°42'00''W; **Identification:** identifiedBy: F. J. Peris-Felipo; **Event:** samplingProtocol: Malaise trap; eventDate: 2005-11-01; **Record Level:** institutionCode: NHMW
- **Type status:** Paratype. **Occurrence:** individualCount: 1; sex: female; **Location:** stateProvince: Alicante; locality: Torrevieja, Natural Park of Lagunas de La Mata-Torrevieja; verbatimElevation: 6 m; verbatimLatitude: 38°01'49''N; verbatimLongitude: 000°42'00''W; **Identification:** identifiedBy: F. J. Peris-Felipo; **Event:** samplingProtocol: Malaise trap; eventDate: 2005-11-15; **Record Level:** institutionCode: BMNH
- **Type status:** Paratype. **Occurrence:** individualCount: 1; sex: male; **Location:** stateProvince: Alicante; locality: Torrevieja, Natural Park of Lagunas de La Mata-Torrevieja; verbatimElevation: 6 m; verbatimLatitude: 38°01'49''N; verbatimLongitude: 000°42'00''W; **Identification:** identifiedBy: F. J. Peris-Felipo; **Event:** samplingProtocol: Malaise trap; eventDate: 2005-11-29; **Record Level:** institutionCode: BMNH
- **Type status:** Paratype. **Occurrence:** individualCount: 1; sex: male; **Location:** stateProvince: Alicante; locality: Torrevieja, Natural Park of Lagunas de La Mata-Torrevieja; verbatimElevation: 6 m; verbatimLatitude: 38°01'49''N; verbatimLongitude: 000°42'00''W; **Identification:** identifiedBy: F. J. Peris-Felipo; **Event:** samplingProtocol: Malaise trap; eventDate: 2006-04-04; **Record Level:** institutionCode: HNHM
- **Type status:** Paratype. **Occurrence:** individualCount: 1; sex: female; **Location:** stateProvince: Alicante; locality: Torrevieja, Natural Park of Lagunas de La Mata-Torrevieja; verbatimElevation: 6 m; verbatimLatitude: 38°01'49''N; verbatimLongitude: 000°42'00''W; **Identification:** identifiedBy: F. J. Peris-Felipo; **Event:** samplingProtocol: Malaise trap; eventDate: 2006-12-05; **Record Level:** institutionCode: HNHM
- **Type status:** Other material. **Occurrence:** individualCount: 1; sex: females; **Location:** stateProvince: Alicante; locality: Torrevieja, Natural Park of Lagunas de La Mata-Torrevieja; verbatimElevation: 6 m; verbatimLatitude: 38°01'49''N; verbatimLongitude: 000°42'00''W; **Identification:** identifiedBy: F. J. Peris-Felipo; **Event:** samplingProtocol: Malaise trap; eventDate: 2004-11-30; **Record Level:** institutionCode: ENV
- **Type status:** Other material. **Occurrence:** individualCount: 1; sex: males; **Location:** stateProvince: Alicante; locality: Torrevieja, Natural Park of Lagunas de La Mata-Torrevieja; verbatimElevation: 6 m; verbatimLatitude: 38°01'49''N; verbatimLongitude: 000°42'00''W; **Identification:** identifiedBy: F. J. Peris-Felipo; **Event:** samplingProtocol: Malaise trap; eventDate: 2004-11-30; **Record Level:** institutionCode: ENV
- **Type status:** Other material. **Occurrence:** individualCount: 3; sex: females; **Location:** stateProvince: Alicante; locality: Torrevieja, Natural Park of Lagunas de La Mata-Torrevieja; verbatimElevation: 6 m; verbatimLatitude: 38°01'49''N; verbatimLongitude: 000°42'00''W; **Identification:** identifiedBy: F. J. Peris-Felipo; **Event:** samplingProtocol: Malaise trap; eventDate: 2005-01-18; **Record Level:** institutionCode: ENV
- **Type status:** Other material. **Occurrence:** individualCount: 1; sex: male; **Location:** stateProvince: Alicante; locality: Torrevieja, Natural Park of Lagunas de La Mata-Torrevieja; verbatimElevation: 6 m; verbatimLatitude: 38°01'49''N; verbatimLongitude: 000°42'00''W; **Identification:** identifiedBy: F. J. Peris-Felipo; **Event:** samplingProtocol: Malaise trap; eventDate: 2006-01-26; **Record Level:** institutionCode: ENV
- **Type status:** Other material. **Occurrence:** individualCount: 1; sex: male; **Location:** stateProvince: Alicante; locality: Torrevieja, Natural Park of Lagunas de La Mata-Torrevieja; verbatimElevation: 6 m; verbatimLatitude: 38°01'49''N; verbatimLongitude: 000°42'00''W; **Identification:** identifiedBy: F. J. Peris-Felipo; **Event:** samplingProtocol: Malaise trap; eventDate: 2005-02-15; **Record Level:** institutionCode: ENV
- **Type status:** Other material. **Occurrence:** individualCount: 1; sex: female; **Location:** stateProvince: Alicante; locality: Torrevieja, Natural Park of Lagunas de La Mata-Torrevieja; verbatimElevation: 6 m; verbatimLatitude: 38°01'49''N; verbatimLongitude: 000°42'00''W; **Identification:** identifiedBy: F. J. Peris-Felipo; **Event:** samplingProtocol: Malaise trap; eventDate: 2005-03-04; **Record Level:** institutionCode: ENV
- **Type status:** Other material. **Occurrence:** individualCount: 1; sex: male; **Location:** stateProvince: Alicante; locality: Torrevieja, Natural Park of Lagunas de La Mata-Torrevieja; verbatimElevation: 6 m; verbatimLatitude: 38°01'49''N; verbatimLongitude: 000°42'00''W; **Identification:** identifiedBy: F. J. Peris-Felipo; **Event:** samplingProtocol: Malaise trap; eventDate: 2005-03-18; **Record Level:** institutionCode: ENV
- **Type status:** Other material. **Occurrence:** individualCount: 4; sex: males; **Location:** stateProvince: Alicante; locality: Torrevieja, Natural Park of Lagunas de La Mata-Torrevieja; verbatimElevation: 6 m; verbatimLatitude: 38°01'49''N; verbatimLongitude: 000°42'00''W; **Identification:** identifiedBy: F. J. Peris-Felipo; **Event:** samplingProtocol: Malaise trap; eventDate: 2005-03-23; **Record Level:** institutionCode: ENV
- **Type status:** Other material. **Occurrence:** individualCount: 7; sex: females; **Location:** stateProvince: Alicante; locality: Torrevieja, Natural Park of Lagunas de La Mata-Torrevieja; verbatimElevation: 6 m; verbatimLatitude: 38°01'49''N; verbatimLongitude: 000°42'00''W; **Identification:** identifiedBy: F. J. Peris-Felipo; **Event:** samplingProtocol: Malaise trap; eventDate: 2005-03-29; **Record Level:** institutionCode: ENV
- **Type status:** Other material. **Occurrence:** individualCount: 3; sex: males; **Location:** stateProvince: Alicante; locality: Torrevieja, Natural Park of Lagunas de La Mata-Torrevieja; verbatimElevation: 6 m; verbatimLatitude: 38°01'49''N; verbatimLongitude: 000°42'00''W; **Identification:** identifiedBy: F. J. Peris-Felipo; **Event:** samplingProtocol: Malaise trap; eventDate: 2005-03-29; **Record Level:** institutionCode: ENV
- **Type status:** Other material. **Occurrence:** individualCount: 1; sex: female; **Location:** stateProvince: Alicante; locality: Torrevieja, Natural Park of Lagunas de La Mata-Torrevieja; verbatimElevation: 6 m; verbatimLatitude: 38°01'49''N; verbatimLongitude: 000°42'00''W; **Identification:** identifiedBy: F. J. Peris-Felipo; **Event:** samplingProtocol: Malaise trap; eventDate: 2005-04-05; **Record Level:** institutionCode: ENV
- **Type status:** Other material. **Occurrence:** individualCount: 1; sex: male; **Location:** stateProvince: Alicante; locality: Torrevieja, Natural Park of Lagunas de La Mata-Torrevieja; verbatimElevation: 6 m; verbatimLatitude: 38°01'49''N; verbatimLongitude: 000°42'00''W; **Identification:** identifiedBy: F. J. Peris-Felipo; **Event:** samplingProtocol: Malaise trap; eventDate: 2005-04-05; **Record Level:** institutionCode: ENV
- **Type status:** Other material. **Occurrence:** individualCount: 1; sex: female; **Location:** stateProvince: Alicante; locality: Torrevieja, Natural Park of Lagunas de La Mata-Torrevieja; verbatimElevation: 6 m; verbatimLatitude: 38°01'49''N; verbatimLongitude: 000°42'00''W; **Identification:** identifiedBy: F. J. Peris-Felipo; **Event:** samplingProtocol: Malaise trap; eventDate: 2005-04-26; **Record Level:** institutionCode: ENV
- **Type status:** Other material. **Occurrence:** individualCount: 1; sex: male; **Location:** stateProvince: Alicante; locality: Torrevieja, Natural Park of Lagunas de La Mata-Torrevieja; verbatimElevation: 6 m; verbatimLatitude: 38°01'49''N; verbatimLongitude: 000°42'00''W; **Identification:** identifiedBy: F. J. Peris-Felipo; **Event:** samplingProtocol: Malaise trap; eventDate: 2005-04-26; **Record Level:** institutionCode: ENV
- **Type status:** Other material. **Occurrence:** individualCount: 1; sex: male; **Location:** stateProvince: Alicante; locality: Torrevieja, Natural Park of Lagunas de La Mata-Torrevieja; verbatimElevation: 6 m; verbatimLatitude: 38°01'49''N; verbatimLongitude: 000°42'00''W; **Identification:** identifiedBy: F. J. Peris-Felipo; **Event:** samplingProtocol: Malaise trap; eventDate: 2005-11-01; **Record Level:** institutionCode: ENV
- **Type status:** Other material. **Occurrence:** individualCount: 4; sex: females; **Location:** stateProvince: Alicante; locality: Torrevieja, Natural Park of Lagunas de La Mata-Torrevieja; verbatimElevation: 6 m; verbatimLatitude: 38°01'49''N; verbatimLongitude: 000°42'00''W; **Identification:** identifiedBy: F. J. Peris-Felipo; **Event:** samplingProtocol: Malaise trap; eventDate: 2005-11-15; **Record Level:** institutionCode: ENV
- **Type status:** Other material. **Occurrence:** individualCount: 1; sex: female; **Location:** stateProvince: Alicante; locality: Torrevieja, Natural Park of Lagunas de La Mata-Torrevieja; verbatimElevation: 6 m; verbatimLatitude: 38°01'49''N; verbatimLongitude: 000°42'00''W; **Identification:** identifiedBy: F. J. Peris-Felipo; **Event:** samplingProtocol: Malaise trap; eventDate: 2005-12-02; **Record Level:** institutionCode: ENV
- **Type status:** Other material. **Occurrence:** individualCount: 2; sex: females; **Location:** stateProvince: Alicante; locality: Torrevieja, Natural Park of Lagunas de La Mata-Torrevieja; verbatimElevation: 6 m; verbatimLatitude: 38°01'49''N; verbatimLongitude: 000°42'00''W; **Identification:** identifiedBy: F. J. Peris-Felipo; **Event:** samplingProtocol: Malaise trap; eventDate: 2005-12-12; **Record Level:** institutionCode: ENV
- **Type status:** Other material. **Occurrence:** individualCount: 1; sex: male; **Location:** stateProvince: Alicante; locality: Torrevieja, Natural Park of Lagunas de La Mata-Torrevieja; verbatimElevation: 6 m; verbatimLatitude: 38°01'49''N; verbatimLongitude: 000°42'00''W; **Identification:** identifiedBy: F. J. Peris-Felipo; **Event:** samplingProtocol: Malaise trap; eventDate: 2005-12-27; **Record Level:** institutionCode: ENV
- **Type status:** Other material. **Occurrence:** individualCount: 1; sex: female; **Location:** stateProvince: Alicante; locality: Torrevieja, Natural Park of Lagunas de La Mata-Torrevieja; verbatimElevation: 6 m; verbatimLatitude: 38°01'49''N; verbatimLongitude: 000°42'00''W; **Identification:** identifiedBy: F. J. Peris-Felipo; **Event:** samplingProtocol: Malaise trap; eventDate: 2006-01-31; **Record Level:** institutionCode: ENV
- **Type status:** Other material. **Occurrence:** individualCount: 1; sex: male; **Location:** stateProvince: Alicante; locality: Torrevieja, Natural Park of Lagunas de La Mata-Torrevieja; verbatimElevation: 6 m; verbatimLatitude: 38°01'49''N; verbatimLongitude: 000°42'00''W; **Identification:** identifiedBy: F. J. Peris-Felipo; **Event:** samplingProtocol: Malaise trap; eventDate: 2006-02-07; **Record Level:** institutionCode: ENV
- **Type status:** Other material. **Occurrence:** individualCount: 1; sex: male; **Location:** stateProvince: Alicante; locality: Torrevieja, Natural Park of Lagunas de La Mata-Torrevieja; verbatimElevation: 6 m; verbatimLatitude: 38°01'49''N; verbatimLongitude: 000°42'00''W; **Identification:** identifiedBy: F. J. Peris-Felipo; **Event:** samplingProtocol: Malaise trap; eventDate: 2006-02-07; **Record Level:** institutionCode: ENV
- **Type status:** Other material. **Occurrence:** individualCount: 3; sex: males; **Location:** stateProvince: Alicante; locality: Torrevieja, Natural Park of Lagunas de La Mata-Torrevieja; verbatimElevation: 6 m; verbatimLatitude: 38°01'49''N; verbatimLongitude: 000°42'00''W; **Identification:** identifiedBy: F. J. Peris-Felipo; **Event:** samplingProtocol: Malaise trap; eventDate: 2006-03-14; **Record Level:** institutionCode: ENV
- **Type status:** Other material. **Occurrence:** individualCount: 2; sex: males; **Location:** stateProvince: Alicante; locality: Torrevieja, Natural Park of Lagunas de La Mata-Torrevieja; verbatimElevation: 6 m; verbatimLatitude: 38°01'49''N; verbatimLongitude: 000°42'00''W; **Identification:** identifiedBy: F. J. Peris-Felipo; **Event:** samplingProtocol: Malaise trap; eventDate: 2006-03-28; **Record Level:** institutionCode: ENV
- **Type status:** Other material. **Occurrence:** individualCount: 4; sex: females; **Location:** stateProvince: Alicante; locality: Torrevieja, Natural Park of Lagunas de La Mata-Torrevieja; verbatimElevation: 6 m; verbatimLatitude: 38°01'49''N; verbatimLongitude: 000°42'00''W; **Identification:** identifiedBy: F. J. Peris-Felipo; **Event:** samplingProtocol: Malaise trap; eventDate: 2006-05-23; **Record Level:** institutionCode: ENV
- **Type status:** Other material. **Occurrence:** individualCount: 2; sex: males; **Location:** stateProvince: Alicante; locality: Torrevieja, Natural Park of Lagunas de La Mata-Torrevieja; verbatimElevation: 6 m; verbatimLatitude: 38°01'49''N; verbatimLongitude: 000°42'00''W; **Identification:** identifiedBy: F. J. Peris-Felipo; **Event:** samplingProtocol: Malaise trap; eventDate: 2006-11-28; **Record Level:** institutionCode: ENV
- **Type status:** Other material. **Occurrence:** individualCount: 5; sex: females; **Location:** stateProvince: Alicante; locality: Torrevieja, Natural Park of Lagunas de La Mata-Torrevieja; verbatimElevation: 6 m; verbatimLatitude: 38°01'49''N; verbatimLongitude: 000°42'00''W; **Identification:** identifiedBy: F. J. Peris-Felipo; **Event:** samplingProtocol: Malaise trap; eventDate: 2006-11-28; **Record Level:** institutionCode: ENV
- **Type status:** Other material. **Occurrence:** individualCount: 2; sex: males; **Location:** stateProvince: Alicante; locality: Torrevieja, Natural Park of Lagunas de La Mata-Torrevieja; verbatimElevation: 6 m; verbatimLatitude: 38°01'49''N; verbatimLongitude: 000°42'00''W; **Identification:** identifiedBy: F. J. Peris-Felipo; **Event:** samplingProtocol: Malaise trap; eventDate: 2006-12-05; **Record Level:** institutionCode: ENV
- **Type status:** Other material. **Occurrence:** individualCount: 1; sex: male; **Location:** stateProvince: Alicante; locality: Torrevieja, Natural Park of Lagunas de La Mata-Torrevieja; verbatimElevation: 6 m; verbatimLatitude: 38°01'49''N; verbatimLongitude: 000°42'00''W; **Identification:** identifiedBy: F. J. Peris-Felipo; **Event:** samplingProtocol: Malaise trap; eventDate: 2006-12-05; **Record Level:** institutionCode: ENV
- **Type status:** Other material. **Occurrence:** individualCount: 1; sex: male; **Location:** stateProvince: Alicante; locality: Torrevieja, Natural Park of Lagunas de La Mata-Torrevieja; verbatimElevation: 6 m; verbatimLatitude: 38°01'49''N; verbatimLongitude: 000°42'00''W; **Identification:** identifiedBy: F. J. Peris-Felipo; **Event:** samplingProtocol: Malaise trap; eventDate: 2007-03-20; **Record Level:** institutionCode: ENV
- **Type status:** Other material. **Occurrence:** individualCount: 1; sex: male; **Location:** stateProvince: Alicante; locality: Torrevieja, Natural Park of Lagunas de La Mata-Torrevieja; verbatimElevation: 6 m; verbatimLatitude: 38°01'49''N; verbatimLongitude: 000°42'00''W; **Identification:** identifiedBy: F. J. Peris-Felipo; **Event:** samplingProtocol: Malaise trap; eventDate: 2007-05-15; **Record Level:** institutionCode: ENV
- **Type status:** Other material. **Occurrence:** individualCount: 1; sex: female; **Location:** stateProvince: Alicante; locality: Torrevieja, Natural Park of Lagunas de La Mata-Torrevieja; verbatimElevation: 6 m; verbatimLatitude: 38°01'49''N; verbatimLongitude: 000°42'00''W; **Identification:** identifiedBy: F. J. Peris-Felipo; **Event:** samplingProtocol: Malaise trap; eventDate: 2007-10-30; **Record Level:** institutionCode: ENV
- **Type status:** Other material. **Occurrence:** individualCount: 1; sex: male; **Location:** stateProvince: Alicante; locality: Torrevieja, Natural Park of Lagunas de La Mata-Torrevieja; verbatimElevation: 6 m; verbatimLatitude: 38°01'49''N; verbatimLongitude: 000°42'00''W; **Identification:** identifiedBy: F. J. Peris-Felipo; **Event:** samplingProtocol: Malaise trap; eventDate: 2007-11-06; **Record Level:** institutionCode: ENV

#### Distribution

Spain.

### Dinotrema mareum

Peris-Felipo, 2013

#### Materials

- **Type status:** Holotype. **Occurrence:** individualCount: 1; sex: female; **Location:** country: Spain; stateProvince: Castellón; locality: Pobla de Benifassà, Natural Park of Tinença de Benifassà; verbatimElevation: 662 m; verbatimLatitude: 40°39'22''N; verbatimLongitude: 000°9'25''W; **Identification:** identifiedBy: F. J. Peris-Felipo; **Event:** samplingProtocol: Malaise trap; eventDate: 2006-05-15; **Record Level:** institutionCode: ENV
- **Type status:** Paratype. **Occurrence:** individualCount: 1; sex: female; **Location:** country: Spain; stateProvince: Castellón; locality: Pobla de Benifassà, Natural Park of Tinença de Benifassà; verbatimElevation: 662 m; verbatimLatitude: 40°39'22''N; verbatimLongitude: 000°9'25''W; **Identification:** identifiedBy: F. J. Peris-Felipo; **Event:** samplingProtocol: Malaise trap; eventDate: 2006-07-03; **Record Level:** institutionCode: ENV

#### Distribution

Spain.

### Dinotrema munki

Peris-Felipo, 2013

#### Materials

- **Type status:** Holotype. **Occurrence:** individualCount: 1; sex: female; **Location:** country: Spain; stateProvince: Castellón; locality: Pobla de Benifassà, Natural Park of Tinença de Benifassà; verbatimElevation: 662 m; verbatimLatitude: 40°39'22''N; verbatimLongitude: 000°9'25''W; **Identification:** identifiedBy: F. J. Peris-Felipo; **Event:** samplingProtocol: Malaise trap; eventDate: 2007-10-15; **Record Level:** institutionCode: ENV
- **Type status:** Paratype. **Occurrence:** individualCount: 1; sex: male; **Location:** country: Spain; stateProvince: Castellón; locality: Pobla de Benifassà, Natural Park of Tinença de Benifassà; verbatimElevation: 662 m; verbatimLatitude: 40°39'22''N; verbatimLongitude: 000°9'25''W; **Identification:** identifiedBy: F. J. Peris-Felipo; **Event:** samplingProtocol: Malaise trap; eventDate: 2004-10-28; **Record Level:** institutionCode: ENV
- **Type status:** Other material. **Occurrence:** individualCount: 1; sex: female; **Location:** country: Spain; stateProvince: Castellón; locality: Pobla de Benifassà, Natural Park of Tinença de Benifassà; verbatimElevation: 662 m; **Identification:** identifiedBy: F. J. Peris-Felipo; **Event:** samplingProtocol: Malaise trap; eventDate: 2006-05-29; **Record Level:** institutionCode: ENV

#### Distribution

Spain.

### Dinotrema pappi

Peris-Felipo, 2013

#### Materials

- **Type status:** Holotype. **Occurrence:** individualCount: 1; sex: female; **Location:** country: Spain; stateProvince: Alicante; locality: Alcoi, Natural Park of Carrascal de La Font Roja; verbatimElevation: 1072 m; verbatimLatitude: 38°38'51''N; verbatimLongitude: 000°32'46''W; **Identification:** identifiedBy: F. J. Peris-Felipo; **Event:** samplingProtocol: Malaise trap; eventDate: 2007-01-02; **Record Level:** institutionCode: ENV
- **Type status:** Paratype. **Occurrence:** individualCount: 1; sex: female; **Location:** country: Spain; stateProvince: Alicante; locality: Alcoi, Natural Park of Carrascal de La Font Roja; verbatimElevation: 1072 m; verbatimLatitude: 38°38'51''N; verbatimLongitude: 000°32'46''W; **Identification:** identifiedBy: F. J. Peris-Felipo; **Event:** samplingProtocol: Malaise trap; eventDate: 2005-01-13; **Record Level:** institutionCode: ENV
- **Type status:** Other material. **Occurrence:** individualCount: 1; sex: female; **Location:** country: Spain; stateProvince: Castellón; locality: Pobla de Benifassà, Natural Park of Tinença de Benifassà; verbatimElevation: 662 m; verbatimLatitude: 40°39'22''N; verbatimLongitude: 000°9'25''W; **Identification:** identifiedBy: F. J. Peris-Felipo; **Event:** samplingProtocol: Malaise trap; eventDate: 2006-05-15; **Record Level:** institutionCode: ENV

#### Distribution

Spain.

### Dinotrema paquitae

Peris-Felipo, 2013

#### Materials

- **Type status:** Holotype. **Occurrence:** individualCount: 1; sex: female; **Location:** country: Spain; stateProvince: Castellón; locality: Pobla de Benifassà, Natural Park of Tinença de Benifassà; verbatimElevation: 662 m; verbatimLatitude: 40°39'22''N; verbatimLongitude: 000°9'25''W; **Identification:** identifiedBy: F. J. Peris-Felipo; **Event:** samplingProtocol: Malaise trap; eventDate: 2006-07-17; **Record Level:** institutionCode: ENV
- **Type status:** Paratype. **Occurrence:** individualCount: 1; sex: female; **Location:** country: Spain; stateProvince: Castellón; locality: Pobla de Benifassà, Natural Park of Tinença de Benifassà; verbatimElevation: 662 m; verbatimLatitude: 40°39'22''N; verbatimLongitude: 000°9'25''W; **Identification:** identifiedBy: F. J. Peris-Felipo; **Event:** samplingProtocol: Malaise trap; eventDate: 2004-10-07; **Record Level:** institutionCode: ENV
- **Type status:** Paratype. **Occurrence:** individualCount: 1; sex: male; **Location:** country: Spain; stateProvince: Castellón; locality: Pobla de Benifassà, Natural Park of Tinença de Benifassà; verbatimElevation: 662 m; verbatimLatitude: 40°39'22''N; verbatimLongitude: 000°9'25''W; **Identification:** identifiedBy: F. J. Peris-Felipo; **Event:** samplingProtocol: Malaise trap; eventDate: 2006-05-29; **Record Level:** institutionCode: ENV
- **Type status:** Paratype. **Occurrence:** individualCount: 1; sex: female; **Location:** country: Spain; stateProvince: Castellón; locality: Pobla de Benifassà, Natural Park of Tinença de Benifassà; verbatimElevation: 662 m; verbatimLatitude: 40°39'22''N; verbatimLongitude: 000°9'25''W; **Identification:** identifiedBy: F. J. Peris-Felipo; **Event:** samplingProtocol: Malaise trap; eventDate: 2007-10-15; **Record Level:** institutionCode: ENV
- **Type status:** Other material. **Occurrence:** individualCount: 1; sex: male; **Location:** country: Spain; stateProvince: Alicante; locality: Torrevieja, Natural Park of Lagunas de La Mata-Torrevieja; verbatimElevation: 6 m; verbatimLatitude: 38°01'49''N; verbatimLongitude: 000°42'00''W; **Identification:** identifiedBy: F. J. Peris-Felipo; **Event:** samplingProtocol: Malaise trap; eventDate: 2004-11-30; **Record Level:** institutionCode: ENV
- **Type status:** Other material. **Occurrence:** individualCount: 1; sex: female; **Location:** country: Spain; stateProvince: Castellón; locality: Pobla de Benifassà, Natural Park of Tinença de Benifassà; verbatimElevation: 662 m; verbatimLatitude: 40°39'22''N; verbatimLongitude: 000°9'25''W; **Identification:** identifiedBy: F. J. Peris-Felipo; **Event:** samplingProtocol: Malaise trap; eventDate: 2006-04-17; **Record Level:** institutionCode: ENV
- **Type status:** Other material. **Occurrence:** individualCount: 1; sex: female; **Location:** country: Spain; stateProvince: Castellón; locality: Pobla de Benifassà, Natural Park of Tinença de Benifassà; verbatimElevation: 662 m; verbatimLatitude: 40°39'22''N; verbatimLongitude: 000°9'25''W; **Identification:** identifiedBy: F. J. Peris-Felipo; **Event:** samplingProtocol: Malaise trap; eventDate: 2006-05-15; **Record Level:** institutionCode: ENV
- **Type status:** Other material. **Occurrence:** individualCount: 1; sex: female; **Location:** country: Spain; stateProvince: Castellón; locality: Pobla de Benifassà, Natural Park of Tinença de Benifassà; verbatimElevation: 662 m; verbatimLatitude: 40°39'22''N; verbatimLongitude: 000°9'25''W; **Identification:** identifiedBy: F. J. Peris-Felipo; **Event:** samplingProtocol: Malaise trap; eventDate: 2007-02-26; **Record Level:** institutionCode: ENV
- **Type status:** Other material. **Occurrence:** individualCount: 1; sex: female; **Location:** country: Spain; stateProvince: Castellón; locality: Pobla de Benifassà, Natural Park of Tinença de Benifassà; verbatimElevation: 662 m; verbatimLatitude: 40°39'22''N; verbatimLongitude: 000°9'25''W; **Identification:** identifiedBy: F. J. Peris-Felipo; **Event:** samplingProtocol: Malaise trap; eventDate: 2007-03-19; **Record Level:** institutionCode: ENV

#### Distribution

Spain.

### Dinotrema parapunctatum

(Fischer, 1976)

#### Materials

- **Type status:** Other material. **Occurrence:** individualCount: 1; sex: female; **Location:** country: Spain; stateProvince: Alicante; locality: Alcoi, Natural Park of Carrascal de La Font Roja; verbatimElevation: 1072 m; verbatimLatitude: 38°38'51''N; verbatimLongitude: 000°32'46''W; **Identification:** identifiedBy: F. J. Peris-Felipo; **Event:** samplingProtocol: Malaise trap; eventDate: 2004-08-02; **Record Level:** institutionCode: ENV
- **Type status:** Other material. **Occurrence:** individualCount: 2; sex: females; **Location:** country: Spain; stateProvince: Alicante; locality: Alcoi, Natural Park of Carrascal de La Font Roja; verbatimElevation: 1072 m; verbatimLatitude: 38°38'51''N; verbatimLongitude: 000°32'46''W; **Identification:** identifiedBy: F. J. Peris-Felipo; **Event:** samplingProtocol: Malaise trap; eventDate: 2004-08-09; **Record Level:** institutionCode: ENV
- **Type status:** Other material. **Occurrence:** individualCount: 1; sex: female; **Location:** country: Spain; stateProvince: Alicante; locality: Alcoi, Natural Park of Carrascal de La Font Roja; verbatimElevation: 1072 m; verbatimLatitude: 38°38'51''N; verbatimLongitude: 000°32'46''W; **Identification:** identifiedBy: F. J. Peris-Felipo; **Event:** samplingProtocol: Malaise trap; eventDate: 2004-08-16; **Record Level:** institutionCode: ENV
- **Type status:** Other material. **Occurrence:** individualCount: 1; sex: female; **Location:** country: Spain; stateProvince: Alicante; locality: Alcoi, Natural Park of Carrascal de La Font Roja; verbatimElevation: 1072 m; verbatimLatitude: 38°38'51''N; verbatimLongitude: 000°32'46''W; **Identification:** identifiedBy: F. J. Peris-Felipo; **Event:** samplingProtocol: Malaise trap; eventDate: 2004-08-23; **Record Level:** institutionCode: ENV
- **Type status:** Other material. **Occurrence:** individualCount: 2; sex: females; **Location:** country: Spain; stateProvince: Alicante; locality: Alcoi, Natural Park of Carrascal de La Font Roja; verbatimElevation: 1072 m; verbatimLatitude: 38°38'51''N; verbatimLongitude: 000°32'46''W; **Identification:** identifiedBy: F. J. Peris-Felipo; **Event:** samplingProtocol: Malaise trap; eventDate: 2004-08-30; **Record Level:** institutionCode: ENV
- **Type status:** Other material. **Occurrence:** individualCount: 1; sex: female; **Location:** country: Spain; stateProvince: Alicante; locality: Alcoi, Natural Park of Carrascal de La Font Roja; verbatimElevation: 1072 m; verbatimLatitude: 38°38'51''N; verbatimLongitude: 000°32'46''W; **Identification:** identifiedBy: F. J. Peris-Felipo; **Event:** samplingProtocol: Malaise trap; eventDate: 2004-09-13; **Record Level:** institutionCode: ENV
- **Type status:** Other material. **Occurrence:** individualCount: 1; sex: female; **Location:** country: Spain; stateProvince: Alicante; locality: Alcoi, Natural Park of Carrascal de La Font Roja; verbatimElevation: 1072 m; verbatimLatitude: 38°38'51''N; verbatimLongitude: 000°32'46''W; **Identification:** identifiedBy: F. J. Peris-Felipo; **Event:** samplingProtocol: Malaise trap; eventDate: 2005-06-12; **Record Level:** institutionCode: ENV
- **Type status:** Other material. **Occurrence:** individualCount: 1; sex: female; **Location:** country: Spain; stateProvince: Alicante; locality: Alcoi, Natural Park of Carrascal de La Font Roja; verbatimElevation: 1072 m; verbatimLatitude: 38°38'51''N; verbatimLongitude: 000°32'46''W; **Identification:** identifiedBy: F. J. Peris-Felipo; **Event:** samplingProtocol: Malaise trap; eventDate: 2005-08-29; **Record Level:** institutionCode: ENV
- **Type status:** Other material. **Occurrence:** individualCount: 2; sex: females; **Location:** country: Spain; stateProvince: Alicante; locality: Alcoi, Natural Park of Carrascal de La Font Roja; verbatimElevation: 1072 m; verbatimLatitude: 38°38'51''N; verbatimLongitude: 000°32'46''W; **Identification:** identifiedBy: F. J. Peris-Felipo; **Event:** samplingProtocol: Malaise trap; eventDate: 2005-09-12; **Record Level:** institutionCode: ENV
- **Type status:** Other material. **Occurrence:** individualCount: 1; sex: female; **Location:** country: Spain; stateProvince: Alicante; locality: Alcoi, Natural Park of Carrascal de La Font Roja; verbatimElevation: 1072 m; verbatimLatitude: 38°38'51''N; verbatimLongitude: 000°32'46''W; **Identification:** identifiedBy: F. J. Peris-Felipo; **Event:** samplingProtocol: Malaise trap; eventDate: 2006-07-10; **Record Level:** institutionCode: ENV
- **Type status:** Other material. **Occurrence:** individualCount: 1; sex: female; **Location:** country: Spain; stateProvince: Alicante; locality: Alcoi, Natural Park of Carrascal de La Font Roja; verbatimElevation: 1072 m; verbatimLatitude: 38°38'51''N; verbatimLongitude: 000°32'46''W; **Identification:** identifiedBy: F. J. Peris-Felipo; **Event:** samplingProtocol: Malaise trap; eventDate: 2006-07-25; **Record Level:** institutionCode: ENV
- **Type status:** Other material. **Occurrence:** individualCount: 2; sex: females; **Location:** country: Spain; stateProvince: Alicante; locality: Alcoi, Natural Park of Carrascal de La Font Roja; verbatimElevation: 1072 m; verbatimLatitude: 38°38'51''N; verbatimLongitude: 000°32'46''W; **Identification:** identifiedBy: F. J. Peris-Felipo; **Event:** samplingProtocol: Malaise trap; eventDate: 2006-08-07; **Record Level:** institutionCode: ENV
- **Type status:** Other material. **Occurrence:** individualCount: 2; sex: females; **Location:** country: Spain; stateProvince: Alicante; locality: Alcoi, Natural Park of Carrascal de La Font Roja; verbatimElevation: 1072 m; verbatimLatitude: 38°38'51''N; verbatimLongitude: 000°32'46''W; **Identification:** identifiedBy: F. J. Peris-Felipo; **Event:** samplingProtocol: Malaise trap; eventDate: 2006-08-14; **Record Level:** institutionCode: ENV
- **Type status:** Other material. **Occurrence:** individualCount: 1; sex: female; **Location:** country: Spain; stateProvince: Alicante; locality: Alcoi, Natural Park of Carrascal de La Font Roja; verbatimElevation: 1072 m; verbatimLatitude: 38°38'51''N; verbatimLongitude: 000°32'46''W; **Identification:** identifiedBy: F. J. Peris-Felipo; **Event:** samplingProtocol: Malaise trap; eventDate: 2006-08-21; **Record Level:** institutionCode: ENV
- **Type status:** Other material. **Occurrence:** individualCount: 1; sex: male; **Location:** country: Spain; stateProvince: Alicante; locality: Alcoi, Natural Park of Carrascal de La Font Roja; verbatimElevation: 1072 m; verbatimLatitude: 38°38'51''N; verbatimLongitude: 000°32'46''W; **Identification:** identifiedBy: F. J. Peris-Felipo; **Event:** samplingProtocol: Malaise trap; eventDate: 2006-08-21; **Record Level:** institutionCode: ENV
- **Type status:** Other material. **Occurrence:** individualCount: 1; sex: female; **Location:** country: Spain; stateProvince: Alicante; locality: Alcoi, Natural Park of Carrascal de La Font Roja; verbatimElevation: 1072 m; verbatimLatitude: 38°38'51''N; verbatimLongitude: 000°32'46''W; **Identification:** identifiedBy: F. J. Peris-Felipo; **Event:** samplingProtocol: Malaise trap; eventDate: 2006-08-28; **Record Level:** institutionCode: ENV
- **Type status:** Other material. **Occurrence:** individualCount: 2; sex: females; **Location:** country: Spain; stateProvince: Alicante; locality: Alcoi, Natural Park of Carrascal de La Font Roja; verbatimElevation: 1072 m; verbatimLatitude: 38°38'51''N; verbatimLongitude: 000°32'46''W; **Identification:** identifiedBy: F. J. Peris-Felipo; **Event:** samplingProtocol: Malaise trap; eventDate: 2006-09-11; **Record Level:** institutionCode: ENV
- **Type status:** Other material. **Occurrence:** individualCount: 3; sex: females; **Location:** country: Spain; stateProvince: Alicante; locality: Alcoi, Natural Park of Carrascal de La Font Roja; verbatimElevation: 1072 m; verbatimLatitude: 38°38'51''N; verbatimLongitude: 000°32'46''W; **Identification:** identifiedBy: F. J. Peris-Felipo; **Event:** samplingProtocol: Malaise trap; eventDate: 2006-09-25; **Record Level:** institutionCode: ENV
- **Type status:** Other material. **Occurrence:** individualCount: 1; sex: female; **Location:** country: Spain; stateProvince: Alicante; locality: Alcoi, Natural Park of Carrascal de La Font Roja; verbatimElevation: 1072 m; verbatimLatitude: 38°38'51''N; verbatimLongitude: 000°32'46''W; **Identification:** identifiedBy: F. J. Peris-Felipo; **Event:** samplingProtocol: Malaise trap; eventDate: 2006-10-02; **Record Level:** institutionCode: ENV
- **Type status:** Other material. **Occurrence:** individualCount: 1; sex: male; **Location:** country: Spain; stateProvince: Alicante; locality: Alcoi, Natural Park of Carrascal de La Font Roja; verbatimElevation: 1072 m; verbatimLatitude: 38°38'51''N; verbatimLongitude: 000°32'46''W; **Identification:** identifiedBy: F. J. Peris-Felipo; **Event:** samplingProtocol: Malaise trap; eventDate: 2007-07-02; **Record Level:** institutionCode: ENV
- **Type status:** Other material. **Occurrence:** individualCount: 1; sex: female; **Location:** country: Spain; stateProvince: Alicante; locality: Alcoi, Natural Park of Carrascal de La Font Roja; verbatimElevation: 1072 m; verbatimLatitude: 38°38'51''N; verbatimLongitude: 000°32'46''W; **Identification:** identifiedBy: F. J. Peris-Felipo; **Event:** samplingProtocol: Malaise trap; eventDate: 2007-07-23; **Record Level:** institutionCode: ENV
- **Type status:** Other material. **Occurrence:** individualCount: 1; sex: female; **Location:** country: Spain; stateProvince: Alicante; locality: Alcoi, Natural Park of Carrascal de La Font Roja; verbatimElevation: 1072 m; verbatimLatitude: 38°38'51''N; verbatimLongitude: 000°32'46''W; **Identification:** identifiedBy: F. J. Peris-Felipo; **Event:** samplingProtocol: Malaise trap; eventDate: 2007-07-23; **Record Level:** institutionCode: ENV
- **Type status:** Other material. **Occurrence:** individualCount: 1; sex: female; **Location:** country: Spain; stateProvince: Alicante; locality: Alcoi, Natural Park of Carrascal de La Font Roja; verbatimElevation: 1072 m; verbatimLatitude: 38°38'51''N; verbatimLongitude: 000°32'46''W; **Identification:** identifiedBy: F. J. Peris-Felipo; **Event:** samplingProtocol: Malaise trap; eventDate: 2007-07-30; **Record Level:** institutionCode: ENV
- **Type status:** Other material. **Occurrence:** individualCount: 1; sex: female; **Location:** country: Spain; stateProvince: Castellón; locality: Pobla de Benifassà, Natural Park of Tinença de Benifassà; verbatimElevation: 662 m; verbatimLatitude: 40°39'22''N; verbatimLongitude: 000°9'25''W; **Identification:** identifiedBy: F. J. Peris-Felipo; **Event:** samplingProtocol: Malaise trap; eventDate: 2004-08-12; **Record Level:** institutionCode: ENV
- **Type status:** Other material. **Occurrence:** individualCount: 1; sex: female; **Location:** country: Spain; stateProvince: Castellón; locality: Pobla de Benifassà, Natural Park of Tinença de Benifassà; verbatimElevation: 662 m; verbatimLatitude: 40°39'22''N; verbatimLongitude: 000°9'25''W; **Identification:** identifiedBy: F. J. Peris-Felipo; **Event:** samplingProtocol: Malaise trap; eventDate: 2004-09-02; **Record Level:** institutionCode: ENV
- **Type status:** Other material. **Occurrence:** individualCount: 1; sex: female; **Location:** country: Spain; stateProvince: Castellón; locality: Pobla de Benifassà, Natural Park of Tinença de Benifassà; verbatimElevation: 662 m; verbatimLatitude: 40°39'22''N; verbatimLongitude: 000°9'25''W; **Identification:** identifiedBy: F. J. Peris-Felipo; **Event:** samplingProtocol: Malaise trap; eventDate: 2005-07-25; **Record Level:** institutionCode: ENV
- **Type status:** Other material. **Occurrence:** individualCount: 1; sex: female; **Location:** country: Spain; stateProvince: Castellón; locality: Pobla de Benifassà, Natural Park of Tinença de Benifassà; verbatimElevation: 662 m; verbatimLatitude: 40°39'22''N; verbatimLongitude: 000°9'25''W; **Identification:** identifiedBy: F. J. Peris-Felipo; **Event:** samplingProtocol: Malaise trap; eventDate: 2005-09-26; **Record Level:** institutionCode: ENV
- **Type status:** Other material. **Occurrence:** individualCount: 1; sex: female; **Location:** country: Spain; stateProvince: Castellón; locality: Pobla de Benifassà, Natural Park of Tinença de Benifassà; verbatimElevation: 662 m; verbatimLatitude: 40°39'22''N; verbatimLongitude: 000°9'25''W; **Identification:** identifiedBy: F. J. Peris-Felipo; **Event:** samplingProtocol: Malaise trap; eventDate: 2006-08-28; **Record Level:** institutionCode: ENV

#### Distribution

Spain.

### Dinotrema pareum

Peris-Felipo, 2013

#### Materials

- **Type status:** Holotype. **Occurrence:** individualCount: 1; sex: female; **Location:** country: Spain; stateProvince: Castellón; locality: Pobla de Benifassà, Natural Park of Tinença de Benifassà; verbatimElevation: 662 m; verbatimLatitude: 40°39'22''N; verbatimLongitude: 000°9'25''W; **Identification:** identifiedBy: F. J. Peris-Felipo; **Event:** samplingProtocol: Malaise trap; eventDate: 2006-04-17; **Record Level:** institutionCode: ENV
- **Type status:** Paratype. **Occurrence:** individualCount: 1; sex: female; **Location:** country: Spain; stateProvince: Castellón; locality: Pobla de Benifassà, Natural Park of Tinença de Benifassà; verbatimElevation: 662 m; verbatimLatitude: 40°39'22''N; verbatimLongitude: 000°9'25''W; **Identification:** identifiedBy: F. J. Peris-Felipo; **Event:** samplingProtocol: Malaise trap; eventDate: 2005-10-31; **Record Level:** institutionCode: ENV
- **Type status:** Paratype. **Occurrence:** individualCount: 1; sex: female; **Location:** country: Spain; stateProvince: Castellón; locality: Pobla de Benifassà, Natural Park of Tinença de Benifassà; verbatimElevation: 662 m; verbatimLatitude: 40°39'22''N; verbatimLongitude: 000°9'25''W; **Identification:** identifiedBy: F. J. Peris-Felipo; **Event:** samplingProtocol: Malaise trap; eventDate: 2007-02-26; **Record Level:** institutionCode: ENV
- **Type status:** Paratype. **Occurrence:** individualCount: 1; sex: female; **Location:** country: Spain; stateProvince: Castellón; locality: Pobla de Benifassà, Natural Park of Tinença de Benifassà; verbatimElevation: 662 m; verbatimLatitude: 40°39'22''N; verbatimLongitude: 000°9'25''W; **Identification:** identifiedBy: F. J. Peris-Felipo; **Event:** samplingProtocol: Malaise trap; eventDate: 2007-03-19; **Record Level:** institutionCode: ENV
- **Type status:** Paratype. **Occurrence:** individualCount: 1; sex: female; **Location:** country: Spain; stateProvince: Castellón; locality: Pobla de Benifassà, Natural Park of Tinença de Benifassà; verbatimElevation: 662 m; verbatimLatitude: 40°39'22''N; verbatimLongitude: 000°9'25''W; **Identification:** identifiedBy: F. J. Peris-Felipo; **Event:** samplingProtocol: Malaise trap; eventDate: 2007-04-16; **Record Level:** institutionCode: ENV
- **Type status:** Paratype. **Occurrence:** individualCount: 1; sex: female; **Location:** country: Spain; stateProvince: Castellón; locality: Pobla de Benifassà, Natural Park of Tinença de Benifassà; verbatimElevation: 662 m; verbatimLatitude: 40°39'22''N; verbatimLongitude: 000°9'25''W; **Identification:** identifiedBy: F. J. Peris-Felipo; **Event:** samplingProtocol: Malaise trap; eventDate: 2007-04-23; **Record Level:** institutionCode: ZISP
- **Type status:** Other material. **Occurrence:** individualCount: 1; sex: female; **Location:** country: Spain; stateProvince: Castellón; locality: Pobla de Benifassà, Natural Park of Tinença de Benifassà; verbatimElevation: 662 m; verbatimLatitude: 40°39'22''N; verbatimLongitude: 000°9'25''W; **Identification:** identifiedBy: F. J. Peris-Felipo; **Event:** samplingProtocol: Malaise trap; eventDate: 2004-03-19; **Record Level:** institutionCode: ENV
- **Type status:** Other material. **Occurrence:** individualCount: 1; sex: female; **Location:** country: Spain; stateProvince: Castellón; locality: Pobla de Benifassà, Natural Park of Tinença de Benifassà; verbatimElevation: 662 m; verbatimLatitude: 40°39'22''N; verbatimLongitude: 000°9'25''W; **Identification:** identifiedBy: F. J. Peris-Felipo; **Event:** samplingProtocol: Malaise trap; eventDate: 2006-11-06; **Record Level:** institutionCode: ENV
- **Type status:** Other material. **Occurrence:** individualCount: 1; sex: female; **Location:** country: Spain; stateProvince: Castellón; locality: Pobla de Benifassà, Natural Park of Tinença de Benifassà; verbatimElevation: 662 m; verbatimLatitude: 40°39'22''N; verbatimLongitude: 000°9'25''W; **Identification:** identifiedBy: F. J. Peris-Felipo; **Event:** samplingProtocol: Malaise trap; eventDate: 2007-01-01; **Record Level:** institutionCode: ENV

#### Distribution

Spain.

### Dinotrema pilarae

Peris-Felipo, 2013

#### Materials

- **Type status:** Holotype. **Occurrence:** individualCount: 1; sex: female; **Location:** country: Spain; stateProvince: Alicante; locality: Alcoi, Natural Park of Carrascal de La Font Roja; verbatimElevation: 1072 m; verbatimLatitude: 38°38'51''N; verbatimLongitude: 000°32'46''W; **Identification:** identifiedBy: F. J. Peris-Felipo; **Event:** samplingProtocol: Malaise trap; eventDate: 2005-01-13; **Record Level:** institutionCode: ENV
- **Type status:** Paratype. **Occurrence:** individualCount: 1; sex: female; **Location:** country: Spain; stateProvince: Alicante; locality: Alcoi, Natural Park of Carrascal de La Font Roja; verbatimElevation: 1072 m; verbatimLatitude: 38°38'51''N; verbatimLongitude: 000°32'46''W; **Identification:** identifiedBy: F. J. Peris-Felipo; **Event:** samplingProtocol: Malaise trap; eventDate: 2007-01-02; **Record Level:** institutionCode: ENV

#### Distribution

Spain.

### Dinotrema robertoi

Peris-Felipo, 2013

#### Materials

- **Type status:** Holotype. **Occurrence:** individualCount: 1; sex: female; **Location:** country: Spain; stateProvince: Castellón; locality: Pobla de Benifassà, Natural Park of Tinença de Benifassà; verbatimElevation: 662 m; verbatimLatitude: 40°39'22''N; verbatimLongitude: 000°9'25''W; **Identification:** identifiedBy: F. J. Peris-Felipo; **Event:** samplingProtocol: Malaise trap; eventDate: 2004-07-22; **Record Level:** institutionCode: ENV
- **Type status:** Paratype. **Occurrence:** individualCount: 1; sex: female; **Location:** country: Spain; stateProvince: Castellón; locality: Pobla de Benifassà, Natural Park of Tinença de Benifassà; verbatimElevation: 662 m; verbatimLatitude: 40°39'22''N; verbatimLongitude: 000°9'25''W; **Identification:** identifiedBy: F. J. Peris-Felipo; **Event:** samplingProtocol: Malaise trap; eventDate: 2004-08-05; **Record Level:** institutionCode: ENV
- **Type status:** Paratype. **Occurrence:** individualCount: 1; sex: female; **Location:** country: Spain; stateProvince: Castellón; locality: Pobla de Benifassà, Natural Park of Tinença de Benifassà; verbatimElevation: 662 m; verbatimLatitude: 40°39'22''N; verbatimLongitude: 000°9'25''W; **Identification:** identifiedBy: F. J. Peris-Felipo; **Event:** samplingProtocol: Malaise trap; eventDate: 2004-09-16; **Record Level:** institutionCode: ENV

#### Distribution

Spain.

### Dinotrema teresae

Peris-Felipo, 2013

#### Materials

- **Type status:** Holotype. **Occurrence:** individualCount: 1; sex: female; **Location:** country: Spain; stateProvince: Castellón; locality: Pobla de Benifassà, Natural Park of Tinença de Benifassà; verbatimElevation: 662 m; verbatimLatitude: 40°39'22''N; verbatimLongitude: 000°9'25''W; **Identification:** identifiedBy: F. J. Peris-Felipo; **Event:** samplingProtocol: Malaise trap; eventDate: 2007-04-30; **Record Level:** institutionCode: ENV
- **Type status:** Paratype. **Occurrence:** individualCount: 1; sex: female; **Location:** country: Spain; stateProvince: Castellón; locality: Pobla de Benifassà, Natural Park of Tinença de Benifassà; verbatimElevation: 662 m; verbatimLatitude: 40°39'22''N; verbatimLongitude: 000°9'25''W; **Identification:** identifiedBy: F. J. Peris-Felipo; **Event:** samplingProtocol: Malaise trap; eventDate: 2006-04-24; **Record Level:** institutionCode: ENV

#### Distribution

Spain.

### Dinotrema tinencaense

Peris-Felipo, 2013

#### Materials

- **Type status:** Holotype. **Occurrence:** individualCount: 1; sex: female; **Location:** country: Spain; stateProvince: Castellón; locality: Pobla de Benifassà, Natural Park of Tinença de Benifassà; verbatimElevation: 662 m; verbatimLatitude: 40°39'22''N; verbatimLongitude: 000°9'25''W; **Identification:** identifiedBy: F. J. Peris-Felipo; **Event:** samplingProtocol: Malaise trap; eventDate: 2004-06-10; **Record Level:** institutionCode: ENV
- **Type status:** Paratype. **Occurrence:** individualCount: 1; sex: female; **Location:** country: Spain; stateProvince: Castellón; locality: Pobla de Benifassà, Natural Park of Tinença de Benifassà; verbatimElevation: 662 m; verbatimLatitude: 40°39'22''N; verbatimLongitude: 000°9'25''W; **Identification:** identifiedBy: F. J. Peris-Felipo; **Event:** samplingProtocol: Malaise trap; eventDate: 2004-08-05; **Record Level:** institutionCode: ENV
- **Type status:** Paratype. **Occurrence:** individualCount: 1; sex: female; **Location:** country: Spain; stateProvince: Castellón; locality: Pobla de Benifassà, Natural Park of Tinença de Benifassà; verbatimElevation: 662 m; verbatimLatitude: 40°39'22''N; verbatimLongitude: 000°9'25''W; **Identification:** identifiedBy: F. J. Peris-Felipo; **Event:** samplingProtocol: Malaise trap; eventDate: 2004-09-27; **Record Level:** institutionCode: ENV
- **Type status:** Paratype. **Occurrence:** individualCount: 1; sex: female; **Location:** country: Spain; stateProvince: Castellón; locality: Pobla de Benifassà, Natural Park of Tinença de Benifassà; verbatimElevation: 662 m; verbatimLatitude: 40°39'22''N; verbatimLongitude: 000°9'25''W; **Identification:** identifiedBy: F. J. Peris-Felipo; **Event:** samplingProtocol: Malaise trap; eventDate: 2005-07-04; **Record Level:** institutionCode: ZISP
- **Type status:** Other material. **Occurrence:** individualCount: 1; sex: female; **Location:** country: Spain; stateProvince: Castellón; locality: Pobla de Benifassà, Natural Park of Tinença de Benifassà; verbatimElevation: 662 m; verbatimLatitude: 40°39'22''N; verbatimLongitude: 000°9'25''W; **Identification:** identifiedBy: F. J. Peris-Felipo; **Event:** samplingProtocol: Malaise trap; eventDate: 2004-07-15; **Record Level:** institutionCode: ENV
- **Type status:** Other material. **Occurrence:** individualCount: 2; sex: females; **Location:** country: Spain; stateProvince: Castellón; locality: Pobla de Benifassà, Natural Park of Tinença de Benifassà; verbatimElevation: 662 m; verbatimLatitude: 40°39'22''N; verbatimLongitude: 000°9'25''W; **Identification:** identifiedBy: F. J. Peris-Felipo; **Event:** samplingProtocol: Malaise trap; eventDate: 2005-06-13; **Record Level:** institutionCode: ENV
- **Type status:** Other material. **Occurrence:** individualCount: 1; sex: female; **Location:** country: Spain; stateProvince: Castellón; locality: Pobla de Benifassà, Natural Park of Tinença de Benifassà; verbatimElevation: 662 m; verbatimLatitude: 40°39'22''N; verbatimLongitude: 000°9'25''W; **Identification:** identifiedBy: F. J. Peris-Felipo; **Event:** samplingProtocol: Malaise trap; eventDate: 2005-07-04; **Record Level:** institutionCode: ENV

#### Distribution

Spain.

### Dinotrema torreviejaense

Peris-Felipo, 2013

#### Materials

- **Type status:** Holotype. **Occurrence:** individualCount: 1; sex: female; **Location:** country: Spain; stateProvince: Alicante; locality: Torrevieja, Natural Park of Lagunas de La Mata-Torrevieja; verbatimElevation: 6 m; verbatimLatitude: 38°01'49''N; verbatimLongitude: 000°42'00''W; **Identification:** identifiedBy: F. J. Peris-Felipo; **Event:** samplingProtocol: Malaise trap; eventDate: 2004-04-04; **Record Level:** institutionCode: ENV
- **Type status:** Paratype. **Occurrence:** individualCount: 1; sex: female; **Location:** country: Spain; stateProvince: Alicante; locality: Torrevieja, Natural Park of Lagunas de La Mata-Torrevieja; verbatimElevation: 6 m; verbatimLatitude: 38°01'49''N; verbatimLongitude: 000°42'00''W; **Identification:** identifiedBy: F. J. Peris-Felipo; **Event:** samplingProtocol: Malaise trap; eventDate: 2006-05-09; **Record Level:** institutionCode: ENV

#### Distribution

Spain.

### Dinotrema vitobiasi

Peris-Felipo et Belokobylskij, 2013

#### Materials

- **Type status:** Holotype. **Occurrence:** individualCount: 1; sex: female; **Location:** country: Spain; stateProvince: Alicante; locality: Alcoi, Natural Park of Carrascal de La Font Roja; verbatimElevation: 1072 m; verbatimLatitude: 38°38'51''N; verbatimLongitude: 000°32'46''W; **Identification:** identifiedBy: F. J. Peris-Felipo; **Event:** samplingProtocol: Malaise trap; eventDate: 2006-04-13; **Record Level:** institutionCode: ENV
- **Type status:** Paratype. **Occurrence:** individualCount: 1; sex: female; **Location:** country: Spain; stateProvince: Alicante; locality: Alcoi, Natural Park of Carrascal de La Font Roja; verbatimElevation: 1072 m; verbatimLatitude: 38°38'51''N; verbatimLongitude: 000°32'46''W; **Identification:** identifiedBy: F. J. Peris-Felipo; **Event:** samplingProtocol: Malaise trap; eventDate: 2007-05-28; **Record Level:** institutionCode: ENV

#### Distribution

Spain.

### Dinotrema zimmermannae

Peris-Felipo, 2013

#### Materials

- **Type status:** Holotype. **Occurrence:** individualCount: 1; sex: female; **Location:** country: Spain; stateProvince: Alicante; locality: Alcoi, Natural Park of Carrascal de La Font Roja; verbatimElevation: 1072 m; verbatimLatitude: 38°38'51''N; verbatimLongitude: 000°32'46''W; **Identification:** identifiedBy: F. J. Peris-Felipo; **Event:** samplingProtocol: Malaise trap; eventDate: 2007-06-18; **Record Level:** institutionCode: ENV
- **Type status:** Paratype. **Occurrence:** individualCount: 1; sex: female; **Location:** country: Spain; stateProvince: Castellón; locality: Pobla de Benifassà, Natural Park of Tinença de Benifassà; verbatimElevation: 662 m; verbatimLatitude: 40°39'22''N; verbatimLongitude: 000°9'25''W; **Identification:** identifiedBy: F. J. Peris-Felipo; **Event:** samplingProtocol: Malaise trap; eventDate: 2006-07-17; **Record Level:** institutionCode: ENV
- **Type status:** Paratype. **Occurrence:** individualCount: 1; sex: male; **Location:** country: Spain; stateProvince: Castellón; locality: Pobla de Benifassà, Natural Park of Tinença de Benifassà; verbatimElevation: 662 m; verbatimLatitude: 40°39'22''N; verbatimLongitude: 000°9'25''W; **Identification:** identifiedBy: F. J. Peris-Felipo; **Event:** samplingProtocol: Malaise trap; eventDate: 2006-11-06; **Record Level:** institutionCode: ENV
- **Type status:** Paratype. **Occurrence:** individualCount: 1; sex: female; **Location:** country: Spain; stateProvince: Castellón; locality: Pobla de Benifassà, Natural Park of Tinença de Benifassà; verbatimElevation: 662 m; verbatimLatitude: 40°39'22''N; verbatimLongitude: 000°9'25''W; **Identification:** identifiedBy: F. J. Peris-Felipo; **Event:** samplingProtocol: Malaise trap; eventDate: 2007-04-16; **Record Level:** institutionCode: ZISP
- **Type status:** Paratype. **Occurrence:** individualCount: 1; sex: male; **Location:** country: Spain; stateProvince: Castellón; locality: Pobla de Benifassà, Natural Park of Tinença de Benifassà; verbatimElevation: 662 m; verbatimLatitude: 40°39'22''N; verbatimLongitude: 000°9'25''W; **Identification:** identifiedBy: F. J. Peris-Felipo; **Event:** samplingProtocol: Malaise trap; eventDate: 2007-07-03; **Record Level:** institutionCode: ENV
- **Type status:** Other material. **Occurrence:** individualCount: 3; sex: females; **Location:** country: Spain; stateProvince: Castellón; locality: Pobla de Benifassà, Natural Park of Tinença de Benifassà; verbatimElevation: 662 m; verbatimLatitude: 40°39'22''N; verbatimLongitude: 000°9'25''W; **Identification:** identifiedBy: F. J. Peris-Felipo; **Event:** samplingProtocol: Malaise trap; eventDate: 2006-10-30; **Record Level:** institutionCode: ENV

#### Distribution

Spain.

### Eudinostigma latistigma

(Fischer, 1962)

#### Materials

- **Type status:** Other material. **Occurrence:** individualCount: 2; sex: females; **Location:** country: Spain; stateProvince: Castellón; locality: Pobla de Benifassà, Natural Park of Tinença de Benifassà; verbatimElevation: 662 m; verbatimLatitude: 40°39'22''N; verbatimLongitude: 000°9'25''W; **Identification:** identifiedBy: F. J. Peris-Felipo; **Event:** samplingProtocol: Malaise trap; eventDate: 2004-07-22; **Record Level:** institutionCode: ENV
- **Type status:** Other material. **Occurrence:** individualCount: 1; sex: female; **Location:** country: Spain; stateProvince: Castellón; locality: Pobla de Benifassà, Natural Park of Tinença de Benifassà; verbatimElevation: 662 m; verbatimLatitude: 40°39'22''N; verbatimLongitude: 000°9'25''W; **Identification:** identifiedBy: F. J. Peris-Felipo; **Event:** samplingProtocol: Malaise trap; eventDate: 2004-07-29; **Record Level:** institutionCode: ENV
- **Type status:** Other material. **Occurrence:** individualCount: 1; sex: female; **Location:** country: Spain; stateProvince: Castellón; locality: Pobla de Benifassà, Natural Park of Tinença de Benifassà; verbatimElevation: 662 m; verbatimLatitude: 40°39'22''N; verbatimLongitude: 000°9'25''W; **Identification:** identifiedBy: F. J. Peris-Felipo; **Event:** samplingProtocol: Malaise trap; eventDate: 2005-05-15; **Record Level:** institutionCode: ENV
- **Type status:** Other material. **Occurrence:** individualCount: 1; sex: female; **Location:** country: Spain; stateProvince: Castellón; locality: Pobla de Benifassà, Natural Park of Tinença de Benifassà; verbatimElevation: 662 m; verbatimLatitude: 40°39'22''N; verbatimLongitude: 000°9'25''W; **Identification:** identifiedBy: F. J. Peris-Felipo; **Event:** samplingProtocol: Malaise trap; eventDate: 2005-05-31; **Record Level:** institutionCode: ENV
- **Type status:** Other material. **Occurrence:** individualCount: 2; sex: females; **Location:** country: Spain; stateProvince: Castellón; locality: Pobla de Benifassà, Natural Park of Tinença de Benifassà; verbatimElevation: 662 m; verbatimLatitude: 40°39'22''N; verbatimLongitude: 000°9'25''W; **Identification:** identifiedBy: F. J. Peris-Felipo; **Event:** samplingProtocol: Malaise trap; eventDate: 2005-06-13; **Record Level:** institutionCode: ENV
- **Type status:** Other material. **Occurrence:** individualCount: 1; sex: female; **Location:** country: Spain; stateProvince: Castellón; locality: Pobla de Benifassà, Natural Park of Tinença de Benifassà; verbatimElevation: 662 m; verbatimLatitude: 40°39'22''N; verbatimLongitude: 000°9'25''W; **Identification:** identifiedBy: F. J. Peris-Felipo; **Event:** samplingProtocol: Malaise trap; eventDate: 2005-06-27; **Record Level:** institutionCode: ENV
- **Type status:** Other material. **Occurrence:** individualCount: 1; sex: female; **Location:** country: Spain; stateProvince: Castellón; locality: Pobla de Benifassà, Natural Park of Tinença de Benifassà; verbatimElevation: 662 m; verbatimLatitude: 40°39'22''N; verbatimLongitude: 000°9'25''W; **Identification:** identifiedBy: F. J. Peris-Felipo; **Event:** samplingProtocol: Malaise trap; eventDate: 2007-07-22; **Record Level:** institutionCode: ENV
- **Type status:** Other material. **Occurrence:** individualCount: 1; sex: male; **Location:** country: Spain; stateProvince: Alicante; locality: Torrevieja, Natural Park of Lagunas de La Mata-Torrevieja; verbatimElevation: 6 m; verbatimLatitude: 38°01'49''N; verbatimLongitude: 000°42'00''W; **Identification:** identifiedBy: F. J. Peris-Felipo; **Event:** samplingProtocol: Malaise trap; eventDate: 2007-06-05; **Record Level:** institutionCode: ENV

#### Distribution

Austria, Bulgaria, China, Germany, Hungary, Mongolia, Poland, Spain and Switzerland.

### Orthostigma beyarslani

Fischer, 1995

#### Materials

- **Type status:** Other material. **Occurrence:** individualCount: 1; sex: female; **Location:** country: Spain; stateProvince: Alicante; locality: Torrevieja, Natural Park of Lagunas de La Mata-Torrevieja; verbatimElevation: 6 m; verbatimLatitude: 38°01'49''N; verbatimLongitude: 000°42'00''W; **Identification:** identifiedBy: F. J. Peris-Felipo; **Event:** samplingProtocol: Malaise trap; eventDate: 2004-05-25; **Record Level:** institutionCode: ENV

#### Distribution

Iran, Spain and Turkey.

### Orthostigma laticeps

(Thomson, 1895)

#### Materials

- **Type status:** Other material. **Occurrence:** individualCount: 1; sex: female; **Location:** country: Spain; stateProvince: Alicante; locality: Alcoi, Natural Park of Carrascal de La Font Roja; verbatimElevation: 1072 m; verbatimLatitude: 38°38'51''N; verbatimLongitude: 000°32'46''W; **Identification:** identifiedBy: F. J. Peris-Felipo; **Event:** samplingProtocol: Malaise trap; eventDate: 2004-05-20; **Record Level:** institutionCode: ENV
- **Type status:** Other material. **Occurrence:** individualCount: 1; sex: female; **Location:** country: Spain; stateProvince: Alicante; locality: Alcoi, Natural Park of Carrascal de La Font Roja; verbatimElevation: 1072 m; verbatimLatitude: 38°38'51''N; verbatimLongitude: 000°32'46''W; **Identification:** identifiedBy: F. J. Peris-Felipo; **Event:** samplingProtocol: Malaise trap; eventDate: 2004-05-27; **Record Level:** institutionCode: ENV
- **Type status:** Other material. **Occurrence:** individualCount: 1; sex: female; **Location:** country: Spain; stateProvince: Alicante; locality: Alcoi, Natural Park of Carrascal de La Font Roja; verbatimElevation: 1072 m; verbatimLatitude: 38°38'51''N; verbatimLongitude: 000°32'46''W; **Identification:** identifiedBy: F. J. Peris-Felipo; **Event:** samplingProtocol: Malaise trap; eventDate: 2004-06-03; **Record Level:** institutionCode: ENV
- **Type status:** Other material. **Occurrence:** individualCount: 4; sex: females; **Location:** country: Spain; stateProvince: Alicante; locality: Alcoi, Natural Park of Carrascal de La Font Roja; verbatimElevation: 1072 m; verbatimLatitude: 38°38'51''N; verbatimLongitude: 000°32'46''W; **Identification:** identifiedBy: F. J. Peris-Felipo; **Event:** samplingProtocol: Malaise trap; eventDate: 2004-06-12; **Record Level:** institutionCode: ENV
- **Type status:** Other material. **Occurrence:** individualCount: 2; sex: females; **Location:** country: Spain; stateProvince: Alicante; locality: Alcoi, Natural Park of Carrascal de La Font Roja; verbatimElevation: 1072 m; verbatimLatitude: 38°38'51''N; verbatimLongitude: 000°32'46''W; **Identification:** identifiedBy: F. J. Peris-Felipo; **Event:** samplingProtocol: Malaise trap; eventDate: 2004-06-17; **Record Level:** institutionCode: ENV
- **Type status:** Other material. **Occurrence:** individualCount: 1; sex: female; **Location:** country: Spain; stateProvince: Alicante; locality: Alcoi, Natural Park of Carrascal de La Font Roja; verbatimElevation: 1072 m; verbatimLatitude: 38°38'51''N; verbatimLongitude: 000°32'46''W; **Identification:** identifiedBy: F. J. Peris-Felipo; **Event:** samplingProtocol: Malaise trap; eventDate: 2004-07-01; **Record Level:** institutionCode: ENV
- **Type status:** Other material. **Occurrence:** individualCount: 3; sex: females; **Location:** country: Spain; stateProvince: Alicante; locality: Alcoi, Natural Park of Carrascal de La Font Roja; verbatimElevation: 1072 m; verbatimLatitude: 38°38'51''N; verbatimLongitude: 000°32'46''W; **Identification:** identifiedBy: F. J. Peris-Felipo; **Event:** samplingProtocol: Malaise trap; eventDate: 2004-07-08; **Record Level:** institutionCode: ENV
- **Type status:** Other material. **Occurrence:** individualCount: 2; sex: females; **Location:** country: Spain; stateProvince: Alicante; locality: Alcoi, Natural Park of Carrascal de La Font Roja; verbatimElevation: 1072 m; verbatimLatitude: 38°38'51''N; verbatimLongitude: 000°32'46''W; **Identification:** identifiedBy: F. J. Peris-Felipo; **Event:** samplingProtocol: Malaise trap; eventDate: 2004-07-22; **Record Level:** institutionCode: ENV
- **Type status:** Other material. **Occurrence:** individualCount: 1; sex: female; **Location:** country: Spain; stateProvince: Alicante; locality: Alcoi, Natural Park of Carrascal de La Font Roja; verbatimElevation: 1072 m; verbatimLatitude: 38°38'51''N; verbatimLongitude: 000°32'46''W; **Identification:** identifiedBy: F. J. Peris-Felipo; **Event:** samplingProtocol: Malaise trap; eventDate: 2006-07-03; **Record Level:** institutionCode: ENV
- **Type status:** Other material. **Occurrence:** individualCount: 1; sex: female; **Location:** country: Spain; stateProvince: Alicante; locality: Alcoi, Natural Park of Carrascal de La Font Roja; verbatimElevation: 1072 m; verbatimLatitude: 38°38'51''N; verbatimLongitude: 000°32'46''W; **Identification:** identifiedBy: F. J. Peris-Felipo; **Event:** samplingProtocol: Malaise trap; eventDate: 2006-09-04; **Record Level:** institutionCode: ENV
- **Type status:** Other material. **Occurrence:** individualCount: 1; sex: female; **Location:** country: Spain; stateProvince: Alicante; locality: Alcoi, Natural Park of Carrascal de La Font Roja; verbatimElevation: 1072 m; verbatimLatitude: 38°38'51''N; verbatimLongitude: 000°32'46''W; **Identification:** identifiedBy: F. J. Peris-Felipo; **Event:** samplingProtocol: Malaise trap; eventDate: 2006-09-11; **Record Level:** institutionCode: ENV
- **Type status:** Other material. **Occurrence:** individualCount: 1; sex: female; **Location:** country: Spain; stateProvince: Alicante; locality: Alcoi, Natural Park of Carrascal de La Font Roja; verbatimElevation: 1072 m; verbatimLatitude: 38°38'51''N; verbatimLongitude: 000°32'46''W; **Identification:** identifiedBy: F. J. Peris-Felipo; **Event:** samplingProtocol: Malaise trap; eventDate: 2006-09-25; **Record Level:** institutionCode: ENV
- **Type status:** Other material. **Occurrence:** individualCount: 1; sex: female; **Location:** country: Spain; stateProvince: Alicante; locality: Alcoi, Natural Park of Carrascal de La Font Roja; verbatimElevation: 1072 m; verbatimLatitude: 38°38'51''N; verbatimLongitude: 000°32'46''W; **Identification:** identifiedBy: F. J. Peris-Felipo; **Event:** samplingProtocol: Malaise trap; eventDate: 2006-10-16; **Record Level:** institutionCode: ENV
- **Type status:** Other material. **Occurrence:** individualCount: 1; sex: female; **Location:** country: Spain; stateProvince: Alicante; locality: Alcoi, Natural Park of Carrascal de La Font Roja; verbatimElevation: 1072 m; verbatimLatitude: 38°38'51''N; verbatimLongitude: 000°32'46''W; **Identification:** identifiedBy: F. J. Peris-Felipo; **Event:** samplingProtocol: Malaise trap; eventDate: 2007-05-07; **Record Level:** institutionCode: ENV
- **Type status:** Other material. **Occurrence:** individualCount: 4; sex: females; **Location:** country: Spain; stateProvince: Alicante; locality: Alcoi, Natural Park of Carrascal de La Font Roja; verbatimElevation: 1072 m; verbatimLatitude: 38°38'51''N; verbatimLongitude: 000°32'46''W; **Identification:** identifiedBy: F. J. Peris-Felipo; **Event:** samplingProtocol: Malaise trap; eventDate: 2007-05-21; **Record Level:** institutionCode: ENV
- **Type status:** Other material. **Occurrence:** individualCount: 3; sex: females; **Location:** country: Spain; stateProvince: Alicante; locality: Alcoi, Natural Park of Carrascal de La Font Roja; verbatimElevation: 1072 m; verbatimLatitude: 38°38'51''N; verbatimLongitude: 000°32'46''W; **Identification:** identifiedBy: F. J. Peris-Felipo; **Event:** samplingProtocol: Malaise trap; eventDate: 2007-05-28; **Record Level:** institutionCode: ENV
- **Type status:** Other material. **Occurrence:** individualCount: 5; sex: females; **Location:** country: Spain; stateProvince: Alicante; locality: Alcoi, Natural Park of Carrascal de La Font Roja; verbatimElevation: 1072 m; verbatimLatitude: 38°38'51''N; verbatimLongitude: 000°32'46''W; **Identification:** identifiedBy: F. J. Peris-Felipo; **Event:** samplingProtocol: Malaise trap; eventDate: 2007-06-04; **Record Level:** institutionCode: ENV
- **Type status:** Other material. **Occurrence:** individualCount: 2; sex: females; **Location:** country: Spain; stateProvince: Alicante; locality: Alcoi, Natural Park of Carrascal de La Font Roja; verbatimElevation: 1072 m; verbatimLatitude: 38°38'51''N; verbatimLongitude: 000°32'46''W; **Identification:** identifiedBy: F. J. Peris-Felipo; **Event:** samplingProtocol: Malaise trap; eventDate: 2007-06-11; **Record Level:** institutionCode: ENV
- **Type status:** Other material. **Occurrence:** individualCount: 4; sex: females; **Location:** country: Spain; stateProvince: Alicante; locality: Alcoi, Natural Park of Carrascal de La Font Roja; verbatimElevation: 1072 m; verbatimLatitude: 38°38'51''N; verbatimLongitude: 000°32'46''W; **Identification:** identifiedBy: F. J. Peris-Felipo; **Event:** samplingProtocol: Malaise trap; eventDate: 2007-06-18; **Record Level:** institutionCode: ENV
- **Type status:** Other material. **Occurrence:** individualCount: 4; sex: females; **Location:** country: Spain; stateProvince: Alicante; locality: Alcoi, Natural Park of Carrascal de La Font Roja; verbatimElevation: 1072 m; verbatimLatitude: 38°38'51''N; verbatimLongitude: 000°32'46''W; **Identification:** identifiedBy: F. J. Peris-Felipo; **Event:** samplingProtocol: Malaise trap; eventDate: 2007-06-25; **Record Level:** institutionCode: ENV
- **Type status:** Other material. **Occurrence:** individualCount: 1; sex: female; **Location:** country: Spain; stateProvince: Alicante; locality: Alcoi, Natural Park of Carrascal de La Font Roja; verbatimElevation: 1072 m; verbatimLatitude: 38°38'51''N; verbatimLongitude: 000°32'46''W; **Identification:** identifiedBy: F. J. Peris-Felipo; **Event:** samplingProtocol: Malaise trap; eventDate: 2007-07-02; **Record Level:** institutionCode: ENV
- **Type status:** Other material. **Occurrence:** individualCount: 2; sex: females; **Location:** country: Spain; stateProvince: Alicante; locality: Alcoi, Natural Park of Carrascal de La Font Roja; verbatimElevation: 1072 m; verbatimLatitude: 38°38'51''N; verbatimLongitude: 000°32'46''W; **Identification:** identifiedBy: F. J. Peris-Felipo; **Event:** samplingProtocol: Malaise trap; eventDate: 2007-07-09; **Record Level:** institutionCode: ENV
- **Type status:** Other material. **Occurrence:** individualCount: 4; sex: females; **Location:** country: Spain; stateProvince: Alicante; locality: Alcoi, Natural Park of Carrascal de La Font Roja; verbatimElevation: 1072 m; verbatimLatitude: 38°38'51''N; verbatimLongitude: 000°32'46''W; **Identification:** identifiedBy: F. J. Peris-Felipo; **Event:** samplingProtocol: Malaise trap; eventDate: 2007-07-16; **Record Level:** institutionCode: ENV
- **Type status:** Other material. **Occurrence:** individualCount: 1; sex: female; **Location:** country: Spain; stateProvince: Castellón; locality: Pobla de Benifassà, Natural Park of Tinença de Benifassà; verbatimElevation: 662 m; verbatimLatitude: 40°39'22''N; verbatimLongitude: 000°9'25''W; **Identification:** identifiedBy: F. J. Peris-Felipo; **Event:** samplingProtocol: Malaise trap; eventDate: 2004-05-27; **Record Level:** institutionCode: ENV
- **Type status:** Other material. **Occurrence:** individualCount: 1; sex: female; **Location:** country: Spain; stateProvince: Castellón; locality: Pobla de Benifassà, Natural Park of Tinença de Benifassà; verbatimElevation: 662 m; verbatimLatitude: 40°39'22''N; verbatimLongitude: 000°9'25''W; **Identification:** identifiedBy: F. J. Peris-Felipo; **Event:** samplingProtocol: Malaise trap; eventDate: 2004-08-19; **Record Level:** institutionCode: ENV
- **Type status:** Other material. **Occurrence:** individualCount: 2; sex: females; **Location:** country: Spain; stateProvince: Castellón; locality: Pobla de Benifassà, Natural Park of Tinença de Benifassà; verbatimElevation: 662 m; verbatimLatitude: 40°39'22''N; verbatimLongitude: 000°9'25''W; **Identification:** identifiedBy: F. J. Peris-Felipo; **Event:** samplingProtocol: Malaise trap; eventDate: 2004-09-16; **Record Level:** institutionCode: ENV
- **Type status:** Other material. **Occurrence:** individualCount: 1; sex: female; **Location:** country: Spain; stateProvince: Castellón; locality: Pobla de Benifassà, Natural Park of Tinença de Benifassà; verbatimElevation: 662 m; verbatimLatitude: 40°39'22''N; verbatimLongitude: 000°9'25''W; **Identification:** identifiedBy: F. J. Peris-Felipo; **Event:** samplingProtocol: Malaise trap; eventDate: 2004-10-07; **Record Level:** institutionCode: ENV
- **Type status:** Other material. **Occurrence:** individualCount: 1; sex: female; **Location:** country: Spain; stateProvince: Castellón; locality: Pobla de Benifassà, Natural Park of Tinença de Benifassà; verbatimElevation: 662 m; verbatimLatitude: 40°39'22''N; verbatimLongitude: 000°9'25''W; **Identification:** identifiedBy: F. J. Peris-Felipo; **Event:** samplingProtocol: Malaise trap; eventDate: 2004-10-21; **Record Level:** institutionCode: ENV
- **Type status:** Other material. **Occurrence:** individualCount: 1; sex: female; **Location:** country: Spain; stateProvince: Castellón; locality: Pobla de Benifassà, Natural Park of Tinença de Benifassà; verbatimElevation: 662 m; verbatimLatitude: 40°39'22''N; verbatimLongitude: 000°9'25''W; **Identification:** identifiedBy: F. J. Peris-Felipo; **Event:** samplingProtocol: Malaise trap; eventDate: 2005-06-13; **Record Level:** institutionCode: ENV
- **Type status:** Other material. **Occurrence:** individualCount: 1; sex: female; **Location:** country: Spain; stateProvince: Castellón; locality: Pobla de Benifassà, Natural Park of Tinença de Benifassà; verbatimElevation: 662 m; verbatimLatitude: 40°39'22''N; verbatimLongitude: 000°9'25''W; **Identification:** identifiedBy: F. J. Peris-Felipo; **Event:** samplingProtocol: Malaise trap; eventDate: 2006-05-01; **Record Level:** institutionCode: ENV
- **Type status:** Other material. **Occurrence:** individualCount: 1; sex: female; **Location:** country: Spain; stateProvince: Castellón; locality: Pobla de Benifassà, Natural Park of Tinença de Benifassà; verbatimElevation: 662 m; verbatimLatitude: 40°39'22''N; verbatimLongitude: 000°9'25''W; **Identification:** identifiedBy: F. J. Peris-Felipo; **Event:** samplingProtocol: Malaise trap; eventDate: 2006-06-12; **Record Level:** institutionCode: ENV
- **Type status:** Other material. **Occurrence:** individualCount: 1; sex: female; **Location:** country: Spain; stateProvince: Castellón; locality: Pobla de Benifassà, Natural Park of Tinença de Benifassà; verbatimElevation: 662 m; verbatimLatitude: 40°39'22''N; verbatimLongitude: 000°9'25''W; **Identification:** identifiedBy: F. J. Peris-Felipo; **Event:** samplingProtocol: Malaise trap; eventDate: 2006-06-19; **Record Level:** institutionCode: ENV
- **Type status:** Other material. **Occurrence:** individualCount: 1; sex: female; **Location:** country: Spain; stateProvince: Castellón; locality: Pobla de Benifassà, Natural Park of Tinença de Benifassà; verbatimElevation: 662 m; verbatimLatitude: 40°39'22''N; verbatimLongitude: 000°9'25''W; **Identification:** identifiedBy: F. J. Peris-Felipo; **Event:** samplingProtocol: Malaise trap; eventDate: 2006-06-19; **Record Level:** institutionCode: ENV
- **Type status:** Other material. **Occurrence:** individualCount: 1; sex: female; **Location:** country: Spain; stateProvince: Castellón; locality: Pobla de Benifassà, Natural Park of Tinença de Benifassà; verbatimElevation: 662 m; verbatimLatitude: 40°39'22''N; verbatimLongitude: 000°9'25''W; **Identification:** identifiedBy: F. J. Peris-Felipo; **Event:** samplingProtocol: Malaise trap; eventDate: 2006-08-10; **Record Level:** institutionCode: ENV
- **Type status:** Other material. **Occurrence:** individualCount: 1; sex: female; **Location:** country: Spain; stateProvince: Alicante; locality: Torrevieja, Natural Park of Lagunas de La Mata-Torrevieja; verbatimElevation: 6 m; verbatimLatitude: 38°01'49''N; verbatimLongitude: 000°42'00''W; **Identification:** identifiedBy: F. J. Peris-Felipo; **Event:** samplingProtocol: Malaise trap; eventDate: 2004-12-21; **Record Level:** institutionCode: ENV
- **Type status:** Other material. **Occurrence:** individualCount: 1; sex: female; **Location:** country: Spain; stateProvince: Alicante; locality: Torrevieja, Natural Park of Lagunas de La Mata-Torrevieja; verbatimElevation: 6 m; verbatimLatitude: 38°01'49''N; verbatimLongitude: 000°42'00''W; **Identification:** identifiedBy: F. J. Peris-Felipo; **Event:** samplingProtocol: Malaise trap; eventDate: 2005-01-18; **Record Level:** institutionCode: ENV
- **Type status:** Other material. **Occurrence:** individualCount: 1; sex: female; **Location:** country: Spain; stateProvince: Alicante; locality: Torrevieja, Natural Park of Lagunas de La Mata-Torrevieja; verbatimElevation: 6 m; verbatimLatitude: 38°01'49''N; verbatimLongitude: 000°42'00''W; **Identification:** identifiedBy: F. J. Peris-Felipo; **Event:** samplingProtocol: Malaise trap; eventDate: 2005-03-29; **Record Level:** institutionCode: ENV
- **Type status:** Other material. **Occurrence:** individualCount: 1; sex: male; **Location:** country: Spain; stateProvince: Alicante; locality: Torrevieja, Natural Park of Lagunas de La Mata-Torrevieja; verbatimElevation: 6 m; verbatimLatitude: 38°01'49''N; verbatimLongitude: 000°42'00''W; **Identification:** identifiedBy: F. J. Peris-Felipo; **Event:** samplingProtocol: Malaise trap; eventDate: 2005-04-05; **Record Level:** institutionCode: ENV

#### Distribution

Austria, Belgium, Bulgaria, China, Czech Republic, former Czechoslovakia, Denmark, Germany, Greece, Hungary, Iceland, Italy, Korea, Macedonia, Netherlands, Russia, Spain, Sweden, Switzerland, Turkey, Uzbekistan and former Yugoslavia.

### Orthostigma maculipes

(Haliday, 1838)

#### Materials

- **Type status:** Other material. **Occurrence:** individualCount: 1; sex: female; **Location:** country: Spain; stateProvince: Castellón; locality: Pobal de Benifassà, Natural Park of Tinença de Benifassà; verbatimElevation: 662 m; verbatimLatitude: 40°39'22''N; verbatimLongitude: 000°9'25''W; **Identification:** identifiedBy: F. J. Peris-Felipo; **Event:** samplingProtocol: Malaise trap; eventDate: 2005-09-26; **Record Level:** institutionCode: ENV
- **Type status:** Other material. **Occurrence:** individualCount: 2; sex: females; **Location:** country: Spain; stateProvince: Castellón; locality: Pobal de Benifassà, Natural Park of Tinença de Benifassà; verbatimElevation: 662 m; verbatimLatitude: 40°39'22''N; verbatimLongitude: 000°9'25''W; **Identification:** identifiedBy: F. J. Peris-Felipo; **Event:** samplingProtocol: Malaise trap; eventDate: 2006-05-29; **Record Level:** institutionCode: ENV
- **Type status:** Other material. **Occurrence:** individualCount: 2; sex: females; **Location:** country: Spain; stateProvince: Castellón; locality: Pobal de Benifassà, Natural Park of Tinença de Benifassà; verbatimElevation: 662 m; verbatimLatitude: 40°39'22''N; verbatimLongitude: 000°9'25''W; **Identification:** identifiedBy: F. J. Peris-Felipo; **Event:** samplingProtocol: Malaise trap; eventDate: 2006-06-05; **Record Level:** institutionCode: ENV
- **Type status:** Other material. **Occurrence:** individualCount: 1; sex: female; **Location:** country: Spain; stateProvince: Castellón; locality: Pobal de Benifassà, Natural Park of Tinença de Benifassà; verbatimElevation: 662 m; verbatimLatitude: 40°39'22''N; verbatimLongitude: 000°9'25''W; **Identification:** identifiedBy: F. J. Peris-Felipo; **Event:** samplingProtocol: Malaise trap; eventDate: 2006-06-19; **Record Level:** institutionCode: ENV
- **Type status:** Other material. **Occurrence:** individualCount: 1; sex: female; **Location:** country: Spain; stateProvince: Castellón; locality: Pobal de Benifassà, Natural Park of Tinença de Benifassà; verbatimElevation: 662 m; verbatimLatitude: 40°39'22''N; verbatimLongitude: 000°9'25''W; **Identification:** identifiedBy: F. J. Peris-Felipo; **Event:** samplingProtocol: Malaise trap; eventDate: 2006-07-17; **Record Level:** institutionCode: ENV
- **Type status:** Other material. **Occurrence:** individualCount: 1; sex: female; **Location:** country: Spain; stateProvince: Alicante; locality: Torrevieja, Natural Prak of Lagunas de La Mata-Torrevieja; verbatimElevation: 6 m; verbatimLatitude: 38°01'49''N; verbatimLongitude: 000°42'00''W; **Identification:** identifiedBy: F. J. Peris-Felipo; **Event:** samplingProtocol: Malaise trap; eventDate: 2007-03-13; **Record Level:** institutionCode: ENV

#### Distribution

Austria, Bosnia-Herzegovina, Bulgaria, Czech Republic, Faeroe Islands, Germany, Greece, Hungary, Iran, Ireland, Macedonia, Netherlands, Poland, Russia, Spain, Sweden, Switzerland, Ukraine, United Kingdom, formar Yugoslavia and Serbia.

### Orthostigma pumilum

(Nees, 1834)

#### Materials

- **Type status:** Other material. **Occurrence:** individualCount: 1; sex: female; **Location:** country: Spain; stateProvince: Castellón; locality: Pobal de Benifassà, Natural Park of Tinença de Benifassà; verbatimElevation: 662 m; verbatimLatitude: 40°39'22''N; verbatimLongitude: 000°9'25''W; **Identification:** identifiedBy: F. J. Peris-Felipo; **Event:** samplingProtocol: Malaise trap; eventDate: 2004-06-17; **Record Level:** institutionCode: ENV
- **Type status:** Other material. **Occurrence:** individualCount: 1; sex: female; **Location:** country: Spain; stateProvince: Castellón; locality: Pobal de Benifassà, Natural Park of Tinença de Benifassà; verbatimElevation: 662 m; verbatimLatitude: 40°39'22''N; verbatimLongitude: 000°9'25''W; **Identification:** identifiedBy: F. J. Peris-Felipo; **Event:** samplingProtocol: Malaise trap; eventDate: 2004-07-01; **Record Level:** institutionCode: ENV
- **Type status:** Other material. **Occurrence:** individualCount: 1; sex: female; **Location:** country: Spain; stateProvince: Castellón; locality: Pobal de Benifassà, Natural Park of Tinença de Benifassà; verbatimElevation: 662 m; verbatimLatitude: 40°39'22''N; verbatimLongitude: 000°9'25''W; **Identification:** identifiedBy: F. J. Peris-Felipo; **Event:** samplingProtocol: Malaise trap; eventDate: 2004-07-08; **Record Level:** institutionCode: ENV
- **Type status:** Other material. **Occurrence:** individualCount: 2; sex: females; **Location:** country: Spain; stateProvince: Castellón; locality: Pobal de Benifassà, Natural Park of Tinença de Benifassà; verbatimElevation: 662 m; verbatimLatitude: 40°39'22''N; verbatimLongitude: 000°9'25''W; **Identification:** identifiedBy: F. J. Peris-Felipo; **Event:** samplingProtocol: Malaise trap; eventDate: 2004-07-29; **Record Level:** institutionCode: ENV
- **Type status:** Other material. **Occurrence:** individualCount: 1; sex: female; **Location:** country: Spain; stateProvince: Castellón; locality: Pobal de Benifassà, Natural Park of Tinença de Benifassà; verbatimElevation: 662 m; verbatimLatitude: 40°39'22''N; verbatimLongitude: 000°9'25''W; **Identification:** identifiedBy: F. J. Peris-Felipo; **Event:** samplingProtocol: Malaise trap; eventDate: 2004-08-05; **Record Level:** institutionCode: ENV
- **Type status:** Other material. **Occurrence:** individualCount: 1; sex: female; **Location:** country: Spain; stateProvince: Castellón; locality: Pobal de Benifassà, Natural Park of Tinença de Benifassà; verbatimElevation: 662 m; verbatimLatitude: 40°39'22''N; verbatimLongitude: 000°9'25''W; **Identification:** identifiedBy: F. J. Peris-Felipo; **Event:** samplingProtocol: Malaise trap; eventDate: 2004-09-30; **Record Level:** institutionCode: ENV
- **Type status:** Other material. **Occurrence:** individualCount: 1; sex: female; **Location:** country: Spain; stateProvince: Castellón; locality: Pobal de Benifassà, Natural Park of Tinença de Benifassà; verbatimElevation: 662 m; verbatimLatitude: 40°39'22''N; verbatimLongitude: 000°9'25''W; **Identification:** identifiedBy: F. J. Peris-Felipo; **Event:** samplingProtocol: Malaise trap; eventDate: 2005-10-24; **Record Level:** institutionCode: ENV
- **Type status:** Other material. **Occurrence:** individualCount: 1; sex: female; **Location:** country: Spain; stateProvince: Castellón; locality: Pobal de Benifassà, Natural Park of Tinença de Benifassà; verbatimElevation: 662 m; verbatimLatitude: 40°39'22''N; verbatimLongitude: 000°9'25''W; **Identification:** identifiedBy: F. J. Peris-Felipo; **Event:** samplingProtocol: Malaise trap; eventDate: 2006-06-12; **Record Level:** institutionCode: ENV
- **Type status:** Other material. **Occurrence:** individualCount: 1; sex: female; **Location:** country: Spain; stateProvince: Castellón; locality: Pobal de Benifassà, Natural Park of Tinença de Benifassà; verbatimElevation: 662 m; verbatimLatitude: 40°39'22''N; verbatimLongitude: 000°9'25''W; **Identification:** identifiedBy: F. J. Peris-Felipo; **Event:** samplingProtocol: Malaise trap; eventDate: 2006-10-02; **Record Level:** institutionCode: ENV
- **Type status:** Other material. **Occurrence:** individualCount: 1; sex: female; **Location:** country: Spain; stateProvince: Castellón; locality: Pobal de Benifassà, Natural Park of Tinença de Benifassà; verbatimElevation: 662 m; verbatimLatitude: 40°39'22''N; verbatimLongitude: 000°9'25''W; **Identification:** identifiedBy: F. J. Peris-Felipo; **Event:** samplingProtocol: Malaise trap; eventDate: 2007-03-19; **Record Level:** institutionCode: ENV
- **Type status:** Other material. **Occurrence:** individualCount: 1; sex: female; **Location:** country: Spain; stateProvince: Castellón; locality: Pobal de Benifassà, Natural Park of Tinença de Benifassà; verbatimElevation: 662 m; verbatimLatitude: 40°39'22''N; verbatimLongitude: 000°9'25''W; **Identification:** identifiedBy: F. J. Peris-Felipo; **Event:** samplingProtocol: Malaise trap; eventDate: 2007-05-28; **Record Level:** institutionCode: ENV
- **Type status:** Other material. **Occurrence:** individualCount: 1; sex: female; **Location:** country: Spain; stateProvince: Alicante; locality: Torrevieja, Natural Prak of Lagunas de La Mata-Torrevieja; verbatimElevation: 6 m; verbatimLatitude: 38°01'49''N; verbatimLongitude: 000°42'00''W; **Identification:** identifiedBy: F. J. Peris-Felipo; **Event:** samplingProtocol: Malaise trap; eventDate: 2005-05-31; **Record Level:** institutionCode: ENV

#### Distribution

Austria, Bulgaria, China, Croatia, Czech Republic, forms Czechoslovakia, Denmark, France, Germany, Hungary, Iceland, Ireland, Italy, Lithuania, Madeira Islands, Mongolia, Montenegro, Netherlands, Poland, Russia, Slovakia, Spain, Switzerland, Ukraine, United Kingdom, former Yugoslavia and Serbia.

### Orthostigma sculpturatum

Tobias, 1962

#### Materials

- **Type status:** Other material. **Occurrence:** individualCount: 1; sex: female; **Location:** country: Spain; stateProvince: Castellón; locality: Pobal de Benifassà, Natural Park of Tinença de Benifassà; verbatimElevation: 662 m; verbatimLatitude: 40°39'22''N; verbatimLongitude: 000°9'25''W; **Identification:** identifiedBy: F. J. Peris-Felipo; **Event:** samplingProtocol: Malaise trap; eventDate: 2006-08-28; **Record Level:** institutionCode: ENV

#### Distribution

Austria, Bulgaria, China, Czech Republic, former Czechoslovakia, Germany, Hungary, Russia, Spain, Uzbekistan, former Yugoslavia and Serbia.

### Synaldis berbegalae

Peris-Felipo, 2014

#### Materials

- **Type status:** Holotype. **Occurrence:** individualCount: 1; sex: female; **Location:** country: Spain; stateProvince: Castellón; locality: Pobal de Benifassà, Natural Park of Tinença de Benifassà; verbatimElevation: 662 m; verbatimLatitude: 40°39'22''N; verbatimLongitude: 000°9'25''W; **Identification:** identifiedBy: F. J. Peris-Felipo; **Event:** samplingProtocol: Malaise trap; eventDate: 2004-07-01; **Record Level:** institutionCode: ENV
- **Type status:** Paratype. **Occurrence:** individualCount: 1; sex: female; **Location:** country: Spain; stateProvince: Castellón; locality: Pobal de Benifassà, Natural Park of Tinença de Benifassà; verbatimElevation: 662 m; verbatimLatitude: 40°39'22''N; verbatimLongitude: 000°9'25''W; **Identification:** identifiedBy: F. J. Peris-Felipo; **Event:** samplingProtocol: Malaise trap; eventDate: 2004-07-22; **Record Level:** institutionCode: ENV

#### Distribution

Spain.

### Synaldis concolor

(Nees, 1812)

#### Materials

- **Type status:** Other material. **Occurrence:** individualCount: 1; sex: male; **Location:** country: Spain; stateProvince: Castellón; locality: Pobal de Benifassà, Natural Park of Tinença de Benifassà; verbatimElevation: 662 m; verbatimLatitude: 40°39'22''N; verbatimLongitude: 000°9'25''W; **Identification:** identifiedBy: F. J. Peris-Felipo; **Event:** samplingProtocol: Malaise trap; eventDate: 2005-05-29; **Record Level:** institutionCode: ENV
- **Type status:** Other material. **Occurrence:** individualCount: 1; sex: female; **Location:** country: Spain; stateProvince: Castellón; locality: Pobal de Benifassà, Natural Park of Tinença de Benifassà; verbatimElevation: 662 m; verbatimLatitude: 40°39'22''N; verbatimLongitude: 000°9'25''W; **Identification:** identifiedBy: F. J. Peris-Felipo; **Event:** samplingProtocol: Malaise trap; eventDate: 2005-07-25; **Record Level:** institutionCode: ENV
- **Type status:** Other material. **Occurrence:** individualCount: 1; sex: male; **Location:** country: Spain; stateProvince: Castellón; locality: Pobal de Benifassà, Natural Park of Tinença de Benifassà; verbatimElevation: 662 m; verbatimLatitude: 40°39'22''N; verbatimLongitude: 000°9'25''W; **Identification:** identifiedBy: F. J. Peris-Felipo; **Event:** samplingProtocol: Malaise trap; eventDate: 2006-05-15; **Record Level:** institutionCode: ENV
- **Type status:** Other material. **Occurrence:** individualCount: 1; sex: male; **Location:** country: Spain; stateProvince: Castellón; locality: Pobal de Benifassà, Natural Park of Tinença de Benifassà; verbatimElevation: 662 m; verbatimLatitude: 40°39'22''N; verbatimLongitude: 000°9'25''W; **Identification:** identifiedBy: F. J. Peris-Felipo; **Event:** samplingProtocol: Malaise trap; eventDate: 2006-05-22; **Record Level:** institutionCode: ENV
- **Type status:** Other material. **Occurrence:** individualCount: 2; sex: males; **Location:** country: Spain; stateProvince: Castellón; locality: Pobal de Benifassà, Natural Park of Tinença de Benifassà; verbatimElevation: 662 m; verbatimLatitude: 40°39'22''N; verbatimLongitude: 000°9'25''W; **Identification:** identifiedBy: F. J. Peris-Felipo; **Event:** samplingProtocol: Malaise trap; eventDate: 2006-06-12; **Record Level:** institutionCode: ENV
- **Type status:** Other material. **Occurrence:** individualCount: 1; sex: male; **Location:** country: Spain; stateProvince: Castellón; locality: Pobal de Benifassà, Natural Park of Tinença de Benifassà; verbatimElevation: 662 m; verbatimLatitude: 40°39'22''N; verbatimLongitude: 000°9'25''W; **Identification:** identifiedBy: F. J. Peris-Felipo; **Event:** samplingProtocol: Malaise trap; eventDate: 2007-03-19; **Record Level:** institutionCode: ENV
- **Type status:** Other material. **Occurrence:** individualCount: 1; sex: male; **Location:** country: Spain; stateProvince: Alicante; locality: Torrevieja, Natural Park of Lagunas de La Mata-Torrevieja; verbatimElevation: 6 m; verbatimLatitude: 38°01'49''N; verbatimLongitude: 000°42'00''W; **Identification:** identifiedBy: F. J. Peris-Felipo; **Event:** samplingProtocol: Malaise trap; eventDate: 2005-01-18; **Record Level:** institutionCode: ENV
- **Type status:** Other material. **Occurrence:** individualCount: 1; sex: female; **Location:** country: Spain; stateProvince: Alicante; locality: Torrevieja, Natural Park of Lagunas de La Mata-Torrevieja; verbatimElevation: 6 m; verbatimLatitude: 38°01'49''N; verbatimLongitude: 000°42'00''W; **Identification:** identifiedBy: F. J. Peris-Felipo; **Event:** samplingProtocol: Malaise trap; eventDate: 2005-04-05; **Record Level:** institutionCode: ENV
- **Type status:** Other material. **Occurrence:** individualCount: 1; sex: female; **Location:** country: Spain; stateProvince: Alicante; locality: Torrevieja, Natural Park of Lagunas de La Mata-Torrevieja; verbatimElevation: 6 m; verbatimLatitude: 38°01'49''N; verbatimLongitude: 000°42'00''W; **Identification:** identifiedBy: F. J. Peris-Felipo; **Event:** samplingProtocol: Malaise trap; eventDate: 2006-02-28; **Record Level:** institutionCode: ENV
- **Type status:** Other material. **Occurrence:** individualCount: 1; sex: male; **Location:** country: Spain; stateProvince: Alicante; locality: Torrevieja, Natural Park of Lagunas de La Mata-Torrevieja; verbatimElevation: 6 m; verbatimLatitude: 38°01'49''N; verbatimLongitude: 000°42'00''W; **Identification:** identifiedBy: F. J. Peris-Felipo; **Event:** samplingProtocol: Malaise trap; eventDate: 2007-04-03; **Record Level:** institutionCode: ENV

#### Distribution

Afghanistan, Austria, Bulgaria, Czech Republic, former Czechoslovakia, France, Germany, Greece, Hungary, Iceland, Iran, Ireland, Italy, Korea, Lithuania, Mongolia, Netherlands, Norway, Poland, Russia, Spain, Switzerland, United Kingdom, former Yugoslavia and Serbia.

### Synaldis distracta

(Nees, 1834)

#### Materials

- **Type status:** Other material. **Occurrence:** individualCount: 1; sex: male; **Location:** country: Spain; stateProvince: Alicante; locality: Alcoi, Natural Park of Carrascal de La Font Roja; verbatimElevation: 1072 m; verbatimLatitude: 38°38'51''N; verbatimLongitude: 000°32'46''W; **Identification:** identifiedBy: F. J. Peris-Felipo; **Event:** samplingProtocol: Malaise trap; eventDate: 2006-05-11; **Record Level:** institutionCode: ENV
- **Type status:** Other material. **Occurrence:** individualCount: 1; sex: female; **Location:** country: Spain; stateProvince: Castellón; locality: Pobal de Benifassà, Natural Park of Tinença de Benifassà; verbatimElevation: 662 m; verbatimLatitude: 40°39'22''N; verbatimLongitude: 000°9'25''W; **Identification:** identifiedBy: F. J. Peris-Felipo; **Event:** samplingProtocol: Malaise trap; eventDate: 2004-06-03; **Record Level:** institutionCode: ENV
- **Type status:** Other material. **Occurrence:** individualCount: 1; sex: female; **Location:** country: Spain; stateProvince: Castellón; locality: Pobal de Benifassà, Natural Park of Tinença de Benifassà; verbatimElevation: 662 m; verbatimLatitude: 40°39'22''N; verbatimLongitude: 000°9'25''W; **Identification:** identifiedBy: F. J. Peris-Felipo; **Event:** samplingProtocol: Malaise trap; eventDate: 2004-07-08; **Record Level:** institutionCode: ENV
- **Type status:** Other material. **Occurrence:** individualCount: 1; sex: female; **Location:** country: Spain; stateProvince: Castellón; locality: Pobal de Benifassà, Natural Park of Tinença de Benifassà; verbatimElevation: 662 m; verbatimLatitude: 40°39'22''N; verbatimLongitude: 000°9'25''W; **Identification:** identifiedBy: F. J. Peris-Felipo; **Event:** samplingProtocol: Malaise trap; eventDate: 2004-07-15; **Record Level:** institutionCode: ENV
- **Type status:** Other material. **Occurrence:** individualCount: 2; sex: females; **Location:** country: Spain; stateProvince: Castellón; locality: Pobal de Benifassà, Natural Park of Tinença de Benifassà; verbatimElevation: 662 m; verbatimLatitude: 40°39'22''N; verbatimLongitude: 000°9'25''W; **Identification:** identifiedBy: F. J. Peris-Felipo; **Event:** samplingProtocol: Malaise trap; eventDate: 2005-06-13; **Record Level:** institutionCode: ENV
- **Type status:** Other material. **Occurrence:** individualCount: 1; sex: female; **Location:** country: Spain; stateProvince: Castellón; locality: Pobal de Benifassà, Natural Park of Tinença de Benifassà; verbatimElevation: 662 m; verbatimLatitude: 40°39'22''N; verbatimLongitude: 000°9'25''W; **Identification:** identifiedBy: F. J. Peris-Felipo; **Event:** samplingProtocol: Malaise trap; eventDate: 2006-05-01; **Record Level:** institutionCode: ENV
- **Type status:** Other material. **Occurrence:** individualCount: 1; sex: male; **Location:** country: Spain; stateProvince: Castellón; locality: Pobal de Benifassà, Natural Park of Tinença de Benifassà; verbatimElevation: 662 m; verbatimLatitude: 40°39'22''N; verbatimLongitude: 000°9'25''W; **Identification:** identifiedBy: F. J. Peris-Felipo; **Event:** samplingProtocol: Malaise trap; eventDate: 2006-05-01; **Record Level:** institutionCode: ENV
- **Type status:** Other material. **Occurrence:** individualCount: 2; sex: females; **Location:** country: Spain; stateProvince: Castellón; locality: Pobal de Benifassà, Natural Park of Tinença de Benifassà; verbatimElevation: 662 m; verbatimLatitude: 40°39'22''N; verbatimLongitude: 000°9'25''W; **Identification:** identifiedBy: F. J. Peris-Felipo; **Event:** samplingProtocol: Malaise trap; eventDate: 2006-05-22; **Record Level:** institutionCode: ENV
- **Type status:** Other material. **Occurrence:** individualCount: 1; sex: male; **Location:** country: Spain; stateProvince: Castellón; locality: Pobal de Benifassà, Natural Park of Tinença de Benifassà; verbatimElevation: 662 m; verbatimLatitude: 40°39'22''N; verbatimLongitude: 000°9'25''W; **Identification:** identifiedBy: F. J. Peris-Felipo; **Event:** samplingProtocol: Malaise trap; eventDate: 2006-05-22; **Record Level:** institutionCode: ENV
- **Type status:** Other material. **Occurrence:** individualCount: 1; sex: male; **Location:** country: Spain; stateProvince: Castellón; locality: Pobal de Benifassà, Natural Park of Tinença de Benifassà; verbatimElevation: 662 m; verbatimLatitude: 40°39'22''N; verbatimLongitude: 000°9'25''W; **Identification:** identifiedBy: F. J. Peris-Felipo; **Event:** samplingProtocol: Malaise trap; eventDate: 2006-05-29; **Record Level:** institutionCode: ENV
- **Type status:** Other material. **Occurrence:** individualCount: 1; sex: female; **Location:** country: Spain; stateProvince: Castellón; locality: Pobal de Benifassà, Natural Park of Tinença de Benifassà; verbatimElevation: 662 m; verbatimLatitude: 40°39'22''N; verbatimLongitude: 000°9'25''W; **Identification:** identifiedBy: F. J. Peris-Felipo; **Event:** samplingProtocol: Malaise trap; eventDate: 2007-02-05; **Record Level:** institutionCode: ENV
- **Type status:** Other material. **Occurrence:** individualCount: 1; sex: female; **Location:** country: Spain; stateProvince: Castellón; locality: Pobal de Benifassà, Natural Park of Tinença de Benifassà; verbatimElevation: 662 m; verbatimLatitude: 40°39'22''N; verbatimLongitude: 000°9'25''W; **Identification:** identifiedBy: F. J. Peris-Felipo; **Event:** samplingProtocol: Malaise trap; eventDate: 2007-03-19; **Record Level:** institutionCode: ENV
- **Type status:** Other material. **Occurrence:** individualCount: 1; sex: male; **Location:** country: Spain; stateProvince: Castellón; locality: Pobal de Benifassà, Natural Park of Tinença de Benifassà; verbatimElevation: 662 m; verbatimLatitude: 40°39'22''N; verbatimLongitude: 000°9'25''W; **Identification:** identifiedBy: F. J. Peris-Felipo; **Event:** samplingProtocol: Malaise trap; eventDate: 2007-04-23; **Record Level:** institutionCode: ENV
- **Type status:** Other material. **Occurrence:** individualCount: 1; sex: female; **Location:** country: Spain; stateProvince: Castellón; locality: Pobal de Benifassà, Natural Park of Tinença de Benifassà; verbatimElevation: 662 m; verbatimLatitude: 40°39'22''N; verbatimLongitude: 000°9'25''W; **Identification:** identifiedBy: F. J. Peris-Felipo; **Event:** samplingProtocol: Malaise trap; eventDate: 2007-06-11; **Record Level:** institutionCode: ENV
- **Type status:** Other material. **Occurrence:** individualCount: 2; sex: females; **Location:** country: Spain; stateProvince: Alicante; locality: Torrevieja, Natural Park of Lagunas de La Mata-Torrevieja; verbatimElevation: 6 m; verbatimLatitude: 40°39'22''N; verbatimLongitude: 000°9'25''W; **Identification:** identifiedBy: F. J. Peris-Felipo; **Event:** samplingProtocol: Malaise trap; eventDate: 2004-05-25; **Record Level:** institutionCode: ENV
- **Type status:** Other material. **Occurrence:** individualCount: 3; sex: females; **Location:** country: Spain; stateProvince: Alicante; locality: Torrevieja, Natural Park of Lagunas de La Mata-Torrevieja; verbatimElevation: 6 m; verbatimLatitude: 38°01'49''N; verbatimLongitude: 000°42'00''W; **Identification:** identifiedBy: F. J. Peris-Felipo; **Event:** samplingProtocol: Malaise trap; eventDate: 2004-11-23; **Record Level:** institutionCode: ENV
- **Type status:** Other material. **Occurrence:** individualCount: 1; sex: male; **Location:** country: Spain; stateProvince: Alicante; locality: Torrevieja, Natural Park of Lagunas de La Mata-Torrevieja; verbatimElevation: 6 m; verbatimLatitude: 38°01'49''N; verbatimLongitude: 000°42'00''W; **Identification:** identifiedBy: F. J. Peris-Felipo; **Event:** samplingProtocol: Malaise trap; eventDate: 2004-11-30; **Record Level:** institutionCode: ENV
- **Type status:** Other material. **Occurrence:** individualCount: 1; sex: female; **Location:** country: Spain; stateProvince: Alicante; locality: Torrevieja, Natural Park of Lagunas de La Mata-Torrevieja; verbatimElevation: 6 m; verbatimLatitude: 38°01'49''N; verbatimLongitude: 000°42'00''W; **Identification:** identifiedBy: F. J. Peris-Felipo; **Event:** samplingProtocol: Malaise trap; eventDate: 2005-01-18; **Record Level:** institutionCode: ENV
- **Type status:** Other material. **Occurrence:** individualCount: 2; sex: females; **Location:** country: Spain; stateProvince: Alicante; locality: Torrevieja, Natural Park of Lagunas de La Mata-Torrevieja; verbatimElevation: 6 m; verbatimLatitude: 38°01'49''N; verbatimLongitude: 000°42'00''W; **Identification:** identifiedBy: F. J. Peris-Felipo; **Event:** samplingProtocol: Malaise trap; eventDate: 2005-02-15; **Record Level:** institutionCode: ENV
- **Type status:** Other material. **Occurrence:** individualCount: 1; sex: male; **Location:** country: Spain; stateProvince: Alicante; locality: Torrevieja, Natural Park of Lagunas de La Mata-Torrevieja; verbatimElevation: 6 m; verbatimLatitude: 38°01'49''N; verbatimLongitude: 000°42'00''W; **Identification:** identifiedBy: F. J. Peris-Felipo; **Event:** samplingProtocol: Malaise trap; eventDate: 2005-03-29; **Record Level:** institutionCode: ENV
- **Type status:** Other material. **Occurrence:** individualCount: 2; sex: males; **Location:** country: Spain; stateProvince: Alicante; locality: Torrevieja, Natural Park of Lagunas de La Mata-Torrevieja; verbatimElevation: 6 m; verbatimLatitude: 38°01'49''N; verbatimLongitude: 000°42'00''W; **Identification:** identifiedBy: F. J. Peris-Felipo; **Event:** samplingProtocol: Malaise trap; eventDate: 2005-03-29; **Record Level:** institutionCode: ENV
- **Type status:** Other material. **Occurrence:** individualCount: 1; sex: female; **Location:** country: Spain; stateProvince: Alicante; locality: Torrevieja, Natural Park of Lagunas de La Mata-Torrevieja; verbatimElevation: 6 m; verbatimLatitude: 38°01'49''N; verbatimLongitude: 000°42'00''W; **Identification:** identifiedBy: F. J. Peris-Felipo; **Event:** samplingProtocol: Malaise trap; eventDate: 2005-04-05; **Record Level:** institutionCode: ENV
- **Type status:** Other material. **Occurrence:** individualCount: 1; sex: female; **Location:** country: Spain; stateProvince: Alicante; locality: Torrevieja, Natural Park of Lagunas de La Mata-Torrevieja; verbatimElevation: 6 m; verbatimLatitude: 38°01'49''N; verbatimLongitude: 000°42'00''W; **Identification:** identifiedBy: F. J. Peris-Felipo; **Event:** samplingProtocol: Malaise trap; eventDate: 2005-04-26; **Record Level:** institutionCode: ENV
- **Type status:** Other material. **Occurrence:** individualCount: 1; sex: male; **Location:** country: Spain; stateProvince: Alicante; locality: Torrevieja, Natural Park of Lagunas de La Mata-Torrevieja; verbatimElevation: 6 m; verbatimLatitude: 38°01'49''N; verbatimLongitude: 000°42'00''W; **Identification:** identifiedBy: F. J. Peris-Felipo; **Event:** samplingProtocol: Malaise trap; eventDate: 2005-05-23; **Record Level:** institutionCode: ENV
- **Type status:** Other material. **Occurrence:** individualCount: 1; sex: female; **Location:** country: Spain; stateProvince: Alicante; locality: Torrevieja, Natural Park of Lagunas de La Mata-Torrevieja; verbatimElevation: 6 m; verbatimLatitude: 38°01'49''N; verbatimLongitude: 000°42'00''W; **Identification:** identifiedBy: F. J. Peris-Felipo; **Event:** samplingProtocol: Malaise trap; eventDate: 2005-06-14; **Record Level:** institutionCode: ENV
- **Type status:** Other material. **Occurrence:** individualCount: 1; sex: male; **Location:** country: Spain; stateProvince: Alicante; locality: Torrevieja, Natural Park of Lagunas de La Mata-Torrevieja; verbatimElevation: 6 m; verbatimLatitude: 38°01'49''N; verbatimLongitude: 000°42'00''W; **Identification:** identifiedBy: F. J. Peris-Felipo; **Event:** samplingProtocol: Malaise trap; eventDate: 2006-04-04; **Record Level:** institutionCode: ENV
- **Type status:** Other material. **Occurrence:** individualCount: 3; sex: females; **Location:** country: Spain; stateProvince: Alicante; locality: Torrevieja, Natural Park of Lagunas de La Mata-Torrevieja; verbatimElevation: 6 m; verbatimLatitude: 38°01'49''N; verbatimLongitude: 000°42'00''W; **Identification:** identifiedBy: F. J. Peris-Felipo; **Event:** samplingProtocol: Malaise trap; eventDate: 2007-03-13; **Record Level:** institutionCode: ENV

#### Distribution

Austria, Bulgaria, Canary Islands, China, Croatia, Czech Republic, former Czechoslovakia, Finland, Germany, Greece, Hungary, Iceland, Iran, Ireland, Korea, Lithuania, Madeira Islands, Mongolia, Poland, Romania, Russia, Slovenia, Spain, Sweden, Switzerland, Tunisia, United Kingdom, Uzbekistan and former Yugoslavia.

### Synaldis falcoi

Peris-Felipo, 2014

#### Materials

- **Type status:** Holotype. **Occurrence:** individualCount: 1; sex: female; **Location:** country: Spain; stateProvince: Alicante; locality: Alcoi, Natural Park of Carrascal de La Font Roja; verbatimElevation: 1072 m; verbatimLatitude: 38°38'51''N; verbatimLongitude: 000°32'46''W; **Identification:** identifiedBy: F. J. Peris-Felipo; **Event:** samplingProtocol: Malaise trap; eventDate: 2004-05-27; **Record Level:** institutionCode: ENV
- **Type status:** Paratype. **Occurrence:** individualCount: 1; sex: female; **Location:** country: Spain; stateProvince: Alicante; locality: Alcoi, Natural Park of Carrascal de La Font Roja; verbatimElevation: 1072 m; verbatimLatitude: 38°38'51''N; verbatimLongitude: 000°32'46''W; **Identification:** identifiedBy: F. J. Peris-Felipo; **Event:** samplingProtocol: Malaise trap; eventDate: 2004-06-03; **Record Level:** institutionCode: ENV
- **Type status:** Paratype. **Occurrence:** individualCount: 1; sex: female; **Location:** country: Spain; stateProvince: Alicante; locality: Alcoi, Natural Park of Carrascal de La Font Roja; verbatimElevation: 1072 m; verbatimLatitude: 38°38'51''N; verbatimLongitude: 000°32'46''W; **Identification:** identifiedBy: F. J. Peris-Felipo; **Event:** samplingProtocol: Malaise trap; eventDate: 2004-06-12; **Record Level:** institutionCode: ENV
- **Type status:** Paratype. **Occurrence:** individualCount: 1; sex: female; **Location:** country: Spain; stateProvince: Alicante; locality: Alcoi, Natural Park of Carrascal de La Font Roja; verbatimElevation: 1072 m; verbatimLatitude: 38°38'51''N; verbatimLongitude: 000°32'46''W; **Identification:** identifiedBy: F. J. Peris-Felipo; **Event:** samplingProtocol: Malaise trap; eventDate: 2004-06-19; **Record Level:** institutionCode: ENV
- **Type status:** Paratype. **Occurrence:** individualCount: 1; sex: female; **Location:** country: Spain; stateProvince: Alicante; locality: Alcoi, Natural Park of Carrascal de La Font Roja; verbatimElevation: 1072 m; verbatimLatitude: 38°38'51''N; verbatimLongitude: 000°32'46''W; **Identification:** identifiedBy: F. J. Peris-Felipo; **Event:** samplingProtocol: Malaise trap; eventDate: 2004-07-01; **Record Level:** institutionCode: ENV
- **Type status:** Paratype. **Occurrence:** individualCount: 1; sex: female; **Location:** country: Spain; stateProvince: Alicante; locality: Alcoi, Natural Park of Carrascal de La Font Roja; verbatimElevation: 1072 m; verbatimLatitude: 38°38'51''N; verbatimLongitude: 000°32'46''W; **Identification:** identifiedBy: F. J. Peris-Felipo; **Event:** samplingProtocol: Malaise trap; eventDate: 2004-07-16; **Record Level:** institutionCode: ENV
- **Type status:** Paratype. **Occurrence:** individualCount: 1; sex: female; **Location:** country: Spain; stateProvince: Alicante; locality: Alcoi, Natural Park of Carrascal de La Font Roja; verbatimElevation: 1072 m; verbatimLatitude: 38°38'51''N; verbatimLongitude: 000°32'46''W; **Identification:** identifiedBy: F. J. Peris-Felipo; **Event:** samplingProtocol: Malaise trap; eventDate: 2006-08-14; **Record Level:** institutionCode: ENV
- **Type status:** Paratype. **Occurrence:** individualCount: 1; sex: female; **Location:** country: Spain; stateProvince: Alicante; locality: Alcoi, Natural Park of Carrascal de La Font Roja; verbatimElevation: 1072 m; verbatimLatitude: 38°38'51''N; verbatimLongitude: 000°32'46''W; **Identification:** identifiedBy: F. J. Peris-Felipo; **Event:** samplingProtocol: Malaise trap; eventDate: 2006-08-28; **Record Level:** institutionCode: ENV
- **Type status:** Paratype. **Occurrence:** individualCount: 2; sex: females; **Location:** country: Spain; stateProvince: Alicante; locality: Alcoi, Natural Park of Carrascal de La Font Roja; verbatimElevation: 1072 m; verbatimLatitude: 38°38'51''N; verbatimLongitude: 000°32'46''W; **Identification:** identifiedBy: F. J. Peris-Felipo; **Event:** samplingProtocol: Malaise trap; eventDate: 2006-09-11; **Record Level:** institutionCode: ENV
- **Type status:** Paratype. **Occurrence:** individualCount: 3; sex: females; **Location:** country: Spain; stateProvince: Alicante; locality: Alcoi, Natural Park of Carrascal de La Font Roja; verbatimElevation: 1072 m; verbatimLatitude: 38°38'51''N; verbatimLongitude: 000°32'46''W; **Identification:** identifiedBy: F. J. Peris-Felipo; **Event:** samplingProtocol: Malaise trap; eventDate: 2007-06-04; **Record Level:** institutionCode: ENV
- **Type status:** Paratype. **Occurrence:** individualCount: 1; sex: female; **Location:** country: Spain; stateProvince: Alicante; locality: Alcoi, Natural Park of Carrascal de La Font Roja; verbatimElevation: 1072 m; verbatimLatitude: 38°38'51''N; verbatimLongitude: 000°32'46''W; **Identification:** identifiedBy: F. J. Peris-Felipo; **Event:** samplingProtocol: Malaise trap; eventDate: 2007-06-11; **Record Level:** institutionCode: ENV
- **Type status:** Paratype. **Occurrence:** individualCount: 1; sex: female; **Location:** country: Spain; stateProvince: Alicante; locality: Alcoi, Natural Park of Carrascal de La Font Roja; verbatimElevation: 1072 m; verbatimLatitude: 38°38'51''N; verbatimLongitude: 000°32'46''W; **Identification:** identifiedBy: F. J. Peris-Felipo; **Event:** samplingProtocol: Malaise trap; eventDate: 2007-06-18; **Record Level:** institutionCode: ENV
- **Type status:** Paratype. **Occurrence:** individualCount: 1; sex: female; **Location:** country: Spain; stateProvince: Alicante; locality: Alcoi, Natural Park of Carrascal de La Font Roja; verbatimElevation: 1072 m; verbatimLatitude: 38°38'51''N; verbatimLongitude: 000°32'46''W; **Identification:** identifiedBy: F. J. Peris-Felipo; **Event:** samplingProtocol: Malaise trap; eventDate: 2007-06-25; **Record Level:** institutionCode: ENV
- **Type status:** Paratype. **Occurrence:** individualCount: 1; sex: female; **Location:** country: Spain; stateProvince: Castellón; locality: Pobal de Benifassà, Natural Park of Tinença de Benifassà; verbatimElevation: 662 m; verbatimLatitude: 38°38'51''N; verbatimLongitude: 000°32'46''W; **Identification:** identifiedBy: F. J. Peris-Felipo; **Event:** samplingProtocol: Malaise trap; eventDate: 2007-07-16; **Record Level:** institutionCode: ENV
- **Type status:** Paratype. **Occurrence:** individualCount: 1; sex: female; **Location:** country: Spain; stateProvince: Castellón; locality: Pobal de Benifassà, Natural Park of Tinença de Benifassà; verbatimElevation: 662 m; verbatimLatitude: 40°39'22''N; verbatimLongitude: 000°9'25''W; **Identification:** identifiedBy: F. J. Peris-Felipo; **Event:** samplingProtocol: Malaise trap; eventDate: 2004-06-10; **Record Level:** institutionCode: ENV
- **Type status:** Paratype. **Occurrence:** individualCount: 1; sex: female; **Location:** country: Spain; stateProvince: Castellón; locality: Pobal de Benifassà, Natural Park of Tinença de Benifassà; verbatimElevation: 662 m; verbatimLatitude: 40°39'22''N; verbatimLongitude: 000°9'25''W; **Identification:** identifiedBy: F. J. Peris-Felipo; **Event:** samplingProtocol: Malaise trap; eventDate: 2004-06-17; **Record Level:** institutionCode: ZISP
- **Type status:** Paratype. **Occurrence:** individualCount: 1; sex: male; **Location:** country: Spain; stateProvince: Castellón; locality: Pobal de Benifassà, Natural Park of Tinença de Benifassà; verbatimElevation: 662 m; verbatimLatitude: 40°39'22''N; verbatimLongitude: 000°9'25''W; **Identification:** identifiedBy: F. J. Peris-Felipo; **Event:** samplingProtocol: Malaise trap; eventDate: 2004-07-15; **Record Level:** institutionCode: ENV
- **Type status:** Paratype. **Occurrence:** individualCount: 2; sex: females; **Location:** country: Spain; stateProvince: Castellón; locality: Pobal de Benifassà, Natural Park of Tinença de Benifassà; verbatimElevation: 662 m; verbatimLatitude: 40°39'22''N; verbatimLongitude: 000°9'25''W; **Identification:** identifiedBy: F. J. Peris-Felipo; **Event:** samplingProtocol: Malaise trap; eventDate: 2004-07-22; **Record Level:** institutionCode: ENV
- **Type status:** Paratype. **Occurrence:** individualCount: 3; sex: females; **Location:** country: Spain; stateProvince: Castellón; locality: Pobal de Benifassà, Natural Park of Tinença de Benifassà; verbatimElevation: 662 m; verbatimLatitude: 40°39'22''N; verbatimLongitude: 000°9'25''W; **Identification:** identifiedBy: F. J. Peris-Felipo; **Event:** samplingProtocol: Malaise trap; eventDate: 2004-07-29; **Record Level:** institutionCode: ENV
- **Type status:** Paratype. **Occurrence:** individualCount: 1; sex: female; **Location:** country: Spain; stateProvince: Castellón; locality: Pobal de Benifassà, Natural Park of Tinença de Benifassà; verbatimElevation: 662 m; verbatimLatitude: 40°39'22''N; verbatimLongitude: 000°9'25''W; **Identification:** identifiedBy: F. J. Peris-Felipo; **Event:** samplingProtocol: Malaise trap; eventDate: 2004-08-05; **Record Level:** institutionCode: ENV
- **Type status:** Paratype. **Occurrence:** individualCount: 1; sex: female; **Location:** country: Spain; stateProvince: Castellón; locality: Pobal de Benifassà, Natural Park of Tinença de Benifassà; verbatimElevation: 662 m; verbatimLatitude: 40°39'22''N; verbatimLongitude: 000°9'25''W; **Identification:** identifiedBy: F. J. Peris-Felipo; **Event:** samplingProtocol: Malaise trap; eventDate: 2004-08-12; **Record Level:** institutionCode: ENV
- **Type status:** Paratype. **Occurrence:** individualCount: 1; sex: female; **Location:** country: Spain; stateProvince: Castellón; locality: Pobal de Benifassà, Natural Park of Tinença de Benifassà; verbatimElevation: 662 m; verbatimLatitude: 40°39'22''N; verbatimLongitude: 000°9'25''W; **Identification:** identifiedBy: F. J. Peris-Felipo; **Event:** samplingProtocol: Malaise trap; eventDate: 2004-09-16; **Record Level:** institutionCode: ZISP
- **Type status:** Paratype. **Occurrence:** individualCount: 2; sex: females; **Location:** country: Spain; stateProvince: Castellón; locality: Pobal de Benifassà, Natural Park of Tinença de Benifassà; verbatimElevation: 662 m; verbatimLatitude: 40°39'22''N; verbatimLongitude: 000°9'25''W; **Identification:** identifiedBy: F. J. Peris-Felipo; **Event:** samplingProtocol: Malaise trap; eventDate: 2004-09-23; **Record Level:** institutionCode: ENV
- **Type status:** Paratype. **Occurrence:** individualCount: 1; sex: female; **Location:** country: Spain; stateProvince: Castellón; locality: Pobal de Benifassà, Natural Park of Tinença de Benifassà; verbatimElevation: 662 m; verbatimLatitude: 40°39'22''N; verbatimLongitude: 000°9'25''W; **Identification:** identifiedBy: F. J. Peris-Felipo; **Event:** samplingProtocol: Malaise trap; eventDate: 2004-09-30; **Record Level:** institutionCode: ENV
- **Type status:** Paratype. **Occurrence:** individualCount: 1; sex: female; **Location:** country: Spain; stateProvince: Castellón; locality: Pobal de Benifassà, Natural Park of Tinença de Benifassà; verbatimElevation: 662 m; verbatimLatitude: 40°39'22''N; verbatimLongitude: 000°9'25''W; **Identification:** identifiedBy: F. J. Peris-Felipo; **Event:** samplingProtocol: Malaise trap; eventDate: 2004-10-07; **Record Level:** institutionCode: ENV
- **Type status:** Paratype. **Occurrence:** individualCount: 4; sex: females; **Location:** country: Spain; stateProvince: Castellón; locality: Pobal de Benifassà, Natural Park of Tinença de Benifassà; verbatimElevation: 662 m; verbatimLatitude: 40°39'22''N; verbatimLongitude: 000°9'25''W; **Identification:** identifiedBy: F. J. Peris-Felipo; **Event:** samplingProtocol: Malaise trap; eventDate: 2004-11-04; **Record Level:** institutionCode: ENV
- **Type status:** Paratype. **Occurrence:** individualCount: 5; sex: females; **Location:** country: Spain; stateProvince: Castellón; locality: Pobal de Benifassà, Natural Park of Tinença de Benifassà; verbatimElevation: 662 m; verbatimLatitude: 40°39'22''N; verbatimLongitude: 000°9'25''W; **Identification:** identifiedBy: F. J. Peris-Felipo; **Event:** samplingProtocol: Malaise trap; eventDate: 2005-06-13; **Record Level:** institutionCode: ENV
- **Type status:** Paratype. **Occurrence:** individualCount: 6; sex: females; **Location:** country: Spain; stateProvince: Castellón; locality: Pobal de Benifassà, Natural Park of Tinença de Benifassà; verbatimElevation: 662 m; verbatimLatitude: 40°39'22''N; verbatimLongitude: 000°9'25''W; **Identification:** identifiedBy: F. J. Peris-Felipo; **Event:** samplingProtocol: Malaise trap; eventDate: 2005-07-11; **Record Level:** institutionCode: ZISP
- **Type status:** Paratype. **Occurrence:** individualCount: 1; sex: female; **Location:** country: Spain; stateProvince: Castellón; locality: Pobal de Benifassà, Natural Park of Tinença de Benifassà; verbatimElevation: 662 m; verbatimLatitude: 40°39'22''N; verbatimLongitude: 000°9'25''W; **Identification:** identifiedBy: F. J. Peris-Felipo; **Event:** samplingProtocol: Malaise trap; eventDate: 2005-06-27; **Record Level:** institutionCode: ENV
- **Type status:** Paratype. **Occurrence:** individualCount: 1; sex: female; **Location:** country: Spain; stateProvince: Castellón; locality: Pobal de Benifassà, Natural Park of Tinença de Benifassà; verbatimElevation: 662 m; verbatimLatitude: 40°39'22''N; verbatimLongitude: 000°9'25''W; **Identification:** identifiedBy: F. J. Peris-Felipo; **Event:** samplingProtocol: Malaise trap; eventDate: 2005-07-04; **Record Level:** institutionCode: ENV
- **Type status:** Paratype. **Occurrence:** individualCount: 8; sex: females; **Location:** country: Spain; stateProvince: Castellón; locality: Pobal de Benifassà, Natural Park of Tinença de Benifassà; verbatimElevation: 662 m; verbatimLatitude: 40°39'22''N; verbatimLongitude: 000°9'25''W; **Identification:** identifiedBy: F. J. Peris-Felipo; **Event:** samplingProtocol: Malaise trap; eventDate: 2005-07-18; **Record Level:** institutionCode: NHMW
- **Type status:** Paratype. **Occurrence:** individualCount: 2; sex: females; **Location:** country: Spain; stateProvince: Castellón; locality: Pobal de Benifassà, Natural Park of Tinença de Benifassà; verbatimElevation: 662 m; verbatimLatitude: 40°39'22''N; verbatimLongitude: 000°9'25''W; **Identification:** identifiedBy: F. J. Peris-Felipo; **Event:** samplingProtocol: Malaise trap; eventDate: 2006-05-01; **Record Level:** institutionCode: ENV
- **Type status:** Paratype. **Occurrence:** individualCount: 1; sex: female; **Location:** country: Spain; stateProvince: Castellón; locality: Pobal de Benifassà, Natural Park of Tinença de Benifassà; verbatimElevation: 662 m; verbatimLatitude: 40°39'22''N; verbatimLongitude: 000°9'25''W; **Identification:** identifiedBy: F. J. Peris-Felipo; **Event:** samplingProtocol: Malaise trap; eventDate: 2006-05-08; **Record Level:** institutionCode: ENV
- **Type status:** Paratype. **Occurrence:** individualCount: 1; sex: male; **Location:** country: Spain; stateProvince: Castellón; locality: Pobal de Benifassà, Natural Park of Tinença de Benifassà; verbatimElevation: 662 m; verbatimLatitude: 40°39'22''N; verbatimLongitude: 000°9'25''W; **Identification:** identifiedBy: F. J. Peris-Felipo; **Event:** samplingProtocol: Malaise trap; eventDate: 2006-05-15; **Record Level:** institutionCode: ENV
- **Type status:** Paratype. **Occurrence:** individualCount: 6; sex: females; **Location:** country: Spain; stateProvince: Castellón; locality: Pobal de Benifassà, Natural Park of Tinença de Benifassà; verbatimElevation: 662 m; verbatimLatitude: 40°39'22''N; verbatimLongitude: 000°9'25''W; **Identification:** identifiedBy: F. J. Peris-Felipo; **Event:** samplingProtocol: Malaise trap; eventDate: 2006-05-15; **Record Level:** institutionCode: ENV
- **Type status:** Paratype. **Occurrence:** individualCount: 1; sex: male; **Location:** country: Spain; stateProvince: Castellón; locality: Pobal de Benifassà, Natural Park of Tinença de Benifassà; verbatimElevation: 662 m; verbatimLatitude: 40°39'22''N; verbatimLongitude: 000°9'25''W; **Identification:** identifiedBy: F. J. Peris-Felipo; **Event:** samplingProtocol: Malaise trap; eventDate: 2006-05-29; **Record Level:** institutionCode: HNHM
- **Type status:** Paratype. **Occurrence:** individualCount: 3; sex: females; **Location:** country: Spain; stateProvince: Castellón; locality: Pobal de Benifassà, Natural Park of Tinença de Benifassà; verbatimElevation: 662 m; verbatimLatitude: 40°39'22''N; verbatimLongitude: 000°9'25''W; **Identification:** identifiedBy: F. J. Peris-Felipo; **Event:** samplingProtocol: Malaise trap; eventDate: 2006-05-29; **Record Level:** institutionCode: ENV
- **Type status:** Paratype. **Occurrence:** individualCount: 1; sex: female; **Location:** country: Spain; stateProvince: Castellón; locality: Pobal de Benifassà, Natural Park of Tinença de Benifassà; verbatimElevation: 662 m; verbatimLatitude: 40°39'22''N; verbatimLongitude: 000°9'25''W; **Identification:** identifiedBy: F. J. Peris-Felipo; **Event:** samplingProtocol: Malaise trap; eventDate: 2006-06-05; **Record Level:** institutionCode: ENV
- **Type status:** Paratype. **Occurrence:** individualCount: 2; sex: females; **Location:** country: Spain; stateProvince: Castellón; locality: Pobal de Benifassà, Natural Park of Tinença de Benifassà; verbatimElevation: 662 m; verbatimLatitude: 40°39'22''N; verbatimLongitude: 000°9'25''W; **Identification:** identifiedBy: F. J. Peris-Felipo; **Event:** samplingProtocol: Malaise trap; eventDate: 2006-06-19; **Record Level:** institutionCode: ENV
- **Type status:** Paratype. **Occurrence:** individualCount: 1; sex: female; **Location:** country: Spain; stateProvince: Castellón; locality: Pobal de Benifassà, Natural Park of Tinença de Benifassà; verbatimElevation: 662 m; verbatimLatitude: 40°39'22''N; verbatimLongitude: 000°9'25''W; **Identification:** identifiedBy: F. J. Peris-Felipo; **Event:** samplingProtocol: Malaise trap; eventDate: 2006-06-26; **Record Level:** institutionCode: ENV
- **Type status:** Paratype. **Occurrence:** individualCount: 2; sex: females; **Location:** country: Spain; stateProvince: Alicante; locality: Torrevieja, Natural Park of Lagunas de La Mata-Torrevieja; verbatimElevation: 6 m; verbatimLatitude: 38°01'49''N; verbatimLongitude: 000°42'00''W; **Identification:** identifiedBy: F. J. Peris-Felipo; **Event:** samplingProtocol: Malaise trap; eventDate: 2007-04-23; **Record Level:** institutionCode: ENV
- **Type status:** Paratype. **Occurrence:** individualCount: 2; sex: females; **Location:** country: Spain; stateProvince: Alicante; locality: Torrevieja, Natural Park of Lagunas de La Mata-Torrevieja; verbatimElevation: 6 m; verbatimLatitude: 38°01'49''N; verbatimLongitude: 000°42'00''W; **Identification:** identifiedBy: F. J. Peris-Felipo; **Event:** samplingProtocol: Malaise trap; eventDate: 2004-05-04; **Record Level:** institutionCode: ENV
- **Type status:** Paratype. **Occurrence:** individualCount: 2; sex: females; **Location:** country: Spain; stateProvince: Alicante; locality: Torrevieja, Natural Park of Lagunas de La Mata-Torrevieja; verbatimElevation: 6 m; verbatimLatitude: 38°01'49''N; verbatimLongitude: 000°42'00''W; **Identification:** identifiedBy: F. J. Peris-Felipo; **Event:** samplingProtocol: Malaise trap; eventDate: 2004-05-11; **Record Level:** institutionCode: ENV
- **Type status:** Paratype. **Occurrence:** individualCount: 4; sex: females; **Location:** country: Spain; stateProvince: Alicante; locality: Torrevieja, Natural Park of Lagunas de La Mata-Torrevieja; verbatimElevation: 6 m; verbatimLatitude: 38°01'49''N; verbatimLongitude: 000°42'00''W; **Identification:** identifiedBy: F. J. Peris-Felipo; **Event:** samplingProtocol: Malaise trap; eventDate: 2004-05-18; **Record Level:** institutionCode: ZISP
- **Type status:** Paratype. **Occurrence:** individualCount: 7; sex: females; **Location:** country: Spain; stateProvince: Alicante; locality: Torrevieja, Natural Park of Lagunas de La Mata-Torrevieja; verbatimElevation: 6 m; verbatimLatitude: 38°01'49''N; verbatimLongitude: 000°42'00''W; **Identification:** identifiedBy: F. J. Peris-Felipo; **Event:** samplingProtocol: Malaise trap; eventDate: 2004-05-25; **Record Level:** institutionCode: ENV
- **Type status:** Paratype. **Occurrence:** individualCount: 1; sex: male; **Location:** country: Spain; stateProvince: Alicante; locality: Torrevieja, Natural Park of Lagunas de La Mata-Torrevieja; verbatimElevation: 6 m; verbatimLatitude: 38°01'49''N; verbatimLongitude: 000°42'00''W; **Identification:** identifiedBy: F. J. Peris-Felipo; **Event:** samplingProtocol: Malaise trap; eventDate: 2004-06-15; **Record Level:** institutionCode: ENV
- **Type status:** Paratype. **Occurrence:** individualCount: 2; sex: females; **Location:** country: Spain; stateProvince: Alicante; locality: Torrevieja, Natural Park of Lagunas de La Mata-Torrevieja; verbatimElevation: 6 m; verbatimLatitude: 38°01'49''N; verbatimLongitude: 000°42'00''W; **Identification:** identifiedBy: F. J. Peris-Felipo; **Event:** samplingProtocol: Malaise trap; eventDate: 2004-06-15; **Record Level:** institutionCode: ENV
- **Type status:** Paratype. **Occurrence:** individualCount: 1; sex: female; **Location:** country: Spain; stateProvince: Alicante; locality: Torrevieja, Natural Park of Lagunas de La Mata-Torrevieja; verbatimElevation: 6 m; verbatimLatitude: 38°01'49''N; verbatimLongitude: 000°42'00''W; **Identification:** identifiedBy: F. J. Peris-Felipo; **Event:** samplingProtocol: Malaise trap; eventDate: 2004-06-22; **Record Level:** institutionCode: ENV
- **Type status:** Paratype. **Occurrence:** individualCount: 2; sex: females; **Location:** country: Spain; stateProvince: Alicante; locality: Torrevieja, Natural Park of Lagunas de La Mata-Torrevieja; verbatimElevation: 6 m; verbatimLatitude: 38°01'49''N; verbatimLongitude: 000°42'00''W; **Identification:** identifiedBy: F. J. Peris-Felipo; **Event:** samplingProtocol: Malaise trap; eventDate: 2004-06-29; **Record Level:** institutionCode: ENV
- **Type status:** Paratype. **Occurrence:** individualCount: 1; sex: female; **Location:** country: Spain; stateProvince: Alicante; locality: Torrevieja, Natural Park of Lagunas de La Mata-Torrevieja; verbatimElevation: 6 m; verbatimLatitude: 38°01'49''N; verbatimLongitude: 000°42'00''W; **Identification:** identifiedBy: F. J. Peris-Felipo; **Event:** samplingProtocol: Malaise trap; eventDate: 2004-07-06; **Record Level:** institutionCode: ENV
- **Type status:** Paratype. **Occurrence:** individualCount: 1; sex: female; **Location:** country: Spain; stateProvince: Alicante; locality: Torrevieja, Natural Park of Lagunas de La Mata-Torrevieja; verbatimElevation: 6 m; verbatimLatitude: 38°01'49''N; verbatimLongitude: 000°42'00''W; **Identification:** identifiedBy: F. J. Peris-Felipo; **Event:** samplingProtocol: Malaise trap; eventDate: 2004-07-20; **Record Level:** institutionCode: ENV
- **Type status:** Paratype. **Occurrence:** individualCount: 3; sex: females; **Location:** country: Spain; stateProvince: Alicante; locality: Torrevieja, Natural Park of Lagunas de La Mata-Torrevieja; verbatimElevation: 6 m; verbatimLatitude: 38°01'49''N; verbatimLongitude: 000°42'00''W; **Identification:** identifiedBy: F. J. Peris-Felipo; **Event:** samplingProtocol: Malaise trap; eventDate: 2004-10-26; **Record Level:** institutionCode: ENV
- **Type status:** Paratype. **Occurrence:** individualCount: 1; sex: female; **Location:** country: Spain; stateProvince: Alicante; locality: Torrevieja, Natural Park of Lagunas de La Mata-Torrevieja; verbatimElevation: 6 m; verbatimLatitude: 38°01'49''N; verbatimLongitude: 000°42'00''W; **Identification:** identifiedBy: F. J. Peris-Felipo; **Event:** samplingProtocol: Malaise trap; eventDate: 2005-05-03; **Record Level:** institutionCode: ENV
- **Type status:** Paratype. **Occurrence:** individualCount: 2; sex: females; **Location:** country: Spain; stateProvince: Alicante; locality: Torrevieja, Natural Park of Lagunas de La Mata-Torrevieja; verbatimElevation: 6 m; verbatimLatitude: 38°01'49''N; verbatimLongitude: 000°42'00''W; **Identification:** identifiedBy: F. J. Peris-Felipo; **Event:** samplingProtocol: Malaise trap; eventDate: 2005-05-10; **Record Level:** institutionCode: NHMW
- **Type status:** Paratype. **Occurrence:** individualCount: 1; sex: female; **Location:** country: Spain; stateProvince: Alicante; locality: Torrevieja, Natural Park of Lagunas de La Mata-Torrevieja; verbatimElevation: 6 m; verbatimLatitude: 38°01'49''N; verbatimLongitude: 000°42'00''W; **Identification:** identifiedBy: F. J. Peris-Felipo; **Event:** samplingProtocol: Malaise trap; eventDate: 2005-05-17; **Record Level:** institutionCode: ENV
- **Type status:** Paratype. **Occurrence:** individualCount: 1; sex: female; **Location:** country: Spain; stateProvince: Alicante; locality: Torrevieja, Natural Park of Lagunas de La Mata-Torrevieja; verbatimElevation: 6 m; verbatimLatitude: 38°01'49''N; verbatimLongitude: 000°42'00''W; **Identification:** identifiedBy: F. J. Peris-Felipo; **Event:** samplingProtocol: Malaise trap; eventDate: 2005-06-14; **Record Level:** institutionCode: ENV
- **Type status:** Paratype. **Occurrence:** individualCount: 1; sex: female; **Location:** country: Spain; stateProvince: Alicante; locality: Torrevieja, Natural Park of Lagunas de La Mata-Torrevieja; verbatimElevation: 6 m; verbatimLatitude: 38°01'49''N; verbatimLongitude: 000°42'00''W; **Identification:** identifiedBy: F. J. Peris-Felipo; **Event:** samplingProtocol: Malaise trap; eventDate: 2005-07-13; **Record Level:** institutionCode: ENV
- **Type status:** Paratype. **Occurrence:** individualCount: 1; sex: female; **Location:** country: Spain; stateProvince: Alicante; locality: Torrevieja, Natural Park of Lagunas de La Mata-Torrevieja; verbatimElevation: 6 m; verbatimLatitude: 38°01'49''N; verbatimLongitude: 000°42'00''W; **Identification:** identifiedBy: F. J. Peris-Felipo; **Event:** samplingProtocol: Malaise trap; eventDate: 2005-10-30; **Record Level:** institutionCode: HNHM
- **Type status:** Paratype. **Occurrence:** individualCount: 1; sex: female; **Location:** country: Spain; stateProvince: Alicante; locality: Torrevieja, Natural Park of Lagunas de La Mata-Torrevieja; verbatimElevation: 6 m; verbatimLatitude: 38°01'49''N; verbatimLongitude: 000°42'00''W; **Identification:** identifiedBy: F. J. Peris-Felipo; **Event:** samplingProtocol: Malaise trap; eventDate: 2005-11-01; **Record Level:** institutionCode: ENV
- **Type status:** Paratype. **Occurrence:** individualCount: 2; sex: females; **Location:** country: Spain; stateProvince: Alicante; locality: Torrevieja, Natural Park of Lagunas de La Mata-Torrevieja; verbatimElevation: 6 m; verbatimLatitude: 38°01'49''N; verbatimLongitude: 000°42'00''W; **Identification:** identifiedBy: F. J. Peris-Felipo; **Event:** samplingProtocol: Malaise trap; eventDate: 2005-11-08; **Record Level:** institutionCode: ENV
- **Type status:** Paratype. **Occurrence:** individualCount: 1; sex: female; **Location:** country: Spain; stateProvince: Alicante; locality: Torrevieja, Natural Park of Lagunas de La Mata-Torrevieja; verbatimElevation: 6 m; verbatimLatitude: 38°01'49''N; verbatimLongitude: 000°42'00''W; **Identification:** identifiedBy: F. J. Peris-Felipo; **Event:** samplingProtocol: Malaise trap; eventDate: 2005-11-15; **Record Level:** institutionCode: ENV
- **Type status:** Paratype. **Occurrence:** individualCount: 2; sex: females; **Location:** country: Spain; stateProvince: Alicante; locality: Torrevieja, Natural Park of Lagunas de La Mata-Torrevieja; verbatimElevation: 6 m; verbatimLatitude: 38°01'49''N; verbatimLongitude: 000°42'00''W; **Identification:** identifiedBy: F. J. Peris-Felipo; **Event:** samplingProtocol: Malaise trap; eventDate: 2005-12-02; **Record Level:** institutionCode: ENV
- **Type status:** Paratype. **Occurrence:** individualCount: 1; sex: male; **Location:** country: Spain; stateProvince: Alicante; locality: Torrevieja, Natural Park of Lagunas de La Mata-Torrevieja; verbatimElevation: 6 m; verbatimLatitude: 38°01'49''N; verbatimLongitude: 000°42'00''W; **Identification:** identifiedBy: F. J. Peris-Felipo; **Event:** samplingProtocol: Malaise trap; eventDate: 2005-12-27; **Record Level:** institutionCode: ENV
- **Type status:** Paratype. **Occurrence:** individualCount: 1; sex: female; **Location:** country: Spain; stateProvince: Alicante; locality: Torrevieja, Natural Park of Lagunas de La Mata-Torrevieja; verbatimElevation: 6 m; verbatimLatitude: 38°01'49''N; verbatimLongitude: 000°42'00''W; **Identification:** identifiedBy: F. J. Peris-Felipo; **Event:** samplingProtocol: Malaise trap; eventDate: 2006-01-10; **Record Level:** institutionCode: ENV
- **Type status:** Paratype. **Occurrence:** individualCount: 1; sex: male; **Location:** country: Spain; stateProvince: Alicante; locality: Torrevieja, Natural Park of Lagunas de La Mata-Torrevieja; verbatimElevation: 6 m; verbatimLatitude: 38°01'49''N; verbatimLongitude: 000°42'00''W; **Identification:** identifiedBy: F. J. Peris-Felipo; **Event:** samplingProtocol: Malaise trap; eventDate: 2006-04-11; **Record Level:** institutionCode: ENV
- **Type status:** Paratype. **Occurrence:** individualCount: 2; sex: females; **Location:** country: Spain; stateProvince: Alicante; locality: Torrevieja, Natural Park of Lagunas de La Mata-Torrevieja; verbatimElevation: 6 m; verbatimLatitude: 38°01'49''N; verbatimLongitude: 000°42'00''W; **Identification:** identifiedBy: F. J. Peris-Felipo; **Event:** samplingProtocol: Malaise trap; eventDate: 2006-09-12; **Record Level:** institutionCode: ENV
- **Type status:** Paratype. **Occurrence:** individualCount: 5; sex: females; **Location:** country: Spain; stateProvince: Alicante; locality: Torrevieja, Natural Park of Lagunas de La Mata-Torrevieja; verbatimElevation: 6 m; verbatimLatitude: 38°01'49''N; verbatimLongitude: 000°42'00''W; **Identification:** identifiedBy: F. J. Peris-Felipo; **Event:** samplingProtocol: Malaise trap; eventDate: 2006-09-19; **Record Level:** institutionCode: ENV
- **Type status:** Paratype. **Occurrence:** individualCount: 1; sex: male; **Location:** country: Spain; stateProvince: Alicante; locality: Torrevieja, Natural Park of Lagunas de La Mata-Torrevieja; verbatimElevation: 6 m; verbatimLatitude: 38°01'49''N; verbatimLongitude: 000°42'00''W; **Identification:** identifiedBy: F. J. Peris-Felipo; **Event:** samplingProtocol: Malaise trap; eventDate: 2006-10-17; **Record Level:** institutionCode: ENV
- **Type status:** Paratype. **Occurrence:** individualCount: 2; sex: females; **Location:** country: Spain; stateProvince: Alicante; locality: Torrevieja, Natural Park of Lagunas de La Mata-Torrevieja; verbatimElevation: 6 m; verbatimLatitude: 38°01'49''N; verbatimLongitude: 000°42'00''W; **Identification:** identifiedBy: F. J. Peris-Felipo; **Event:** samplingProtocol: Malaise trap; eventDate: 2006-10-17; **Record Level:** institutionCode: ENV
- **Type status:** Paratype. **Occurrence:** individualCount: 1; sex: male; **Location:** country: Spain; stateProvince: Alicante; locality: Torrevieja, Natural Park of Lagunas de La Mata-Torrevieja; verbatimElevation: 6 m; verbatimLatitude: 38°01'49''N; verbatimLongitude: 000°42'00''W; **Identification:** identifiedBy: F. J. Peris-Felipo; **Event:** samplingProtocol: Malaise trap; eventDate: 2006-10-24; **Record Level:** institutionCode: ENV
- **Type status:** Paratype. **Occurrence:** individualCount: 1; sex: female; **Location:** country: Spain; stateProvince: Alicante; locality: Torrevieja, Natural Park of Lagunas de La Mata-Torrevieja; verbatimElevation: 6 m; verbatimLatitude: 38°01'49''N; verbatimLongitude: 000°42'00''W; **Identification:** identifiedBy: F. J. Peris-Felipo; **Event:** samplingProtocol: Malaise trap; eventDate: 2006-11-07; **Record Level:** institutionCode: ENV
- **Type status:** Paratype. **Occurrence:** individualCount: 2; sex: females; **Location:** country: Spain; stateProvince: Alicante; locality: Torrevieja, Natural Park of Lagunas de La Mata-Torrevieja; verbatimElevation: 6 m; verbatimLatitude: 38°01'49''N; verbatimLongitude: 000°42'00''W; **Identification:** identifiedBy: F. J. Peris-Felipo; **Event:** samplingProtocol: Malaise trap; eventDate: 2006-11-28; **Record Level:** institutionCode: ENV
- **Type status:** Paratype. **Occurrence:** individualCount: 3; sex: females; **Location:** country: Spain; stateProvince: Alicante; locality: Torrevieja, Natural Park of Lagunas de La Mata-Torrevieja; verbatimElevation: 6 m; verbatimLatitude: 38°01'49''N; verbatimLongitude: 000°42'00''W; **Identification:** identifiedBy: F. J. Peris-Felipo; **Event:** samplingProtocol: Malaise trap; eventDate: 2007-04-17; **Record Level:** institutionCode: ENV
- **Type status:** Paratype. **Occurrence:** individualCount: 3; sex: females; **Location:** country: Spain; stateProvince: Alicante; locality: Torrevieja, Natural Park of Lagunas de La Mata-Torrevieja; verbatimElevation: 6 m; verbatimLatitude: 38°01'49''N; verbatimLongitude: 000°42'00''W; **Identification:** identifiedBy: F. J. Peris-Felipo; **Event:** samplingProtocol: Malaise trap; eventDate: 2007-05-08; **Record Level:** institutionCode: ENV
- **Type status:** Paratype. **Occurrence:** individualCount: 1; sex: male; **Location:** country: Spain; stateProvince: Alicante; locality: Torrevieja, Natural Park of Lagunas de La Mata-Torrevieja; verbatimElevation: 6 m; verbatimLatitude: 38°01'49''N; verbatimLongitude: 000°42'00''W; **Identification:** identifiedBy: F. J. Peris-Felipo; **Event:** samplingProtocol: Malaise trap; eventDate: 2007-05-15; **Record Level:** institutionCode: ENV
- **Type status:** Paratype. **Occurrence:** individualCount: 1; sex: female; **Location:** country: Spain; stateProvince: Alicante; locality: Torrevieja, Natural Park of Lagunas de La Mata-Torrevieja; verbatimElevation: 6 m; verbatimLatitude: 38°01'49''N; verbatimLongitude: 000°42'00''W; **Identification:** identifiedBy: F. J. Peris-Felipo; **Event:** samplingProtocol: Malaise trap; eventDate: 2007-05-15; **Record Level:** institutionCode: ENV
- **Type status:** Paratype. **Occurrence:** individualCount: 1; sex: female; **Location:** country: Spain; stateProvince: Alicante; locality: Torrevieja, Natural Park of Lagunas de La Mata-Torrevieja; verbatimElevation: 6 m; verbatimLatitude: 38°01'49''N; verbatimLongitude: 000°42'00''W; **Identification:** identifiedBy: F. J. Peris-Felipo; **Event:** samplingProtocol: Malaise trap; eventDate: 2007-05-22; **Record Level:** institutionCode: NHMW
- **Type status:** Paratype. **Occurrence:** individualCount: 1; sex: female; **Location:** country: Spain; stateProvince: Alicante; locality: Torrevieja, Natural Park of Lagunas de La Mata-Torrevieja; verbatimElevation: 6 m; verbatimLatitude: 38°01'49''N; verbatimLongitude: 000°42'00''W; **Identification:** identifiedBy: F. J. Peris-Felipo; **Event:** samplingProtocol: Malaise trap; eventDate: 2007-05-29; **Record Level:** institutionCode: ENV
- **Type status:** Paratype. **Occurrence:** individualCount: 1; sex: female; **Location:** country: Spain; stateProvince: Alicante; locality: Torrevieja, Natural Park of Lagunas de La Mata-Torrevieja; verbatimElevation: 6 m; verbatimLatitude: 38°01'49''N; verbatimLongitude: 000°42'00''W; **Identification:** identifiedBy: F. J. Peris-Felipo; **Event:** samplingProtocol: Malaise trap; eventDate: 2007-06-05; **Record Level:** institutionCode: ENV
- **Type status:** Paratype. **Occurrence:** individualCount: 1; sex: female; **Location:** country: Spain; stateProvince: Alicante; locality: Torrevieja, Natural Park of Lagunas de La Mata-Torrevieja; verbatimElevation: 6 m; verbatimLatitude: 38°01'49''N; verbatimLongitude: 000°42'00''W; **Identification:** identifiedBy: F. J. Peris-Felipo; **Event:** samplingProtocol: Malaise trap; eventDate: 2007-06-19; **Record Level:** institutionCode: ENV
- **Type status:** Paratype. **Occurrence:** individualCount: 2; sex: females; **Location:** country: Spain; stateProvince: Alicante; locality: Torrevieja, Natural Park of Lagunas de La Mata-Torrevieja; verbatimElevation: 6 m; verbatimLatitude: 38°01'49''N; verbatimLongitude: 000°42'00''W; **Identification:** identifiedBy: F. J. Peris-Felipo; **Event:** samplingProtocol: Malaise trap; eventDate: 2007-07-03; **Record Level:** institutionCode: ENV
- **Type status:** Paratype. **Occurrence:** individualCount: 1; sex: female; **Location:** country: Spain; stateProvince: Alicante; locality: Torrevieja, Natural Park of Lagunas de La Mata-Torrevieja; verbatimElevation: 6 m; verbatimLatitude: 38°01'49''N; verbatimLongitude: 000°42'00''W; **Identification:** identifiedBy: F. J. Peris-Felipo; **Event:** samplingProtocol: Malaise trap; eventDate: 2007-10-16; **Record Level:** institutionCode: ENV

#### Distribution

Spain.

### Synaldis gilaberti

Peris-Felipo, 2014

#### Materials

- **Type status:** Holotype. **Occurrence:** individualCount: 1; sex: female; **Location:** country: Spain; stateProvince: Alicante; locality: Alcoi, Natural Park of Carrascal de La Font Roja; verbatimElevation: 1072 m; verbatimLatitude: 38°38'51''N; verbatimLongitude: 000°32'46''W; **Identification:** identifiedBy: F. J. Peris-Felipo; **Event:** samplingProtocol: Malaise trap; eventDate: 2004-11-01; **Record Level:** institutionCode: ENV
- **Type status:** Paratype. **Occurrence:** individualCount: 1; sex: female; **Location:** country: Spain; stateProvince: Castellón; locality: Pobla de Benifassà, Natural Park of Tinença de Benifassà; verbatimElevation: 662 m; verbatimLatitude: 40°39'22''N; verbatimLongitude: 000°9'25''W; **Identification:** identifiedBy: F. J. Peris-Felipo; **Event:** samplingProtocol: Malaise trap; eventDate: 2005-07-11; **Record Level:** institutionCode: ENV
- **Type status:** Paratype. **Occurrence:** individualCount: 1; sex: female; **Location:** country: Spain; stateProvince: Castellón; locality: Pobla de Benifassà, Natural Park of Tinença de Benifassà; verbatimElevation: 662 m; verbatimLatitude: 40°39'22''N; verbatimLongitude: 000°9'25''W; **Identification:** identifiedBy: F. J. Peris-Felipo; **Event:** samplingProtocol: Malaise trap; eventDate: 2005-07-18; **Record Level:** institutionCode: ENV

#### Distribution

Spain.

### Synaldis jujisae

Peris-Felipo, 2014

#### Materials

- **Type status:** Holotype. **Occurrence:** individualCount: 1; sex: female; **Location:** country: Spain; stateProvince: Alicante; locality: Alcoi, Natural Park of Carrascal de La Font Roja; verbatimElevation: 1072 m; verbatimLatitude: 38°38'51''N; verbatimLongitude: 000°32'46''W; **Identification:** identifiedBy: F. J. Peris-Felipo; **Event:** samplingProtocol: Malaise trap; eventDate: 2007-06-04; **Record Level:** institutionCode: ENV
- **Type status:** Paratype. **Occurrence:** individualCount: 1; sex: male; **Location:** country: Spain; stateProvince: Alicante; locality: Alcoi, Natural Park of Carrascal de La Font Roja; verbatimElevation: 1072 m; verbatimLatitude: 38°38'51''N; verbatimLongitude: 000°32'46''W; **Identification:** identifiedBy: F. J. Peris-Felipo; **Event:** samplingProtocol: Malaise trap; eventDate: 2005-09-12; **Record Level:** institutionCode: ENV
- **Type status:** Paratype. **Occurrence:** individualCount: 1; sex: female; **Location:** country: Spain; stateProvince: Alicante; locality: Alcoi, Natural Park of Carrascal de La Font Roja; verbatimElevation: 1072 m; verbatimLatitude: 38°38'51''N; verbatimLongitude: 000°32'46''W; **Identification:** identifiedBy: F. J. Peris-Felipo; **Event:** samplingProtocol: Malaise trap; eventDate: 2006-07-17; **Record Level:** institutionCode: ENV
- **Type status:** Paratype. **Occurrence:** individualCount: 1; sex: male; **Location:** country: Spain; stateProvince: Alicante; locality: Alcoi, Natural Park of Carrascal de La Font Roja; verbatimElevation: 1072 m; verbatimLatitude: 38°38'51''N; verbatimLongitude: 000°32'46''W; **Identification:** identifiedBy: F. J. Peris-Felipo; **Event:** samplingProtocol: Malaise trap; eventDate: 2006-09-25; **Record Level:** institutionCode: ENV
- **Type status:** Paratype. **Occurrence:** individualCount: 1; sex: male; **Location:** country: Spain; stateProvince: Alicante; locality: Alcoi, Natural Park of Carrascal de La Font Roja; verbatimElevation: 1072 m; verbatimLatitude: 38°38'51''N; verbatimLongitude: 000°32'46''W; **Identification:** identifiedBy: F. J. Peris-Felipo; **Event:** samplingProtocol: Malaise trap; eventDate: 2006-10-23; **Record Level:** institutionCode: ENV
- **Type status:** Paratype. **Occurrence:** individualCount: 1; sex: male; **Location:** country: Spain; stateProvince: Alicante; locality: Alcoi, Natural Park of Carrascal de La Font Roja; verbatimElevation: 1072 m; verbatimLatitude: 38°38'51''N; verbatimLongitude: 000°32'46''W; **Identification:** identifiedBy: F. J. Peris-Felipo; **Event:** samplingProtocol: Malaise trap; eventDate: 2007-07-02; **Record Level:** institutionCode: ZISP
- **Type status:** Paratype. **Occurrence:** individualCount: 1; sex: male; **Location:** country: Spain; stateProvince: Alicante; locality: Alcoi, Natural Park of Carrascal de La Font Roja; verbatimElevation: 1072 m; verbatimLatitude: 38°38'51''N; verbatimLongitude: 000°32'46''W; **Identification:** identifiedBy: F. J. Peris-Felipo; **Event:** samplingProtocol: Malaise trap; eventDate: 2007-07-30; **Record Level:** institutionCode: ENV

#### Distribution

Spain.

### Synaldis lacessiva

Fischer, 1975

#### Materials

- **Type status:** Other material. **Occurrence:** individualCount: 1; sex: female; **Location:** country: Spain; stateProvince: Alicante; locality: Alcoi, Natural Park of Carrascal de La Font Roja; verbatimElevation: 1072 m; verbatimLatitude: 38°38'51''N; verbatimLongitude: 000°32'46''W; **Identification:** identifiedBy: F. J. Peris-Felipo; **Event:** samplingProtocol: Malaise trap; eventDate: 2004-05-27; **Record Level:** institutionCode: ENV
- **Type status:** Other material. **Occurrence:** individualCount: 1; sex: female; **Location:** country: Spain; stateProvince: Alicante; locality: Alcoi, Natural Park of Carrascal de La Font Roja; verbatimElevation: 1072 m; verbatimLatitude: 38°38'51''N; verbatimLongitude: 000°32'46''W; **Identification:** identifiedBy: F. J. Peris-Felipo; **Event:** samplingProtocol: Malaise trap; eventDate: 2004-06-03; **Record Level:** institutionCode: ENV
- **Type status:** Other material. **Occurrence:** individualCount: 1; sex: female; **Location:** country: Spain; stateProvince: Alicante; locality: Alcoi, Natural Park of Carrascal de La Font Roja; verbatimElevation: 1072 m; verbatimLatitude: 38°38'51''N; verbatimLongitude: 000°32'46''W; **Identification:** identifiedBy: F. J. Peris-Felipo; **Event:** samplingProtocol: Malaise trap; eventDate: 2004-06-12; **Record Level:** institutionCode: ENV
- **Type status:** Other material. **Occurrence:** individualCount: 1; sex: female; **Location:** country: Spain; stateProvince: Alicante; locality: Alcoi, Natural Park of Carrascal de La Font Roja; verbatimElevation: 1072 m; verbatimLatitude: 38°38'51''N; verbatimLongitude: 000°32'46''W; **Identification:** identifiedBy: F. J. Peris-Felipo; **Event:** samplingProtocol: Malaise trap; eventDate: 2004-06-24; **Record Level:** institutionCode: ENV
- **Type status:** Other material. **Occurrence:** individualCount: 3; sex: females; **Location:** country: Spain; stateProvince: Alicante; locality: Alcoi, Natural Park of Carrascal de La Font Roja; verbatimElevation: 1072 m; verbatimLatitude: 38°38'51''N; verbatimLongitude: 000°32'46''W; **Identification:** identifiedBy: F. J. Peris-Felipo; **Event:** samplingProtocol: Malaise trap; eventDate: 2004-07-01; **Record Level:** institutionCode: ENV
- **Type status:** Other material. **Occurrence:** individualCount: 6; sex: females; **Location:** country: Spain; stateProvince: Alicante; locality: Alcoi, Natural Park of Carrascal de La Font Roja; verbatimElevation: 1072 m; verbatimLatitude: 38°38'51''N; verbatimLongitude: 000°32'46''W; **Identification:** identifiedBy: F. J. Peris-Felipo; **Event:** samplingProtocol: Malaise trap; eventDate: 2004-07-08; **Record Level:** institutionCode: ENV
- **Type status:** Other material. **Occurrence:** individualCount: 2; sex: females; **Location:** country: Spain; stateProvince: Alicante; locality: Alcoi, Natural Park of Carrascal de La Font Roja; verbatimElevation: 1072 m; verbatimLatitude: 38°38'51''N; verbatimLongitude: 000°32'46''W; **Identification:** identifiedBy: F. J. Peris-Felipo; **Event:** samplingProtocol: Malaise trap; eventDate: 2004-07-22; **Record Level:** institutionCode: ENV
- **Type status:** Other material. **Occurrence:** individualCount: 1; sex: female; **Location:** country: Spain; stateProvince: Alicante; locality: Alcoi, Natural Park of Carrascal de La Font Roja; verbatimElevation: 1072 m; verbatimLatitude: 38°38'51''N; verbatimLongitude: 000°32'46''W; **Identification:** identifiedBy: F. J. Peris-Felipo; **Event:** samplingProtocol: Malaise trap; eventDate: 2004-07-29; **Record Level:** institutionCode: ENV
- **Type status:** Other material. **Occurrence:** individualCount: 1; sex: female; **Location:** country: Spain; stateProvince: Alicante; locality: Alcoi, Natural Park of Carrascal de La Font Roja; verbatimElevation: 1072 m; verbatimLatitude: 38°38'51''N; verbatimLongitude: 000°32'46''W; **Identification:** identifiedBy: F. J. Peris-Felipo; **Event:** samplingProtocol: Malaise trap; eventDate: 2004-09-27; **Record Level:** institutionCode: ENV
- **Type status:** Other material. **Occurrence:** individualCount: 1; sex: female; **Location:** country: Spain; stateProvince: Alicante; locality: Alcoi, Natural Park of Carrascal de La Font Roja; verbatimElevation: 1072 m; verbatimLatitude: 38°38'51''N; verbatimLongitude: 000°32'46''W; **Identification:** identifiedBy: F. J. Peris-Felipo; **Event:** samplingProtocol: Malaise trap; eventDate: 2004-11-01; **Record Level:** institutionCode: ENV
- **Type status:** Other material. **Occurrence:** individualCount: 2; sex: females; **Location:** country: Spain; stateProvince: Alicante; locality: Alcoi, Natural Park of Carrascal de La Font Roja; verbatimElevation: 1072 m; verbatimLatitude: 38°38'51''N; verbatimLongitude: 000°32'46''W; **Identification:** identifiedBy: F. J. Peris-Felipo; **Event:** samplingProtocol: Malaise trap; eventDate: 2005-05-16; **Record Level:** institutionCode: ENV
- **Type status:** Other material. **Occurrence:** individualCount: 1; sex: female; **Location:** country: Spain; stateProvince: Alicante; locality: Alcoi, Natural Park of Carrascal de La Font Roja; verbatimElevation: 1072 m; verbatimLatitude: 38°38'51''N; verbatimLongitude: 000°32'46''W; **Identification:** identifiedBy: F. J. Peris-Felipo; **Event:** samplingProtocol: Malaise trap; eventDate: 2005-05-23; **Record Level:** institutionCode: ENV
- **Type status:** Other material. **Occurrence:** individualCount: 1; sex: female; **Location:** country: Spain; stateProvince: Alicante; locality: Alcoi, Natural Park of Carrascal de La Font Roja; verbatimElevation: 1072 m; verbatimLatitude: 38°38'51''N; verbatimLongitude: 000°32'46''W; **Identification:** identifiedBy: F. J. Peris-Felipo; **Event:** samplingProtocol: Malaise trap; eventDate: 2005-10-17; **Record Level:** institutionCode: ENV
- **Type status:** Other material. **Occurrence:** individualCount: 1; sex: female; **Location:** country: Spain; stateProvince: Alicante; locality: Alcoi, Natural Park of Carrascal de La Font Roja; verbatimElevation: 1072 m; verbatimLatitude: 38°38'51''N; verbatimLongitude: 000°32'46''W; **Identification:** identifiedBy: F. J. Peris-Felipo; **Event:** samplingProtocol: Malaise trap; eventDate: 2006-06-12; **Record Level:** institutionCode: ENV
- **Type status:** Other material. **Occurrence:** individualCount: 1; sex: male; **Location:** country: Spain; stateProvince: Alicante; locality: Alcoi, Natural Park of Carrascal de La Font Roja; verbatimElevation: 1072 m; verbatimLatitude: 38°38'51''N; verbatimLongitude: 000°32'46''W; **Identification:** identifiedBy: F. J. Peris-Felipo; **Event:** samplingProtocol: Malaise trap; eventDate: 2006-07-17; **Record Level:** institutionCode: ENV
- **Type status:** Other material. **Occurrence:** individualCount: 1; sex: female; **Location:** country: Spain; stateProvince: Alicante; locality: Alcoi, Natural Park of Carrascal de La Font Roja; verbatimElevation: 1072 m; verbatimLatitude: 38°38'51''N; verbatimLongitude: 000°32'46''W; **Identification:** identifiedBy: F. J. Peris-Felipo; **Event:** samplingProtocol: Malaise trap; eventDate: 2006-08-14; **Record Level:** institutionCode: ENV
- **Type status:** Other material. **Occurrence:** individualCount: 2; sex: females; **Location:** country: Spain; stateProvince: Alicante; locality: Alcoi, Natural Park of Carrascal de La Font Roja; verbatimElevation: 1072 m; verbatimLatitude: 38°38'51''N; verbatimLongitude: 000°32'46''W; **Identification:** identifiedBy: F. J. Peris-Felipo; **Event:** samplingProtocol: Malaise trap; eventDate: 2006-08-21; **Record Level:** institutionCode: ENV
- **Type status:** Other material. **Occurrence:** individualCount: 1; sex: female; **Location:** country: Spain; stateProvince: Alicante; locality: Alcoi, Natural Park of Carrascal de La Font Roja; verbatimElevation: 1072 m; verbatimLatitude: 38°38'51''N; verbatimLongitude: 000°32'46''W; **Identification:** identifiedBy: F. J. Peris-Felipo; **Event:** samplingProtocol: Malaise trap; eventDate: 2006-09-18; **Record Level:** institutionCode: ENV
- **Type status:** Other material. **Occurrence:** individualCount: 1; sex: female; **Location:** country: Spain; stateProvince: Alicante; locality: Alcoi, Natural Park of Carrascal de La Font Roja; verbatimElevation: 1072 m; verbatimLatitude: 38°38'51''N; verbatimLongitude: 000°32'46''W; **Identification:** identifiedBy: F. J. Peris-Felipo; **Event:** samplingProtocol: Malaise trap; eventDate: 2006-11-13; **Record Level:** institutionCode: ENV
- **Type status:** Other material. **Occurrence:** individualCount: 2; sex: females; **Location:** country: Spain; stateProvince: Alicante; locality: Alcoi, Natural Park of Carrascal de La Font Roja; verbatimElevation: 1072 m; verbatimLatitude: 38°38'51''N; verbatimLongitude: 000°32'46''W; **Identification:** identifiedBy: F. J. Peris-Felipo; **Event:** samplingProtocol: Malaise trap; eventDate: 2007-06-04; **Record Level:** institutionCode: ENV
- **Type status:** Other material. **Occurrence:** individualCount: 1; sex: male; **Location:** country: Spain; stateProvince: Alicante; locality: Alcoi, Natural Park of Carrascal de La Font Roja; verbatimElevation: 1072 m; verbatimLatitude: 38°38'51''N; verbatimLongitude: 000°32'46''W; **Identification:** identifiedBy: F. J. Peris-Felipo; **Event:** samplingProtocol: Malaise trap; eventDate: 2007-06-04; **Record Level:** institutionCode: ENV
- **Type status:** Other material. **Occurrence:** individualCount: 4; sex: females; **Location:** country: Spain; stateProvince: Alicante; locality: Alcoi, Natural Park of Carrascal de La Font Roja; verbatimElevation: 1072 m; verbatimLatitude: 38°38'51''N; verbatimLongitude: 000°32'46''W; **Identification:** identifiedBy: F. J. Peris-Felipo; **Event:** samplingProtocol: Malaise trap; eventDate: 2007-06-11; **Record Level:** institutionCode: ENV
- **Type status:** Other material. **Occurrence:** individualCount: 1; sex: female; **Location:** country: Spain; stateProvince: Alicante; locality: Alcoi, Natural Park of Carrascal de La Font Roja; verbatimElevation: 1072 m; verbatimLatitude: 38°38'51''N; verbatimLongitude: 000°32'46''W; **Identification:** identifiedBy: F. J. Peris-Felipo; **Event:** samplingProtocol: Malaise trap; eventDate: 2007-06-18; **Record Level:** institutionCode: ENV
- **Type status:** Other material. **Occurrence:** individualCount: 1; sex: male; **Location:** country: Spain; stateProvince: Alicante; locality: Alcoi, Natural Park of Carrascal de La Font Roja; verbatimElevation: 1072 m; verbatimLatitude: 38°38'51''N; verbatimLongitude: 000°32'46''W; **Identification:** identifiedBy: F. J. Peris-Felipo; **Event:** samplingProtocol: Malaise trap; eventDate: 2007-06-18; **Record Level:** institutionCode: ENV
- **Type status:** Other material. **Occurrence:** individualCount: 1; sex: male; **Location:** country: Spain; stateProvince: Alicante; locality: Alcoi, Natural Park of Carrascal de La Font Roja; verbatimElevation: 1072 m; verbatimLatitude: 38°38'51''N; verbatimLongitude: 000°32'46''W; **Identification:** identifiedBy: F. J. Peris-Felipo; **Event:** samplingProtocol: Malaise trap; eventDate: 2007-07-02; **Record Level:** institutionCode: ENV
- **Type status:** Other material. **Occurrence:** individualCount: 1; sex: female; **Location:** country: Spain; stateProvince: Alicante; locality: Alcoi, Natural Park of Carrascal de La Font Roja; verbatimElevation: 1072 m; verbatimLatitude: 38°38'51''N; verbatimLongitude: 000°32'46''W; **Identification:** identifiedBy: F. J. Peris-Felipo; **Event:** samplingProtocol: Malaise trap; eventDate: 2007-07-16; **Record Level:** institutionCode: ENV
- **Type status:** Other material. **Occurrence:** individualCount: 1; sex: female; **Location:** country: Spain; stateProvince: Castellón; locality: Pobla de Benifassà, Natural Park of Tinença de Benifassà; verbatimElevation: 662 m; verbatimLatitude: 40°39'22''N; verbatimLongitude: 000°9'25''W; **Identification:** identifiedBy: F. J. Peris-Felipo; **Event:** samplingProtocol: Malaise trap; eventDate: 2004-05-27; **Record Level:** institutionCode: ENV

#### Distribution

Austria, Canary Islands, Czech Republic, Hungary, Madeira Islands and Spain.

### Synaldis lozanoae

Peris-Felipo, 2014

#### Materials

- **Type status:** Holotype. **Occurrence:** individualCount: 1; sex: female; **Location:** country: Spain; stateProvince: Castellón; locality: Pobla de Benifassà, Natural Park of Tinença de Benifassà; verbatimElevation: 662 m; verbatimLatitude: 40°39'22''N; verbatimLongitude: 000°9'25''W; **Identification:** identifiedBy: F. J. Peris-Felipo; **Event:** samplingProtocol: Malaise trap; eventDate: 2005-05-23; **Record Level:** institutionCode: ENV
- **Type status:** Paratype. **Occurrence:** individualCount: 1; sex: female; **Location:** country: Spain; stateProvince: Castellón; locality: Pobla de Benifassà, Natural Park of Tinença de Benifassà; verbatimElevation: 662 m; verbatimLatitude: 40°39'22''N; verbatimLongitude: 000°9'25''W; **Identification:** identifiedBy: F. J. Peris-Felipo; **Event:** samplingProtocol: Malaise trap; eventDate: 2005-05-31; **Record Level:** institutionCode: ENV
- **Type status:** Paratype. **Occurrence:** individualCount: 1; sex: female; **Location:** country: Spain; stateProvince: Castellón; locality: Pobla de Benifassà, Natural Park of Tinença de Benifassà; verbatimElevation: 662 m; verbatimLatitude: 40°39'22''N; verbatimLongitude: 000°9'25''W; **Identification:** identifiedBy: F. J. Peris-Felipo; **Event:** samplingProtocol: Malaise trap; eventDate: 2005-06-13; **Record Level:** institutionCode: ENV
- **Type status:** Paratype. **Occurrence:** individualCount: 1; sex: female; **Location:** country: Spain; stateProvince: Castellón; locality: Pobla de Benifassà, Natural Park of Tinença de Benifassà; verbatimElevation: 662 m; verbatimLatitude: 40°39'22''N; verbatimLongitude: 000°9'25''W; **Identification:** identifiedBy: F. J. Peris-Felipo; **Event:** samplingProtocol: Malaise trap; eventDate: 2005-07-25; **Record Level:** institutionCode: ENV
- **Type status:** Paratype. **Occurrence:** individualCount: 1; sex: female; **Location:** country: Spain; stateProvince: Castellón; locality: Pobla de Benifassà, Natural Park of Tinença de Benifassà; verbatimElevation: 662 m; verbatimLatitude: 40°39'22''N; verbatimLongitude: 000°9'25''W; **Identification:** identifiedBy: F. J. Peris-Felipo; **Event:** samplingProtocol: Malaise trap; eventDate: 2006-06-12; **Record Level:** institutionCode: ZISP
- **Type status:** Paratype. **Occurrence:** individualCount: 1; sex: male; **Location:** country: Spain; stateProvince: Castellón; locality: Pobla de Benifassà, Natural Park of Tinença de Benifassà; verbatimElevation: 662 m; verbatimLatitude: 40°39'22''N; verbatimLongitude: 000°9'25''W; **Identification:** identifiedBy: F. J. Peris-Felipo; **Event:** samplingProtocol: Malaise trap; eventDate: 2007-02-05; **Record Level:** institutionCode: ENV
- **Type status:** Paratype. **Occurrence:** individualCount: 1; sex: male; **Location:** country: Spain; stateProvince: Alicante; locality: Alcoi, Natural Park of Carrascal de La Font Roja; verbatimElevation: 1072 m; verbatimLatitude: 38°38'51''N; verbatimLongitude: 000°32'46''W; **Identification:** identifiedBy: F. J. Peris-Felipo; **Event:** samplingProtocol: Malaise trap; eventDate: 2007-05-14; **Record Level:** institutionCode: ENV

#### Distribution

Spain.

### Synaldis martinezae

Peris-Felipo, 2014

#### Materials

- **Type status:** Holotype. **Occurrence:** individualCount: 1; sex: female; **Location:** country: Spain; stateProvince: Alicante; locality: Torrevieja, Natural Park of Lagunas de La Mata-Torrevieja; verbatimElevation: 6 m; verbatimLatitude: 38°01'49''N; verbatimLongitude: 000°42'00''W; **Identification:** identifiedBy: F. J. Peris-Felipo; **Event:** samplingProtocol: Malaise trap; eventDate: 2004-05-25; **Record Level:** institutionCode: ENV
- **Type status:** Paratype. **Occurrence:** individualCount: 1; sex: female; **Location:** country: Spain; stateProvince: Alicante; locality: Torrevieja, Natural Park of Lagunas de La Mata-Torrevieja; verbatimElevation: 6 m; verbatimLatitude: 38°01'49''N; verbatimLongitude: 000°42'00''W; **Identification:** identifiedBy: F. J. Peris-Felipo; **Event:** samplingProtocol: Malaise trap; eventDate: 2004-06-22; **Record Level:** institutionCode: ENV
- **Type status:** Paratype. **Occurrence:** individualCount: 1; sex: female; **Location:** country: Spain; stateProvince: Alicante; locality: Torrevieja, Natural Park of Lagunas de La Mata-Torrevieja; verbatimElevation: 6 m; verbatimLatitude: 38°01'49''N; verbatimLongitude: 000°42'00''W; **Identification:** identifiedBy: F. J. Peris-Felipo; **Event:** samplingProtocol: Malaise trap; eventDate: 2004-10-12; **Record Level:** institutionCode: ENV
- **Type status:** Paratype. **Occurrence:** individualCount: 1; sex: female; **Location:** country: Spain; stateProvince: Alicante; locality: Torrevieja, Natural Park of Lagunas de La Mata-Torrevieja; verbatimElevation: 6 m; verbatimLatitude: 38°01'49''N; verbatimLongitude: 000°42'00''W; **Identification:** identifiedBy: F. J. Peris-Felipo; **Event:** samplingProtocol: Malaise trap; eventDate: 2004-10-12; **Record Level:** institutionCode: ZISP
- **Type status:** Paratype. **Occurrence:** individualCount: 1; sex: female; **Location:** country: Spain; stateProvince: Alicante; locality: Torrevieja, Natural Park of Lagunas de La Mata-Torrevieja; verbatimElevation: 6 m; verbatimLatitude: 38°01'49''N; verbatimLongitude: 000°42'00''W; **Identification:** identifiedBy: F. J. Peris-Felipo; **Event:** samplingProtocol: Malaise trap; eventDate: 2006-09-19; **Record Level:** institutionCode: ENV

#### Distribution

Spain.

### Synaldis navarroae

Peris-Felipo, 2014

#### Materials

- **Type status:** Holotype. **Occurrence:** individualCount: 1; sex: female; **Location:** country: Spain; stateProvince: Alicante; locality: Torrevieja, Natural Park of Lagunas de La Mata-Torrevieja; verbatimElevation: 6 m; verbatimLatitude: 38°01'49''N; verbatimLongitude: 000°42'00''W; **Identification:** identifiedBy: F. J. Peris-Felipo; **Event:** samplingProtocol: Malaise trap; eventDate: 2005-02-02; **Record Level:** institutionCode: ENV
- **Type status:** Paratype. **Occurrence:** individualCount: 1; sex: male; **Location:** country: Spain; stateProvince: Alicante; locality: Torrevieja, Natural Park of Lagunas de La Mata-Torrevieja; verbatimElevation: 6 m; verbatimLatitude: 38°01'49''N; verbatimLongitude: 000°42'00''W; **Identification:** identifiedBy: F. J. Peris-Felipo; **Event:** samplingProtocol: Malaise trap; eventDate: 2005-03-18; **Record Level:** institutionCode: ENV

#### Distribution

Spain.

## Analysis

During the sampling period, 1707 specimens of Alysiinae were collected, including 822 specimens in the *Aspilota*-group (48.23%) distributed in 6 genera: *Adelphenaldis* (2), *Aspilota* (108), *Dinotrema* (343), *Eudinostigma* (10), *Orthostigma* (88) and *Synaldis* (271) and representing 53 species. The list of the species with collecting information was given above.

However, the species were not evenly distributed when different Natural Parks are considered separately. Thus, 39 species were identified in the Natural Park of La Tinença de Benifassà (Tinença), 23 were identified in the Natural Park of Carrascal de La Font Roja (Font Roja) and 21 were identified in the Natural Park of Las Lagunas de la Mata-Torrevieja (Torrevieja).

The genus *Dinotrema* was the most abundant with 343 examples, followed by the genera *Synaldis* (271) and *Aspilota* (108). On the other hand, when analysing the number of captures, it was observed that 383 individuals were collected in Tinença, 257 in Torrevieja and 182 in Font Roja. In Tinença the most captured genera was *Dinotrema* with 202 specimens followed by *Synaldis* with 95. However, in Torrevieja and Font Roja the most captured genera was *Synaldis* with 105 and 71 specimens respectively, followed by *Dinotrema* with 93 and 48.

The Margalef index (D*_Mg_*) shows that Natural Park of Tinença hosted a higher species richness with D*_Mg_* = 6.389, while Font Roja reached a value of 4.228 and Torrevieja 3.604. These values might be so discordant as a consequence of the identified species differing widely from Tinença (39 species) to other habitats. Font Roja and Torrevieja has a similar D*_Mg_* value because its species number is very close (23 and 21 species respectively).

On the other hand, with the estimators of species richness (Chao 2), it is possible conclude that the Natural Park where our sampling effort has enabled a closer approximation to the estimated maximum richness is Font Roja with a value of 94.62%, followed by Tinença and Torrevieja with values of 82.97% and 79.75% respectively.

When analyzing the structure of the community it is needed to distinguish between two types of analysis: proportional abundance indices or parametric models.

First, the community structure is studied by proportional abundance indices in which differentiate dominance indices as Simpson or Berger-Parker and equity index as Shannon-Wiener.

The results obtained with the Simpson and Berger-Parker index (Table 1) show a dominance of the community structure by one or more species with high population abundance. The Shannon index suggested a similar trend in the distribution of dominant genera; discrepancies were merely due to different numbers of rare genera (those represented by few specimens).

Finally, applying parametric models, (Table 2) the analysis of the *Aspilota*-group community structure showed that Font Roja and Torrevieja present compliance with the *log-series* model indicating that these communities have an unstable structure, composed by few abundant species and a large number of rare species. These results show that habitat does not determine community structure because the sampling area presents very specific botanical and faunal composition and climatic conditions.

However, Tinença shows compliance with *log-series* and *log-normal* models presenting, more or less, the same p-value (0.501 and 0.513 respectively). This fact could be indicating two types of behaviour. On the one hand, this community could be unstable and composed by few abundant species and large number of rare species. And, on the other hand, it could be indicating that the specimens number of this community is conditioned by a large number of factors associated with high temperatures and low rainfall that occur in this area causing that species must adapt to very strict conditions.

**Figure 1.**
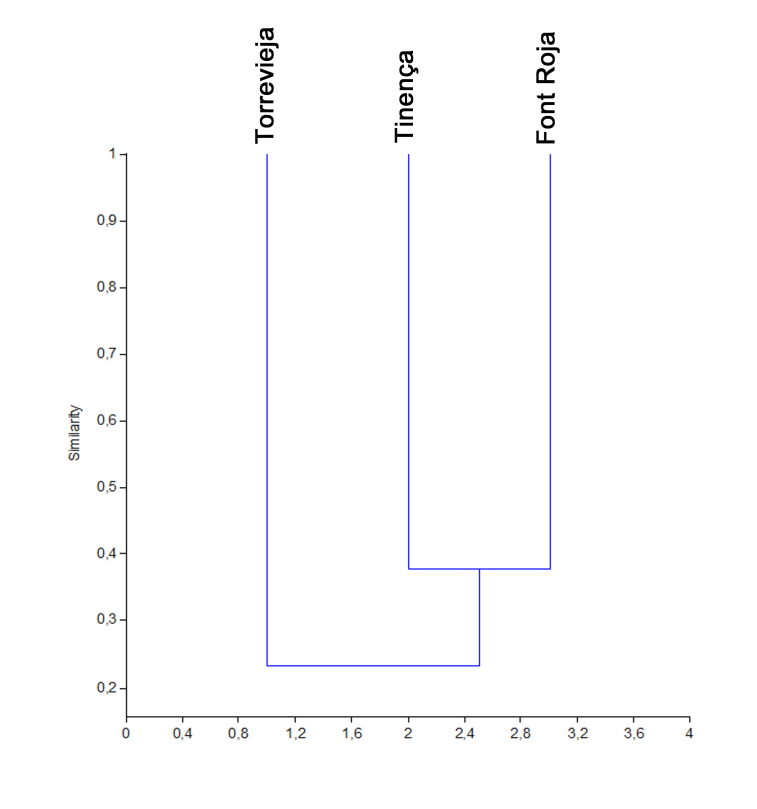
Cluster of Jaccard analysis reflecting relationship between Natural Parks.

In order to obtain beta diversity (similarity/dissimilarity) values between the different areas under consideration, the Jaccard index was calculated. The resulting value indicated a certain degree of dissimilarity between the Natural Parks although, Font Roja and Tinença are the closest parks (I_J_ = 0.377) while Font Roja and Torrevieja are the farthest parks (I_J_ = 0.189). These results were also observed in the Jaccard cluster obtained through cluster analysis, of which the level of correlation was *r* = 0.8863 (Fig. 1).

**Figure 2.**
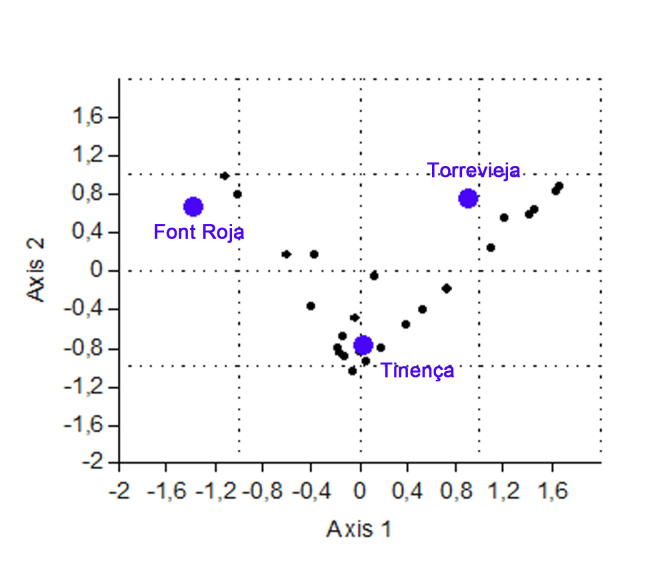
Principal Component Analysis (PCA).

However, applying the Principal Component Analysis (PCA) (Fig. 2) shows that there are many unique species to each Natural Park (16 for Tinença, 7 for Torrevieja and 6 for Font Roja) while the rest of species are usually present shared (17 for Font Roja-Tinença, 12 for Tinença-Torrevieja and 7 for Font Roja-Torrevieja). This could be due to the fact that Tinença and Font Roja are Mediterranean forests while Torrevieja is a lagoon.

The indices of species replacement by the Whittaker index (Table 3) show that the Natural Park of La Tinença de Benifassà has not a lot of replacement with species from other Natural Parks, while, Torrevieja and Font Roja show some replacement. This relationship could be possible thanks that these natural parks are close while Tinença is far.

Applying the changes proposed by Schluter and Ricklefs (1993) get the average beta diversity then employ for gamma diversity.

β = 1 / c = 1 / average number of samples occupied per one species.

c = Σ number of species in each area / number of species

c = [23 + 39 + 21] / 53 = 83 / 53 = 1.566

β = 1 / 1.566 = 0.638

The Complementarity index (C) suggested that the Font Roja and Torrevieja has the highest complementarity (0.810) followed by Tinença and Torrevieja with 0.723 and Tinença and Font Roja with 0.622. These results showed a fair degree of complementarity, but also indicated the presence of different species in each habitat (Table 3). This fact could be explained because these natural parks are close to each other while Tinença is farther apart.

Finally, gamma diversity reached a value of 52.954, which is practically identical to the value of the total species richness caught in the three Natural Parks (species number = 53).

γ = α average × β × sample dimension

γ = [(23 + 39 + 21) / (3)] × 0.638 × 3 = 52.954

## Discussion

Regarding the faunistic study, four species captured are new records for Spain: *Aspilota delicata*, *Aspilota procreata*, *Dinotrema costulatum* and *Dinotrema crassicostum*. While, regarding the biodiversity study, it is possible to see that the Natural Park of La Tinença de Benifassà presents greater abundance and species diversity, followed by the Font Roja and Torrevieja parks. On the other hand, the analysis of the structure of the network has showed that the Font Roja and the Torrevieja Natural Parks show a model of community that matches the *log-series* model. This indicates that these communities are unstable and are composed of few abundant species and a large number of rare species. While the community of the *Aspilota*-group present in the Tinença, is adapted to the models of *log-series* and *log-normal*. This demonstrates that the structure of the community is not determined by the habitat, but conditioned by a large number of factors associated with the high temperatures and low rates of precipitation, which may force the species to adapt to strict environmental conditions. Furthermore, when comparing parks, it can be seen that La Tinença and La Font Roja show the most similarities between each other, whilst the Font Roja Park and the Torrevieja Park show a larger group of species that complement each other.

On the other hand, checking with the studies realized in other areas of Spain as Artikutza about the *Aspilota*-group show that this group was the most abundant captured with approximately 75.77% (Peris-Felipo and Jiménez-Peydró 2011). The information about the abundance is very interesting due to the relationships that these parasitic wasps have with their hosts. This information could be used to estimate the biodiversity appearing in each area.

Finally, we conclude that, although this study was conducted to determine the diversity and community structure of the *Aspilota*-group, it is recommended further studies of Braconidae in different areas together with DNA-barcode studies to increase the knowledge of this large group that still remains largely unknown.

## Supplementary Material

XML Treatment for Adelphenaldis maxfischeri

XML Treatment for Aspilota anaphoretica

XML Treatment for Aspilota delicata

XML Treatment for Aspilota flagimilis

XML Treatment for Aspilota insolita

XML Treatment for Aspilota procreata

XML Treatment for Aspilota propedaemon

XML Treatment for Aspilota propeminimam

XML Treatment for Aspilota valenciensis

XML Treatment for Aspilota sp1

XML Treatment for Aspilota sp2

XML Treatment for Dinotrema achterbergi

XML Treatment for Dinotrema amparoae

XML Treatment for Dinotrema belokobylskiji

XML Treatment for Dinotrema benifassaense

XML Treatment for Dinotrema broadi

XML Treatment for Dinotrema castaneithorax

XML Treatment for Dinotrema costulatum

XML Treatment for Dinotrema crassicosta

XML Treatment for Dinotrema enanum

XML Treatment for Dinotrema fischerianum

XML Treatment for Dinotrema jimenezi

XML Treatment for Dinotrema lagunasense

XML Treatment for Dinotrema mareum

XML Treatment for Dinotrema munki

XML Treatment for Dinotrema pappi

XML Treatment for Dinotrema paquitae

XML Treatment for Dinotrema parapunctatum

XML Treatment for Dinotrema pareum

XML Treatment for Dinotrema pilarae

XML Treatment for Dinotrema robertoi

XML Treatment for Dinotrema teresae

XML Treatment for Dinotrema tinencaense

XML Treatment for Dinotrema torreviejaense

XML Treatment for Dinotrema vitobiasi

XML Treatment for Dinotrema zimmermannae

XML Treatment for Eudinostigma latistigma

XML Treatment for Orthostigma beyarslani

XML Treatment for Orthostigma laticeps

XML Treatment for Orthostigma maculipes

XML Treatment for Orthostigma pumilum

XML Treatment for Orthostigma sculpturatum

XML Treatment for Synaldis berbegalae

XML Treatment for Synaldis concolor

XML Treatment for Synaldis distracta

XML Treatment for Synaldis falcoi

XML Treatment for Synaldis gilaberti

XML Treatment for Synaldis jujisae

XML Treatment for Synaldis lacessiva

XML Treatment for Synaldis lozanoae

XML Treatment for Synaldis martinezae

XML Treatment for Synaldis navarroae

## Figures and Tables

**Table 1.** Expected frequency of species (exp f) according to abundance models (log-series, log-normal and broken-stick) for the *Aspilota*-group community.

	Font Roja	Tinença	Torrevieja
Species	23	39	21
Specimens	182	383	257
Simpson I.	0.835	0.919	0.798
Berger-Parker	0.274	0.185	0.307
Shannon I.	2.234	3.006	2.023

**Table 2.** Expected frequency of species (exp f) according to abundance models (log-series, log-normal and broken-stick) for the *Aspilota*-group community.

	Font Roja						Tinença						Torrevieja					
	*Log-series*		*Log-normal*		*Broken-stick*		*Log-series*		*Log-normal*		*Broken-stick*		*Log-series*		*Log-normal*		*Broken-stick*	
Class	Exp f	Obs f	Exp f	Obs f	Exp f	Obs f	Exp f	Obs f	Exp f	Obs f	Exp f	Obs f	Exp f	Obs f	Exp f	Obs f	Exp f	Obs f
0	–	–	5.72	0	–	–	–	–	1.72	0	–	–	–	–	3.12	0	–	–
1	9.94	14	17.55	14	4.68	14	15.69	14	10.95	14	6.70	14	7.89	10	5.63	10	2.92	10
2	3.57	3	7.75	3	3.70	3	5.75	4	6.66	4	5.51	4	2.94	4	2.28	4	2.52	4
3	3.49	2	6.83	2	5.22	2	5.78	6	7.03	6	8.26	6	3.01	1	2.19	1	4.03	1
4	2.94	0	4.72	0	5.18	0	5.14	10	5.54	10	9.30	10	2.79	2	1.74	2	5.14	2
5	1.97	2	2.55	2	2.53	2	3.83	3	3.29	3	5.97	3	2.25	2	1.14	2	4.16	2
6	0.87	2	1.01	2	0.30	2	2.08	1	1.41	1	1.30	1	1.42	0	0.64	0	1.34	0
7	0.18	0	0.32	0	0.00	0	0.63	1	0.46	1	0.03	1	0.57	2	0.31	2	0.06	2
8	0.09	0	0.10	0	0.00	0	0.06	0	0.09	0	0.00	0	0.10	0	0.12	0	0.00	0
9	–	–	–	–	–	–	0.00	0	0.04	0	0.00	0	0.00	0	0.06	0	0.00	0
	X^2^ = 6.973		X^2^ = 18.994		X^2^ = 35.603		X^2^ = 6.336		X^2^ = 8.202		X^2^ = 41.949		X^2^ = 7.647		X^2^ = 19.177		X^2^ = 87.420	
	p = 0.323		p = 0.014		p = 0.000		p = 0.501		p = 0.513		p = 0.000		p = 0.364		p = 0.023		p = 0.000	

**Table 3.** Whittaker index and Complementarity index values for *Aspilota*-group between Natural Parks.

	Tinença	Font Roja	Torrevieja	
**Tinença**		0.622	0.723	**Complementarity**
**Font Roja**	0.709		0.810	
**Torrevieja**	0.766	1.409		
	**Whittaker**			
